# Vocal complexity and sociality in spotted paca (*Cuniculus paca*)

**DOI:** 10.1371/journal.pone.0190961

**Published:** 2018-01-24

**Authors:** Stella G. C. Lima, Renata S. Sousa-Lima, Rosana S. Tokumaru, Sérgio L. G. Nogueira-Filho, Selene S. C. Nogueira

**Affiliations:** 1 Laboratório de Bioacústica, Departamento de Fisiologia e Comportamento / Programa de Pós-Graduação em Psicobiologia, Universidade Federal do Rio Grande do Norte, Natal, RN, Brazil; 2 Departamento de Psicologia Social e do Desenvolvimento, Universidade Federal do Espírito Santo, Vitória, ES, Brazil; 3 Laboratório de Etologia Aplicada, Departamento de Ciências Biológicas, Universidade Estadual de Santa Cruz, Ilhéus, BA, Brazil; University of Windsor, CANADA

## Abstract

The evolution of sociality is related to many ecological factors that act on animals as selective forces, thus driving the formation of groups. Group size will depend on the payoffs of group living. The Social Complexity Hypothesis for Communication (SCHC) predicts that increases in group size will be related to increases in the complexity of the communication among individuals. This hypothesis, which was confirmed in some mammal societies, may be useful to trace sociality in the spotted paca (*Cuniculus paca*), a Neotropical caviomorph rodent reported as solitary. There are, however, sightings of groups in the wild, and farmers easily form groups of spotted paca in captivity. Thus, we aimed to describe the acoustic repertoire of captive spotted paca to test the SCHC and to obtain insights about the sociability of this species. Moreover, we aimed to verify the relationship between group size and acoustic repertoire size of caviomorph rodents, to better understand the evolution of sociality in this taxon. We predicted that spotted paca should display a complex acoustic repertoire, given their social behavior in captivity and group sightings in the wild. We also predicted that in caviomorph species the group size would increase with acoustic repertoire, supporting the SCHC. We performed a Linear Discriminant Analysis (LDA) based on acoustic parameters of the vocalizations recorded. In addition, we applied an independent contrasts approach to investigate sociality in spotted paca following the social complexity hypothesis, independent of phylogeny. Our analysis showed that the spotted paca’s acoustic repertoire contains seven vocal types and one mechanical signal. The broad acoustic repertoire of the spotted paca might have evolved given the species’ ability to live in groups. The relationship between group size and the size of the acoustic repertoires of caviomorph species was confirmed, providing additional support for the SCHC in yet another group of diverse mammals–caviomorph rodents.

## Introduction

Comparative ecology and phylogeny analyses have been applied [1] to better understand the relationships between the variation in species’ social behavior and evolutionary history [2]. The evolution of sociality or group living [3] has been extensively investigated in many mammal species to answer many questions including links between sociality and communication [2], and is related to increased predation risk and vigilance [4], diurnal habits [5], body size [6], collective defense [7], burrowing [5, 8], sexual selection [9], mating system [10], food resources [11], habitat [12], and many other ecological factors (for a review, see [2] and references therein). In caviomorph rodents, for instance, it has been suggested that sociality coevolved with increasing body sizes and use of burrows [8].

It is not only natural events that influence the size of social groups in mammals [13]; species group living has also been driven by human-induced rapid environmental change (HIREC) *sensu* [14, 15], such as climate change, habitat fragmentation, and hunting. These anthropogenic factors have challenged species to adapt to persist in a modified environment [14–16]. Therefore, the observation of the behavior of individuals of a given species in human disturbed environments can be misleading and may change under different ecological conditions. This plasticity is particularly evident in caviomorph rodents. Intraspecific variation in spacing, group size, and mating systems in response to many factors including predation, have been reported for this group [17]. The capybara (*Hydrochoerus hydrochaeris*), for instance, is known to change its behavior in response to environmental pressure, becoming more nocturnal in areas with high hunting pressure [18].

Plastic behavioral traits, such as communicative signals, may respond rapidly to environmental change. In fact, communication and cognition are social traits that differ greatly between social and non-social mammals and therefore, could contribute to understanding the evolution of sociality [2]. Acoustic communication is involved in social bonding, predator defense, cooperative foraging, reproduction, and dominance hierarchy negotiations, to name but a few (for a complete review, see [2]. Complexity in social relationships drives the evolutionary paths of social cognition and communication in parallel to each other [19], as different signals mediate several subtle processes.

Complex communication modulates behavioral responses and is expected to be present in more complex societies [20]. The Social Complexity Hypothesis for Communication (SCHC), states that solitary animals show a lower diversity of communicative signals than species that live in groups with complex social systems [21]. This hypothesis has been tested in some societies of mammals: ground-dwelling sciurid rodents [22], bats [23], non-human primates [24], whales [25], giant otter [26], meerkats, and slender mongooses [27]. In sciurids, for instance, it was reported that different attributes of sociality are linked to different attributes of communication [28]. Therefore, the SCHC may explain the evolution of sociality in caviomorph rodents, which have a relatively resolved phylogeny. Additional information about the acoustic repertoire of the spotted paca (*Cuniculus paca*) may reveal a different level of sociality that otherwise would not be detected due to HIREC *sensu* [14, 15].

The spotted paca is the second largest rodent occurring from Southern Mexico to Northern Argentina, and it is widely distributed in Neotropical countries [29]. The species is nocturnal, burrower [30–32], and shows moderate sociality [1], mostly reported as solitary, living in monogamous pairs during reproductive periods [30–32]. Nevertheless, there are some anecdotal reports of free-ranging spotted paca living in groups of five individuals [33–35]. Moreover, farmers easily form groups of paca by gathering post-weaning juveniles to form captive breeding groups [34, 36]. The acoustic repertoire of spotted paca has been qualitatively described and is composed of six types of acoustic emissions, most of them related to contact (low grunt and low whine), alarm (snort and low growl), and aggressiveness (tooth chattering and very loud growl) [37, 38]. However, no data are available on the acoustic parameters of these calls. Thus, we aimed to extend the knowledge on the spotted paca’s acoustic repertoire by describing its acoustic structure and to evaluate if the repertoire of this species adheres to the SCHC predictions. Complex communicative systems predict larger vocal repertoires or features that increase the variability in vocal signals, such as combinations of sounds [39] and formants [40], promoting more efficient information transfer in social groups [41]. We then, aimed to verify the relationship between group size and the vocal repertoire size of caviomorph rodents, to better understand the evolution of sociality in this taxon. Due to reports of spotted paca’s social-living [33–35], we hypothesized that the vocal repertoire of this species would show higher complexity in its repertoire size or signal features. Considering the variation of social behavior, social-living and vocal repertoire sizes among caviomorph species, we predicted this group would provide compelling evidence of the positive relationship between the number of call types and group size (SCHC). We also predicted that the size of the vocal repertoire of the spotted paca or its complexity would correspond to those of group-living caviomorph species.

## Methods

### Ethics statement

This work followed the principles of laboratory animal care (NIH publication No. 86–23, revised in 1985) and was approved by the Committee on Animal Research and Ethics of the State University of Santa Cruz, under protocol # 010/11.

### Study area and subjects

The study was carried out on a farm where spotted paca are commercially raised, namely Empreendimentos Agropecuários e Obras S/A (EAO), zip code 42841-000, at the town of Camaçari, state of Bahia, Brazil. We recorded vocalizations and behaviors of 51 individuals: 42 adults (26 females and 16 males) and nine juveniles (six females and three males). All individuals were born and raised in captivity. The age of adults ranged from two to four years old and the age of juveniles ranged from 15 days to four months old. Unfortunately, there were no sub-adult animals available to improve our samples. Animals were housed in groups of one male and four females in 20 breeding pens, and four maternity pens with one female and its offspring. Five out of 20 breeding pens had one to two juveniles.

Each pen, including maternity pens, occupied 6 m^2^ (3.0m long x 2.0m wide), with concrete floor, surrounded by a 2.0m-high wire mesh fence supported by wooden poles. The ceiling was covered with tiles. All pens had two wooden shelters (1.5m long x 1.5m high x 1.0m wide), a water tank (0.6m long x 0.3m wide) and two feeders (1.0m long x 0.3m wide). During observations at night, we used red lights to allow the visualization of animals and to minimize disturbances. Rabbit commercial pelleted feed (200 g per animal) plus seasonal fruits and mineral salt *ad libitum* were daily furnished. Water was also offered *ad libitum*.

### Data collection

Both calls and behaviors were recorded simultaneously at 1.5 m distance (*in maximum*) from the animals. The observation sessions usually took place between 4.00pm and 6.00am, the period of the highest activity of the captive spotted paca. We also observed the animals on three other routine occasions: during cleaning of the pen (between 11.00am and 1.00pm), during handling for medical procedures such as parasites control and weighing of animals (between 11.00am and 1.00pm), and when the animals were fed (4.00pm).

We recorded the acoustic signs *ad libitum* [42] between March and April 2014 using a Sennheiser ME-66 directional microphone (Wedemark, Germany) and a Marantz PMD 670 (Sagamihara, Japan) digital recorder (recorder settings: WAV format, mono mode, 48 kHz sampling rate, and 16-bits resolution). The observer started to record the animals when they were actively emitting sounds and kept recording until any sound emission was produced up to its end or after an interval of 1 minute with no sound emission. The animals were not marked individually; however, it was possible to identify them by natural characteristics. Because the animals are not very vocal, we could not register calls from all individuals housed in the breeding pens. The data collection totalized 90 hours of recording and observations. Just one observer (SGCL) collected and analyzed all behavioral and acoustic data. Two other authors (SLGNF and SSCN) analyzed a subset of the data from this study and using the Kendall’s coefficient of concordance we found inter-observer reliability in coding of these vocalizations (W ≥ 0.77).

To stimulate the emission of isolation or contact calls between a mother and her young, we isolated four mothers from their offspring by using a wooden barrier (0.12m long X 0.75m high X 0.02m wide), which acted as a barrier to physical and visual signals, but did not imply the loss of auditory and olfactory contact. The animals’ keeper was responsible for setting up the barrier between mothers and offspring. The observer started to record the vocalizations just after the keeper isolated the animals. Each mother/pup pair was separated from each other only once. This recording/observation session lasted 30 minutes per pair.

### Acoustic analysis

Spectrograms and oscillograms were generated and analyzed with Raven Pro Software version 1.5 (Cornell Lab of Ornithology, Ithaca NY) using the same settings for all call types: Hann window, 1460 window length, 90% overlap, and 4096 FFT size. First, we categorized putative call types by ear and by visual inspection of spectrograms and oscillograms. The smaller vocal units of the calls were termed elements and were defined as a continuous sound without interruption *sensu* [43, 44]. For each element, we measured the following parameters: minimum frequency (Hz), maximum frequency (Hz), dominant frequency (Hz), number of harmonics under 1 kHz, and call duration (s) (S1 Table). Sound parameters were measured from spectrograms, oscillograms, and power spectra, and these were used to describe and compare vocalizations. Oscillograms were used to measure signal duration. Spectrograms and power spectrum were used to measure the minimum, maximum frequencies of the sound and the dominant frequency of the selection. Minimum frequency is the lowest frequency boundary and maximum frequency is the upper frequency boundary of the selection [45]. Dominant frequency is the frequency that corresponds to maximum power occurrence within the element selection [45]. To calculate call emission rate per hour (hourly rates) we divided the total numbers of each vocal type by the total data collection (90 h).

We also examined spectrograms for the presence of vocal complexity through structural variability, such as combination of sounds (combination of different vocalization types in sequences) [39], and formants (vocal tract resonance frequencies) [46]. To analyze the combination of sounds of *roar* and *groan* vocalizations (S2 Table), we measured the number of elements, the order of elements, the duration of the sequence (s), and the rhythm of the sequence (the number of elements divided by the total sequence duration). To test whether the pronounced horizontal frequency bands visible in roars spectrograms were formants, we compared measured and predicted ‘formant dispersion’, which is the averaged difference between successive formants [47]. For this, we obtained four adult 1-2- year-old male spotted pacas (4.6 ± 0.7 kg) that were destined to be sent to the abattoir at the EAO farm. This part of the study was carried out at the Laboratory of Applied Ethology, Universidade Estadual de Santa Cruz, Ilhéus, Bahia, Brazil. At this site, we individually introduced the animals into cages (1.2 m long X 0.8 m high X 0.8 m wide). The cages were made of metal with a feeder, a drinking trough, and a wooden shelter. After 15 days of habituation to the new conditions, we recorded the acoustic signs *ad libitum* [42] between August and September 2017, using the same procedures and equipment as described above. *Roars* were induced by showing to the individuals the handling net. Then we selected 40 roars (10 calls/animal) (S3 Table) and measured the values of the first five formant frequencies using linear predictive coding (LPC: ‘To Formants: Burg method’) command in PRAAT software, version 5.3.06 [48]. The following settings were used: time step: 0.01 s; maximum number of formants: 6; maximum formant value: 11000 Hz; window length: 0.01 s; pre-emphasis: 50 Hz. Each element was measured separately and them we measured the averaged formant dispersion using the equation:

Df=∑(Fi+1–Fi)/N–1 (1)

where *D*f is the formant dispersion (in Hz) and *N* is the total number of formants measured.

We then returned animals to the commercial farm and, after their slaughter, which followed ethical and legal rules; we performed anatomical measurements of the vocal tracts. The vocal tract length (*VTL*) was determined with a tape measure from the middle of the glottis to the front of the incisors (cm). According to Fitch [47], the measurement of *VTL* should be associated with formant frequency dispersion, as it follows the path of plane-wave propagation of sound, in a ‘uniform tube’ (a tube that does not vary in cross-sectional area), from the glottis to the oral or nasal radiation site, and the frequency difference between the successive resonances is constant and given by the equation:

Fi–Fi–1=c/2VTL (2)

where *i* is the formant number, *c* is the speed of sound (350 m s^−1^), *VTL* is vocal tract length (in m) and *F*_i_ is the frequency (in Hz) of the *i*th formant.

Following Ried and Fitch [46] we determined the average of the obtained differences to provide an overall estimate of spectral dispersion of each of the animals. We used the standard deviation of the formant intervals to evaluate the extent to which the uniform tube approximation holds [46].

### Statistical analysis

To test the validity of the putative call types that we categorized by ear and by visual inspection of the spectrograms, we conducted a Linear Discriminant Analysis (LDA) based on four of the five acoustic parameters that were measured. We excluded the number of harmonics under 1 kHz in LDA because this parameter showed no variation. We randomly chose 50 elements of each vocal type with high signal quality (low background noise and no overlap among calls from different individuals) from the total 3,341 elements recorded for acoustic measurements, totaling 400 samples (S1 Table). Elements were taken from different recording sections to increase the chances of analyzing different vocal types and calls from different individuals. Moreover, to avoid over-sampling, we randomly selected the data by considering both call types (from three to nine calls per individual) and individuals. We first randomly selected elements for each vocal type from different recording sections, and the individuals were balanced in these samples to increase the chances of analyzing different vocal types from different individuals (see Table 1). Before conducting the LDA analysis we standardized the variables (by subtracting the mean of the variable from each data point and dividing the result by the variable’s standard deviation–the Z score transformation) to avoid the spurious attribution of weights to acoustic parameters measured in different units [49]. To test the significance of the discriminant model we performed a Multivariate Analysis of Variance (MANOVA). Further, to determine whether it was possible to predict each vocal type correctly based on the measured acoustic parameters (independent variables), we performed cross-validation analyses using the ‘leave one sample out’ procedure [50], in which each case was classified by the functions derived from all cases other than the one being classified, reporting the LDA accuracy as the proportion of elements correctly assigned to each vocal type. We performed a binomial test to evaluate whether the proportion of the success of the cross-validation analysis was higher than that expected by chance (12.5%).

**Table 1:** Mean ± standard deviation (SD) of acoustic parameters measured in spotted paca vocalizations. Percentage of notes correctly attributed to each call type in the cross validation. The N corresponds to the number of emissions analyzed in each category/number of individuals from which individual calls were selected. The centroids show the spacing of different call types of spotted paca in a two-dimensional signal space defined by the first two discriminant functions (DF1 and DF2). The values in bold indicate the correlation coefficients of the variables that most contributed to the discriminant functions (DF1 and DF2).

Vocal type	N	Duration (s)	Dominant frequency (Hz)	Minimum frequency (Hz)	Maximum frequency (Hz)	Cross- validation	Centroids	
							DF1	DF2
Roar	50/21	1.5 ± 0.9	672.0 ± 589.0	95.2 ± 66.2	15922.8 ± 8712.8	68.0%	-1.41	-1.21
Growl	50/14	0.4 ± 0.2	392.1 ± 242.7	114.9 ± 110.1	13577.3 ± 9173.9	34.0%	0.67	0.18
Bark	50/15	0.2 ± 0.07	531.8 ± 348.6	107.5 ± 68.9	15466.6 ± 7321.6	62.0%	0.78	0.59
Tooth chattering	50/11	0.09 ± 0.0	438.0 ± 157.2	264.3 ± 124.5	13960.6 ± 6858.3	92.0%	0.51	1.71
Groan	50/7	0.5 ± 0.2	359.8 ± 191.7	162.5 ± 96.4	5782.4 ± 6814.7	52.0%	0.42	-0.15
Snore	50/18	0.3 ± 0.05	190.8 ± 82.0	65.1 ± 44.2	8702.4 ± 7892.4	48.0%	1.29	-0.48
Click	50/7	0.2 ± 0.05	146.0 ± 28.3	47.8 ± 16.7	2863.4 ± 3820.3	86.0%	1.55	-0.92
Cry	50/5	1.8 ± 0.7	1676.9 ± 711.4	280.8 ± 139.5	14434.7 ± 4979.6	84.0%	-3.81	0.27
DF1	-	**-0.90**	**-0.87**	-0.49	-0.32	-		
DF2	-	-0.40	-0.19	**-0.72**	-0.33	-		

Additionally, we ran a General Linear Model (GLM), considering call types and age class as predictors, to verify potential differences between juveniles and adults shared call types. We first calculated the individual’s averages for each acoustic measurement per call type, and then used these means as the dependent variable in the analysis, and the call type as the independent variable. Furthermore, we used the agglomerative hierarchical cluster analysis to investigate whether the combinations of *roar* and *groan* would fit into additional call types (S2 Table). This method successively links the most similar objects into larger groups. Average values of duration, number of notes, and rhythm of the different combinations were used as input data. Euclidean distances were used as the measurement of distance between the pair of situations and Ward’s criterion was the linkage criterion. Ward’s criterion minimizes the within-cluster variation. Furthermore, we used the automatic truncation option, based on the entropy and tries, to create homogeneous groups.

We used the paired t-test to compare the predicted and measured formant dispersions of the four spotted pacas for which we obtained both anatomical and acoustic data (S3 Table). For this analysis we used the average formant dispersion (*D*f—measured formant dispersion) determined from vocalisation recordings using Eq 1 and the theoretical values calculated using the *VTL* obtained and Eq 2 (predicted formant dispersion). All analyses were performed using XLSTAT (Version 2017.4, Addinsoft), with a significance level of *α <* .05.

### Vocal complexity and group size: Phylogenetically based analysis

We determined the relationship between group size and vocal repertoire size by means of independent contrasts analyses. For this, we used the total repertoire size of the selected caviomorph species (see results section), considering only adult repertoire to avoid ontogeny effects. Although different research groups characterized possibly similar call types with different terminology, we assumed that authors, independent of the analyses chosen, discriminated these species repertoire as the maximum. Thus, we used the independent contrasts approach [51, 52] to investigate sociality in spotted paca following the social complexity hypothesis [21], independent of phylogeny.

We used the topology described by Ebensperger and Blumstein [5] to establish major relationships among seven caviomorph rodent species, from which we had information on average group sizes and on the repertoire size of adult vocalizations based on acoustic parameter information. The positions of *Spalacopus cyanus* and *Ctenomys talarum* were based on [53]. To calculate independent contrasts, we defined sociality as the log_10_ of the midpoint of the range of observed group sizes, as the midpoint is an appropriate measure of central tendency, given a wide range of values within a species, following [5] Our continuous independent variables were the log_10_ of the adult vocal repertoire reported for each species, excluding mechanical sounds and juvenile vocalizations. We transformed mid group size and vocal repertoire to eliminate outliers and to meet assumptions of linear models. As the phylogeny used did not have consistently good estimates of the branch lengths model of evolution we assumed the Nee transformation [54], where the distance from the tips to the focal node was calculated by log_10_ transforming the number of tips descending from that node. Given these phylogenies, we calculated independent contrasts and determined the relationship between independent contrasts of group size and independent contrasts of vocal repertoire size, in order to investigate sociality in spotted paca following the SCHC [21] through a linear regression analysis. We then used the obtained equation to estimate the group size of the spotted paca through its vocal repertoire. In the linear regression analysis, we also used XLSTAT (Version 2017.4, Addinsoft) with a significance level of *α <* .05.

## Results

**Fig 1:**
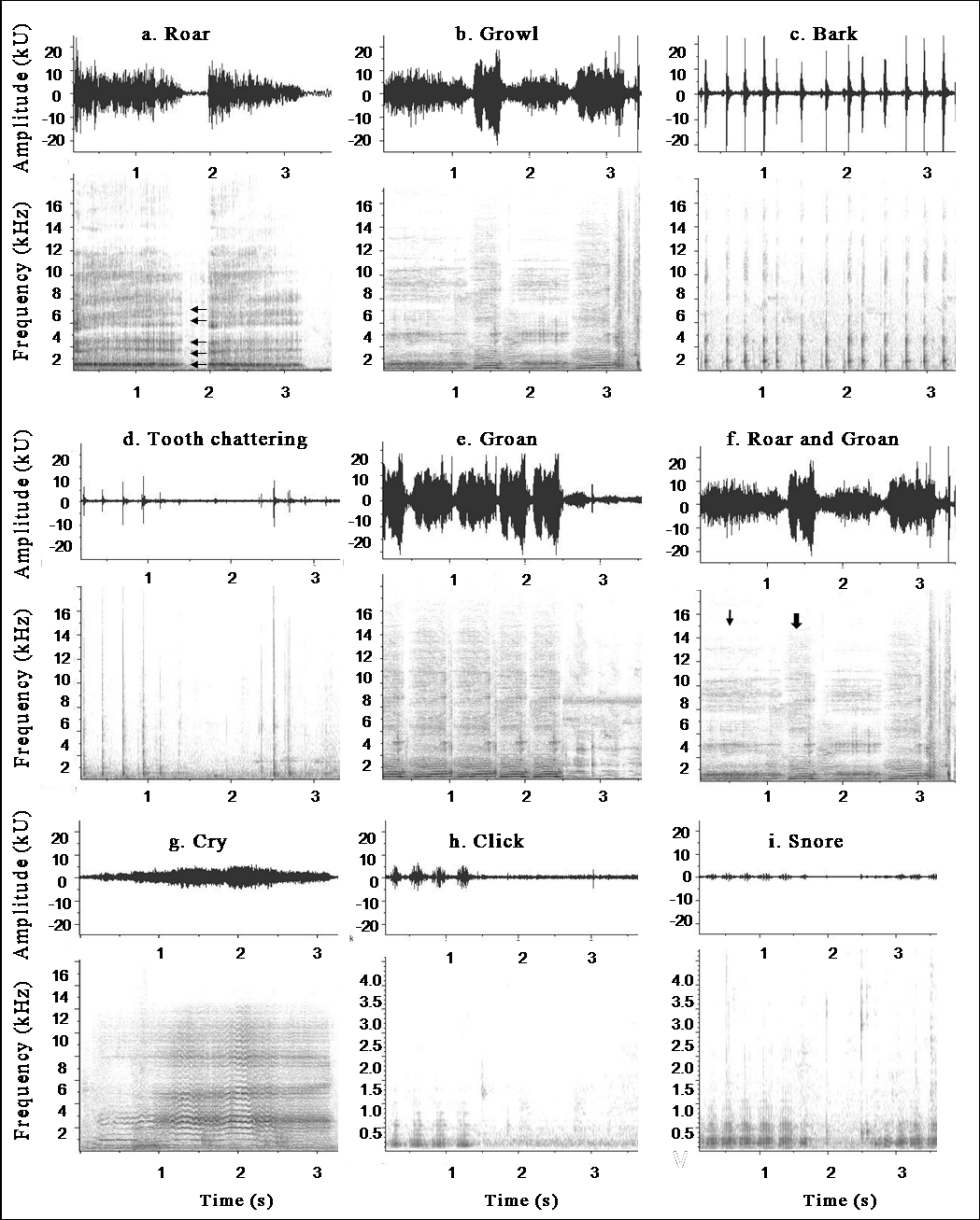
Spectrograms and time series of spotted paca vocalizations. The arrows in the first *roar* vocalization (box *a*) indicate five formants. The box *f* shows combinations of sounds between *roar* and *groan* calls, and the arrows (1^st^ and 2^nd^) of this box indicate the *groan and roar* calls, respectively.

Eight putative calls were found based on observations of the animals and visual inspection of the spectrograms (Fig 1; S4 Table). The hourly rate of emissions showed that *roar* (3.3 calls/s) was the most frequent vocal type, followed by *snore* (3.2 calls/s), *growl* (2.6 calls/s), *bark* (1.8 calls/s), *cry* (0.7 calls/s), *tooth chattering* (0.9 calls/s), *groan* (0.7 calls/s) and *click* (0.4 calls/s).

The LDA, based on the four acoustic parameters measured, discriminated the eight call types initially proposed (*roar*, *growl*, *bark*, *tooth chattering*, *groan*, *snore*, *click*, and *cry*) (MANOVA: *Wilks* λ = 0.10, *F*_28, 1404_ = 44.87; *P* < 0.0001, N = 400; Table 1). The first two discriminant functions explained 88.9% of variance among the vocalizations. The duration and dominant frequency were the variables that most contributed to the first discriminant function, whereas the minimum frequency was the variable that most contributed to the second function (Table 1). The cross-validation correctly attributed the vocal categories with an accuracy of 66%, which was significantly higher than the 12.5% (or 1/8) expected by chance (binomial test: *P* < 0.0001). The accuracy of cross-validation for the eight vocal categories ranged from 34% (*snore*) to 92% (*tooth chattering*) (Table 1). The spacing of different call types, denoted by the obtained centroids (Table 1) allows confirmation that they are well discriminated based on the factor axes extracted from the original explanatory variables.

The GLM confirmed differences between call types (F_20, 122_ = 6.3, *P* < 0.0001). This analysis also showed differences in the acoustic parameters of juveniles and adults (F_4, 46_ = 18.9, *P* < 0.0001). However, the statistical model showed no interaction between call types and age class (F_20, 122_ = 0.87, *P* = 0.56). Therefore, there were no differences in the acoustic parameters of call types (*growl*, *groan*, *roar*, and *bark*) shared by juveniles and adults. As just one juvenile emitted *tooth chattering* we excluded this call type from the GLM.

Besides the *bark* and *groan* alarm calls, *clicks* and *cries* were the only non-agonistic calls in the species’ repertoire. *Clicks* were emitted during the feeding context, probably as a contact call (Table 2). During mother-offspring separation, only the *cry* call (Fig 1) was emitted by offspring, suggesting a contact function (Table 2). During *cry* emissions by the offspring the mother remained agitated, moving back and forth in the maternity pen, sniffing the air and the wooden barrier between them. The mother could also produce *tooth chattering* and *bark*s in response to the offspring *cry* call. On one occasion, we observed one of the mothers performing thumping displays (when the animal beats the ground with its hind legs) during this separation period.

**Table 2:** Description of context and possible communicative function of the vocalizations of spotted paca associated with age (A: adult and J: juveniles) and sex (M: male and F: female).

Call	Communicative Function	Age and sex category	Context
Roar	Agonistic/Alarm	A, J, M, F	Loud and harsh sound emitted as a single element or in sequences of two to five elements. During these emissions, the animals were very close to one another, and usually adopted alert posture, exhibiting pilo-erection and open mouth. Recorded during agonistic encounters mainly by defense of burrows or food and during handling. In this last context, the roar seems to be an alarm call to a conspecific. Roar call was produced once by one female during copula avoidance. This vocalization seemed to repel conspecifics or keep them away from the vocalizing animals.
Snore	Agonistic	A, M, F	Low vocalization emitted in sequences of three to seven elements. They were produced when an animal approached burrows or food of another animal or a human being during pen cleaning. The animal assumed alert posture with minimal movements of mouth.
Tooth chattering	Agonistic	A, J, M, F	A mechanical signal produced by the clash of upper and lower incisors. This sound is produced in sequences of two to six elements. The animals produced the sound during defense of burrows, during mother/pup isolation and during human presence.
Bark	Alarm	A, J, M, F	Barks are produced alone or in sequences of two to ten short elements. This vocalization was emitted only when an animal was captured for medical procedures or during environmental disturbances (loud noises or presence of unfamiliar humans). Animals assumed alert posture and presented pilo-erection while vocalizing. Sometimes the animal jumped, trying to escape from the situation. A behavioral response from a conspecific, immediately after the call was released, could lead to freezing and hiding in burrows.
Growl	Agonistic	A, J, M, F	Growls are harsh sounds produced as a single element or in sequences of two to five elements. Growls were recorded in agonistic encounters mainly during defense of burrow or resources addressed only to conspecific.
Groan	Agonistic/alarm	A, J, M	Noisy vocalization with three to eight harmonic structures. These calls were emitted single or in a sequence of two to three elements. Produced when an animal was injured during agonistic encounters and seemed to express pain. When a dispute for food or burrows was lost or when animals were captured for handling, they emitted *groan* combined with *roars* that seemed to alert conspecific.
Click	Contact	A, M, F	Clicks are very low vocalizations emitted in sequences of three to eight elements This vocalization was produced just before feeding and when the keeper approached for food delivery.
Cry	Contact	J, M, F	Loud sound with broadband noise that presents harmonic structures emitted singly or in a sequence of two to four elements. Produced only by juveniles during mother-pup separation. The emission of this vocalization seemed to reestablish contact with mother.

**Fig 2:**
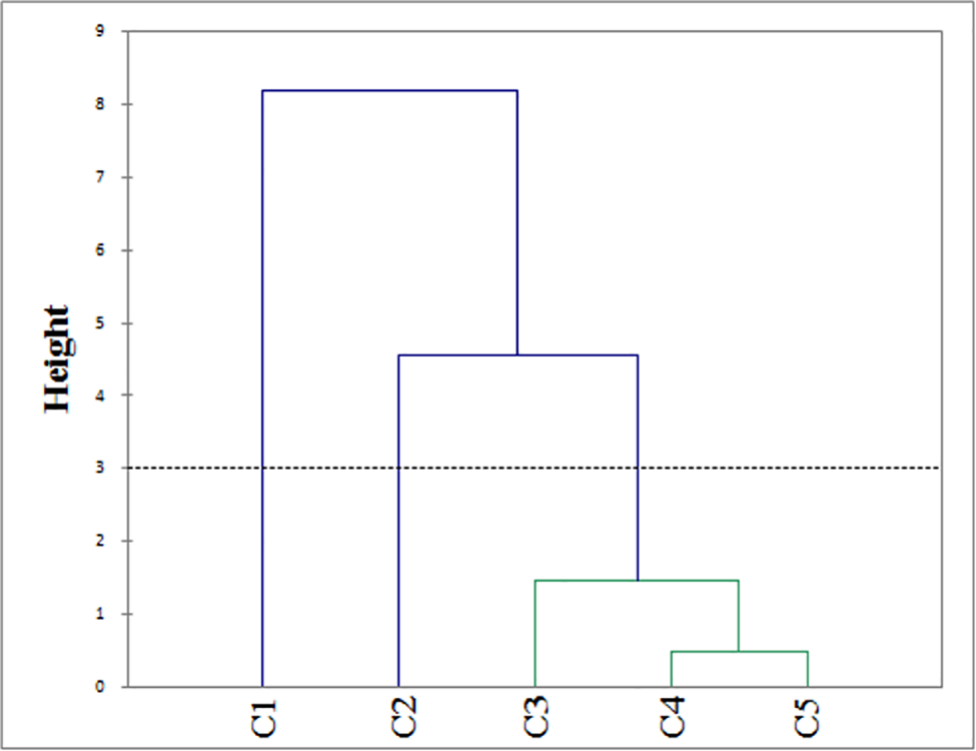
**Dendrogram of similarity according to acoustic parameters of five different combinations (C1-C5) of *roar* (A) and *groan* (B)**. C1 (BA), C2 (ABABAB), C3 (ABA), C4 (ABAB), C5 (ABABA). The letters A and B correspond to the sequences of roar (A) and groan (B) appeared in each combination. Hierarchical clustering with Ward’s method was used to construct the dendrogram. Height represents cophenetic distance (dissimilarity) between the combinations. The dotted line represents the automatic truncation, leading to three combination calls of *roar* and *groan*.

The spotted paca emitted *groan* combined with *roar* when captured for handling, which apparently seemed to alert conspecifics (Table 2). Although we recorded 14 combinations of both calls, we analyzed just five of these combinations, as these were emitted more than three times from at least three animals. The automatic truncation showed in dendrogram (Fig 2), reveals that from the five analyzed combinations of *roar* (A) and *groan* (B) three of them (ABA, ABAB, and ABABA) were acoustically similar, composing one unique vocalization.

For four spotted pacas, for which both anatomical and acoustic data were available (Table 3), the predicted and measured formant dispersion were not significantly different (paired *t*-test: *t* = 1.33; N = 4; *P* = 0.27). Standard deviations of formant dispersion were quite stable in spotted paca, ranging from 2.5% to 10.4% of the formant dispersion (mean 7.8%). Thus, spotted paca closely approximated the uniform tube approximation.

**Table 3:** Descriptive data for anatomical and acoustic variables of adult spotted paca (N = 4) used in formant analysis.

	Mean	S.D.	Min	Max
Body mass (kg)	4.6	0.7	3.8	5.5
Body length (cm)	50.1	2.5	47.0	53.0
Skull length (cm)	16.1	0.4	15.5	16.5
*VTL* (cm)	11.0	0.2	10.8	11.2
*D*_f_ (Hz)	1161	91	1099	1297

S.D.: standard deviation; Min and Max: minimum and maximum values; *VTL*: vocal tract length; *D*_f_: formant dispersion.

### Vocal complexity and group size: Phylogenetically based analysis

**Fig 3:**
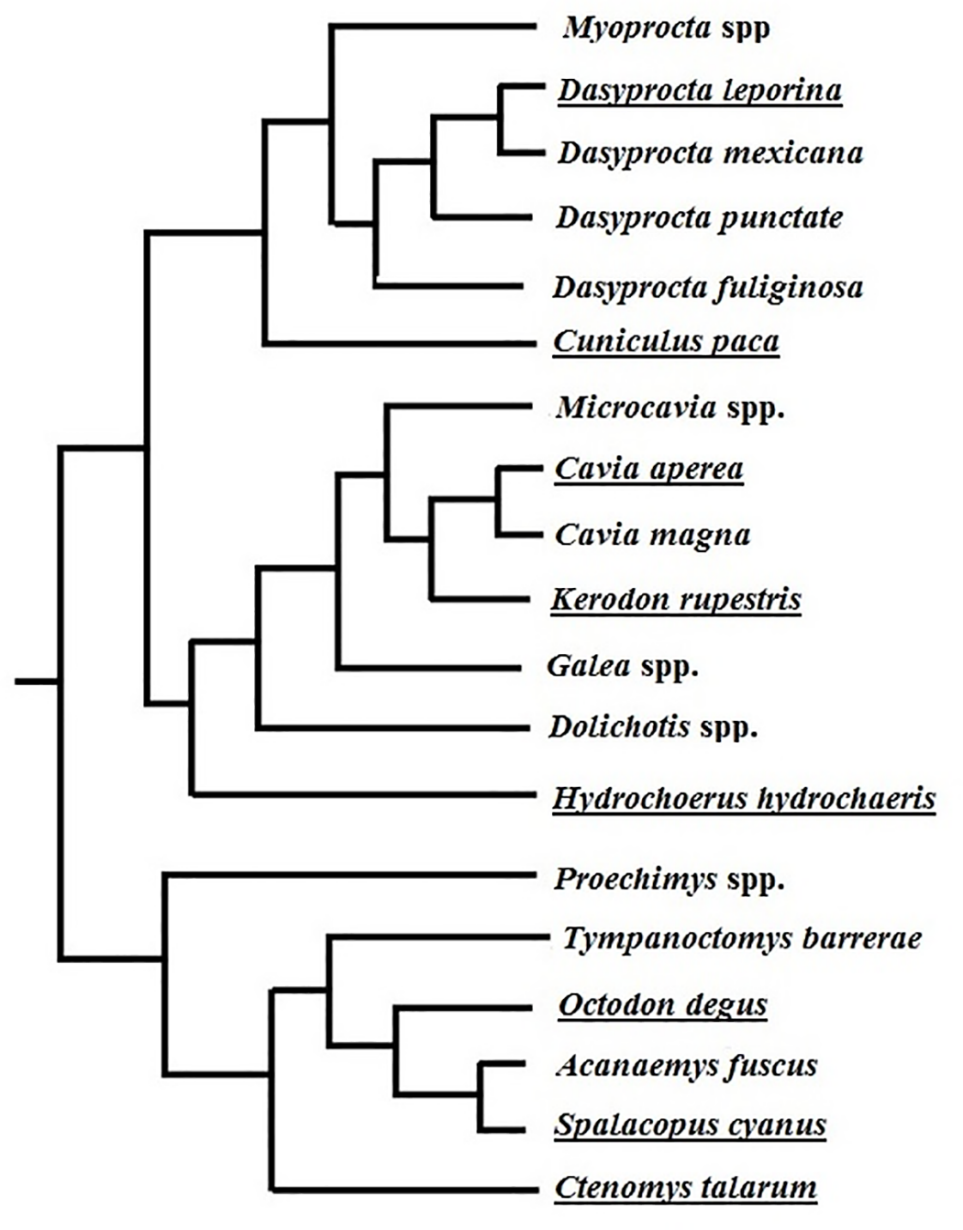
Topology of the species of caviomorph rodents used in this study. Modified from [5] and [53].

**Fig 4:**
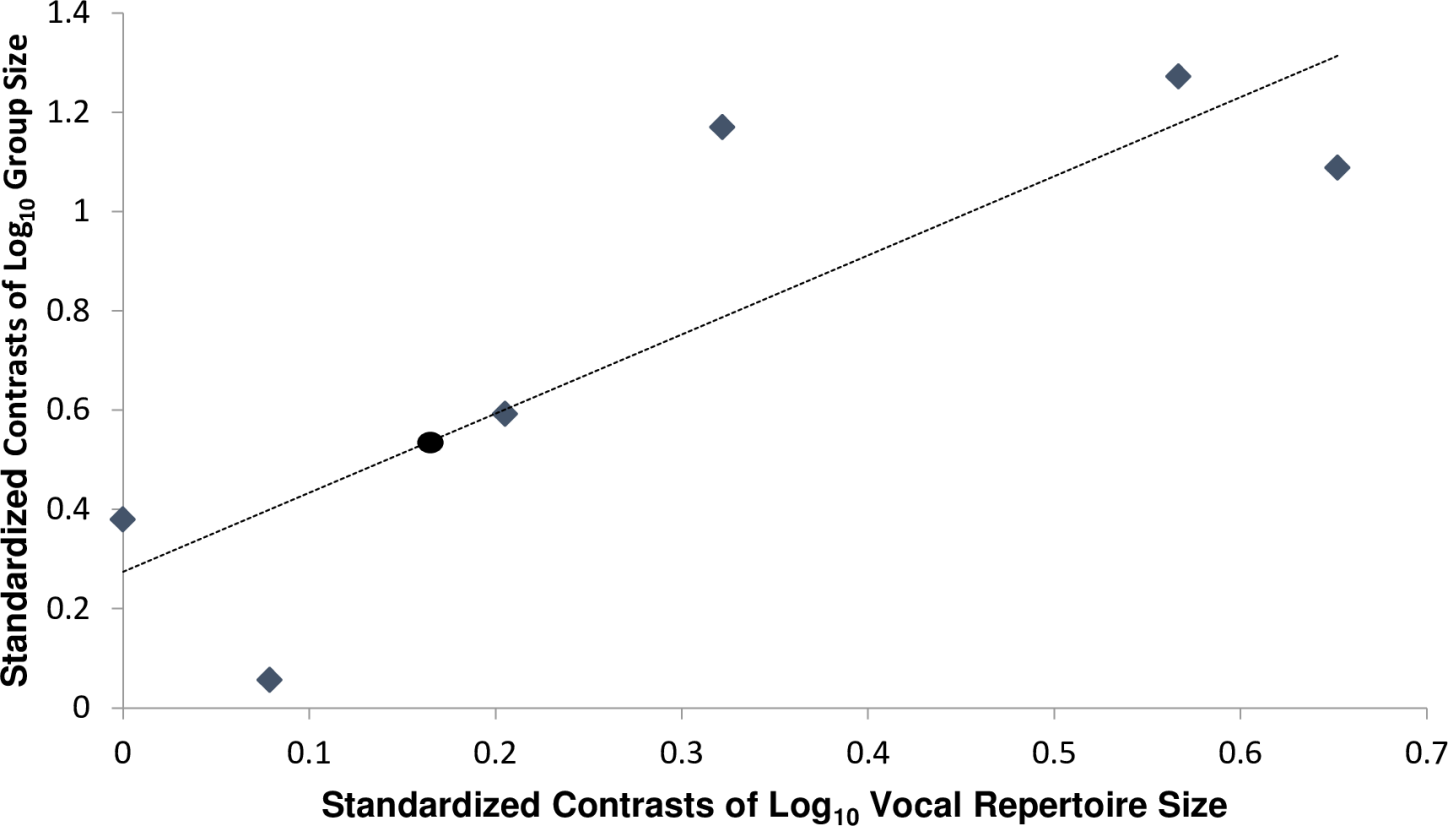
The relationship between standardized independent contrasts of log_10_ vocal repertoire size and standardized contrasts of log_10_ group size of seven caviomorph rodent species. The linear regression follows the equation: Standardized contrasts of log_10_ group size = 0.27 + 1.59*Standardized contrasts of log_10_ vocal repertoire size (F_1, 4_ = 10.41; R^2^ = 0.72; *P* = 0.03). Gray lozenge indicates the independent contrasts of the original seven caviomorph species, while black circle is the virtual position of the spotted paca based on the equation considering only its adult acoustic repertoire size (N = 6) determined in this study.

Using the available information on average group size and adult vocalizations repertoire size based on acoustic parameter information from seven species of caviomorph rodents (Table 4), as well as the phylogenies of these same species (Fig 3), we determined the relationship between group size and vocal repertoire size across independent contrasts analyses through the equation: Y = 0.27 + 1.59*X (F_1, 4_ = 10.41; R^2^ = 0.72; *P* = 0.03; Fig 4).

**Table 4:** Data of the seven caviomorph species used to analyze the relationship between group size and the number of vocal types.

Species	Average group size	References	Adult vocal repertoire* (number of types)	References
*Ctenomys talarum*	1	[74]	4	[70]
*Dasyprocta leporina*	2	[75]	8	[72, S1 Text]
*Cavia aperea*	2	[76]	8	[68]
*Octodon degus*	4	[77]	7	[67]
*Kerodon rupestris*	4.5	[78]	10	[69]
*Spalacopus cyanus*	8.5	[79]	11	[73]
*Hydrochoerus hydrochaeris*	9.5	[80]	6	[44]

* Excluding juveniles and mechanical vocal types.

Considering the acoustic repertoire size of adult spotted paca determined in this study to comprise six different acoustic vocalizations (Table 2)–excluding the mechanical call and juvenile vocalization–as well as the spotted paca’s position in the phylogeny (Fig 3) we calculated as 0.17 the standardized contrasts of log_10_ of vocal repertoire size of spotted paca. Then, replacing X by 0.17 (X = 0.17) in the obtained equation (Y = 0.27 + 1.59*X), we calculated as 0.55 the standardized contrasts of log_10_ group size of spotted paca (Y = 0.55). Using this value and the species’ phylogeny position we calculated the log_10_ group size of spotted paca as 0.94. Then, using inverse logarithm (anti logarithm), we estimated an average potential group-living size of 8.7 individuals for this species. If we consider the 95% confidence interval the potential group size of the spotted paca is estimated to be 6.0–12.7 individuals.

## Discussion

We provide evidence of the complexity in the vocal repertoire of spotted paca in numbers and features, such as combination of vocal types, which may increase the species’ acoustic repertoire even more. Our results also revealed the expected relationship between caviomorph rodents’ vocal repertoire size and group size (sociality). This relationship corroborates the hypothesis that the acoustic repertoire, together with other ecological factors [55], can be a predictor of sociality in caviomorph rodents. The complexity of spotted paca’s acoustic repertoire described herein, grants this species behavioral capabilities to be included within the more social caviomorph rodents.

The acoustic repertoire of captive spotted paca described here is larger than that previously recorded for free-ranging animals [37], which included six types of calls for the species. In the present study, based on LDA analysis, we confirmed the discrimination of eight acoustic signals from the eight calls initially proposed based on visual inspection of the spectrograms: seven vocal and one non-vocal call types. In an earlier study, Eisenberg [37] did not show the acoustic structure of the calls, which prevented the accurate description of these calls and the analysis of complexity in the repertoire of this species.

The independent contrasts analysis revealed that evolutionary changes in vocal repertoire are a predictor of changes in group-living. Through the linear regression analysis, we verified that the spotted paca has the potential to live in groups of between six and 13 individuals. The relationship between group-living and vocal complexity was also reported for other taxa. For instance, in non-human primates, increases in group size and social grooming, are related to evolutionary changes in acoustic repertoire size [24]. In whales, group sizes are related to differences in acoustic parameters [25], while individuals of Carolina chickadees (*Poecile carolinensis*) living in larger groups emitted calls with greater complexity than individuals in smaller groups [56]. It is not possible, however, to evaluate if vocal complexity (repertoire size) precedes or follows the increases in sociality (group size) during evolutionary processes [24]. In this context, the vocal complexity in spotted paca explains different sightings of groups ranging from one to five individuals in Peru, Mexico, Colombia and Brazil [33–35, 57] and may also explain the success of breeding groups in captivity [36], showing acoustic capabilities to manage social interactions among companions. Moreover, Nogueira-Filho and Nogueira [34] suggested that the solitary habit reported for the species might be a response to the intensity of hunting pressure, which is corroborated by the report that spotted paca changes its burrowing habits depending on hunting pressure [35].

Spotted paca provides the most sought-after game meat in Neotropical countries and suffers high hunting pressure, threatening the species [58–60]. This pressure could change the species’ behavior from more social to solitary as a strategy to escape from predation. Although the predatory risk hypothesis predicts that group-living reduces the risk of predation [4, 8], this behavioral pattern seems not to be adaptive for spotted paca, which use burrows to hide from predators [35]. Moreover, there is a correlation between burrow digging and sociality in caviomorphs [5, 8]. Therefore, burrowing behavior could also be an evolutionary trait reinforcing sociality capabilities in this species besides its vocal complexity. Furthermore, studies have showed that prey individuals can change their social strategies according to predation risk [61]. For instance, the African striped mouse (*Rhabdomys pumilio*) can adjust its social behavior in response to prevailing environmental conditions, in which both sexes switch between solitary- or group-living according to social tactics, to have a greater fitness at a particular time [62].

The occurrence of sociality is expected when the benefits are stronger than costs [7, 63]. This explains the variability recorded in the sociality degrees among and within species as current ecological factors may influence group composition, making it more or less cohesive [15]. Thus, the human-induced rapid environmental change (HIREC) *sensu* [2] could lead to solitary habits in the spotted paca instead of finding this species living in groups. Complexity features such as vocal combinations can change behavioral responses of vocal receivers. We found at least three different possible types of combinations of the *groan* and *roar* calls that were emitted in different contexts than those in which each call was emitted separately. In *roar* vocalizations we found formant-like structures. Formant structures, which reflect details of individual vocal-tract anatomy and body size [6], usually play an important role in individual recognition. Therefore, the formants may allow receivers to assess the caller’s age, sex and maturity [64, 65, 66], which could help, for instance, in the recognition of the reproductive condition of male/female in the spotted paca. However, further study using playback is still necessary to test the function of vocal combinations and formants in this species.

The vocal repertoire of spotted paca herein described, provides some reflection regarding the presence of homologies among caviomorph rodents. The *cry* call, for example, is structurally similar and seems to occur in the same behavioral context (contact/isolation) as the degu’s (*Octodon degus*) *loud whistle* [67] and the guinea pig’s (*Cavia porcellus*) *isolation whistle* [68]. The emission of *tooth chattering*, a mechanical signal described for spotted paca, was also present in several caviomorph species, including *Hydrochoerus hydrochaeris* [44], *Cavia* sp. [68], *Kerodon rupestris* [69], *Ctenomys talarum* [70], and showing similarities in behavioral contexts and acoustic structures [37]. The *groan* vocalization in the spotted paca is functionally like *groans* in degu [67] and *whine* in rock cavy (*Kerodon rupestris*) [69] and guinea pigs [68], all emitted in aggressive contexts. *Growls* emitted by spotted paca are agonistic calls and are possibly used as defensive function of the *grunt* call in tuco-tuco (*Ctenomys talarum*) [70], rock cavy [69], and degu [67], which provide similar acoustic patterns. *Bark* calls of spotted paca also show similarities with the capybara’s *bark* [44] in both function (alarm call) and acoustic features. *Click* and *snore* calls are acoustically like those of capybara and emitted during contact and agonistic contexts, respectively [44]. Nevertheless, based on our acoustic analyses, the *roar* vocalization emitted by spotted paca seems to be unique for this species. These similarities in function and structures, in the acoustic repertoire of caviomorphs, highlight phylogenetic relationships among taxa and urge more studies to use acoustic behavior as characters to rebuild phylogenies and acquire insights into the evolution of sociality in caviomorphs.

The repertoire complexity described here corroborates the hypotheses of sociality for the spotted paca. Our results also support vocal complexity as a predictor for sociality in caviomorph rodents. Nevertheless, these findings show that measurements of complexity in both social systems and communication systems are challenging and liable to arbitrary decisions, depending on how these behavioral features are considered and which metrics are used as highlighted by Fischer et al. [71]. Moreover, we could not include data from wild spotted paca due to the species’ nocturnal habit and defensive behavior. Therefore, we must consider that our data is not a definitive description of the spotted paca’s repertoire, because contexts and motivational states could be restricted in the captive environment. Nonetheless, the data here described urges researchers to continue investigating the species’ vocal repertoire in more diverse contexts, to test the function of the vocalizations already described and to apply the knowledge about vocal communication to the investigation of evolutionary and ecologically relevant questions.

## Supporting information

S1 Table Dataset of acoustic parameters of spotted paca’s vocalizations. (XLS)

S2 Table Dataset of combination of *roar* and *groan* vocalizations. (XLSX)

S3 Table Dataset of formants in *roar* vocalizations. (XLSX)

S4 Table Dataset of acoustic types of spotted paca. (RAR)

S1 Text Master Thesis Lima SGC. (PDF)
