# Supplementary material for: Vocal complexity and sociality in spotted paca (Cuniculus paca)
Source: PLoS One. 2018 Jan 24;13(1):e0190961. doi: 10.1371/journal.pone.0190961 (PMC5783385; doi:10.1371/journal.pone.0190961)
Supplement: S1 Text — (PDF) [file pone.0190961.s005.pdf]

UNIVERSIDADE FEDERAL DO RIO GRANDE DO NORTE

CENTRO DE BIOCIÊNCIAS

PROGRAMA DE PÓS-GRADUAÇÃO EM PSICOBIOLOGIA

STELLA GUEDES CALAZANS LIMA

COMPORTAMENTO ACÚSTICO E COMPLEXIDADE SOCIAL EM CAVIOIDEA

NATAL-RN

2016

STELLA GUEDES CALAZANS LIMA

COMPORTAMENTO ACÚSTICO E COMPLEXIDADE SOCIAL EM CAVIOIDEA

Tese apresentada ao Programa de Pós-Graduação em Psicobiologia da Universidade Federal do Rio Grande do Norte como parte dos requisitos obrigatórios para obtenção do título de Doutora em Psicobiologia.

Orientadora: Profa. Dra. Renata Santoro de Sousa Lima Mobley

Co-orientadora: Profa. Dra. Selene Siqueira da Cunha Nogueira

NATAL – RN

2016

Universidade Federal do Rio Grande do Norte – UFRN
Sistema de Bibliotecas – SISBI
Catalogação da Publicação na Fonte - Biblioteca Central Zila Mamede

Lima, Stella Guedes Calazans.
Comportamento acústico e complexidade social em cavioidea / Stella Guedes Calazans Lima. - 2016.
131f.: il.

Tese (doutorado) - Universidade Federal do Rio Grande do Norte, Centro de Biociências, Programa de Pós-Graduação em Psicobiologia.Natal, RN, 2016.
Orientador: Prof.ª Dr.ª Renata Santoro de Sousa Lima Mobley.
Coorientador: Prof.ª Dr.ª Selene Siqueira da Cunha Nogueira.

1. Bioacústica - Tese. 2. Caviomorfo- Tese. 3. Complexidade vocal- Tese. 4. Comunicação- Tese. 5. Histricognata- Tese. I. Mobley, Renata Santoro de Sousa Lima. II. Nogueira, Selene Siqueira da Cunha. III. Título.

RN/UF/BCZM   CDU 551.463.2

Título: Comportamento Acústico e Complexidade Social em Cavioidea

Autor: Stella Guedes Calazans Lima

Data da defesa: 22/11/2016

Banca examinadora

Prof Dr Rogério Grasseto Teixeira da Cunha

Universidade Federal de Alfenas

Prof Dr Carlos Barros de Araújo

Universidade Federal da Paraíba

Profª Drª Maria Bernadete Cordeiro de Sousa

Universidade Federal do Rio Grande do Norte

Prof Dr Arrilton Araújo de Souza

Universidade Federal do Rio Grande do Norte

Profª Drª Renata Santoro de Sousa Lima Mobley

Universidade Federal do Rio Grande do Norte

Dedico aos meus eternos amores,
Daniel, Jonas e minha familia que me apoiaram e incentivaram durante essa caminhada de grande crescimento pessoal e profissional.

AGRADECIMENTOS

Ao meu amor maior, meu filho Daniel, por iluminar a minha existência e me alegrar em todos os momentos da minha vida.

Ao meu eterno amor Jonas, grande incentivador que mesmo não estando mais entre nós, continua vivo nas minhas lembranças e no meu coração e do nosso filho.

Aos meus familiares, em especial a minha mãe Suely que cuidou com amor do meu filho para que eu pudesse me dedicar aos estudos. Ao meu pai Reinaldo e aos meus irmãos Felipe, Alice e Renata pelo constante incentivo, companheirismo, amor e carinho dedicados a mim.

A Roberto Magalhães pela paciência, ajuda e companheirismo dedicados a mim.

A Prof.ª Dr.ª Selene Nogueira, pela excelente orientação e por ser um exemplo de pesquisadora e professora, agindo sempre com ética e seriedade, sem a qual, seria improvável a existência deste trabalho.

A Prof.ª Dr.ª Renata Sousa Lima, pela orientação e importante papel para a realização deste trabalho.

A profª Suemi Tokumaro e Christini Caselli pela colaboração durante este trabalho.

Aos professores Drª Elisabeth Spinelli de Oliveira, Dr Carlos Barros de Araújo, Drª Maria Bernadete Cordeiro de Sousa e Dr Arrilton Araújo de Souza por terem aceitado fazer parte dessa banca de doutorado.

A todos os meus amigos e parentes que sempre me motivaram e entenderam as minhas ausências durante essa longa caminhada de estudos.

Ao CNPq pela concessão da bolsa de estudos.

A UFRN e ao programa de pós-graduação em Psicobiologia, pela oportunidade de estudo e pesquisa.

COMPORTAMENTO ACÚSTICO E COMPLEXIDADE SOCIAL EM CAVIOIDEA

RESUMO

A relação entre comunicação acústica e socialidade tem sido o foco de vários estudos sobre a hipótese da complexidade social. Essa hipótese afirma que animais que vivem em grupos sociais mais complexos necessitam de um repertório mais diversificado para produzirem maior quantidade de informações. No entanto, ainda faltam estudos que melhor expliquem qual a direcionalidade causal entre a complexidade social e vocal em mamíferos, além de quais fatores ambientais e/ou sociais podem ter co-evoluído e contribuído para a complexidade na comunicação de mamíferos. Nesse contexto, o estudo comparado entre espécies de caviomorfos que diferem em seus sistemas sociais e ecológicos, pode contribuir para este entendimento. O grupo dos caviomorfos é composto por uma ampla diversidade de espécies, que apresentam variedade no tamanho e composição dos grupos sociais, nos tipos de sistemas de acasalamento, nos tipos de sistemas sociais, nos tipos de hábitat e nos repertórios acústicos. Essas características do táxon estão disponíveis na literatura para 10 espécies de caviomorfos. Nesse estudo, objetiva-se ampliar o conhecimento sobre os repertórios acústicos de *Cuniculus paca* e *Dasyprocta leporina*, além de explorar a relação entre complexidade social e vocal nos caviomorfos. Dessa forma, o capítulo um desta tese, descreve o repertório vocal de pacas mantidas em cativeiro identificando-se sete tipos de vocalizações emitidas principalmente em contextos agonísticos, possivelmente relacionados com sua defesa territorial. Encontramos uma complexidade vocal que não era esperada em uma espécie solitária como a paca. O capítulo dois descreve o repertório acústico da cotia vermelha, composto por 10 tipos de chamados associados principalmente a contextos agonísticos e de defesa durante o período de alimentação. Foram confirmadas diferenças sexuais nos chamados *có, gemido, rangido 7, gru, latido* e *rosnado*, os quais geralmente apresentam freqüências mais graves para os machos. Ainda foram encontradas gradações, transições e combinações de sons que adicionam complexidade ao repertório acústico de cotias vermelhas, como esperado para espécies que vivem em pares ou formam pequenos grupos familiares. O terceiro capítulo apresenta dados sobre a correlação entre complexidade social e vocal entre dez espécies de caviomorfos. Os resultados confirmam que quanto mais complexo o tipo de sistema social da espécie (composição do grupo, tamanho da prole, sistema social e sistema de acasalamento) maior seu repertório vocal amigável.

Palavras chave: Caviomorfo, Histricognata, Bioacústica, Complexidade vocal, Comunicação

ACOUSTIC BEHAVIOR AND SOCIAL COMPLEXITY IN CAVIOIDEA

ABSTRACT

The relationship between acoustic communication and sociality has been the focus of several studies concerning the hypothesis of social complexity for communication. This hypothesis state that animals that live in complex social groups, requires a more diverse repertoire to produce more information. However, there is a lack of studies that explain the causal directionality between social and vocal complexity in mammals, and that identify which factors -environmental and/or social - may have co-evolved and contributed to the complexity of mammalian communication. In this context, the comparative study between caviomorph species that differ in their social and ecological systems, may contribute to the understanding of this system. The caviomorph rodents are comprised of a wide diversity of species, which present variation in the size and composition of social groups, in the types of mating systems, in the types of social systems, in the types of habitat and size of acoustic repertoires. These characteristics of this taxon are available in the literature for ten species of caviomorph. In this study, we aim to provide data about the acoustic repertoire of *Cuniculus Paca* and *Dasyprocta leporina* species as well as explore the relationship between social and vocal complexity in caviomorphs. Thus, the first chapter of this work describes the vocal repertoire of spotted pacas kept in captivity. The results revealed seven types of vocalizations, mainly emitted in agonistic contexts, possibly related to territorial defense. We also found an unexpected degree of vocal complexity for this solitary species. The second chapter describes the acoustic repertoire of red-rumped agoutis, consisting of 10 types of calls, mainly associated with agonistic and defense contexts during the feeding period. Sexual differences were identified in several vocalizations: "*coo", "groan", "creak-squeak", "grunt", bark and* "*snarl"*, which generally have lower frequencies for males. We also found gradations, transitions and combinations of sounds that add complexity to the acoustic repertoire of red-rumped agouti, as expected for species that live in pairs or form small family groups. The third chapter tests the correlation between social and vocal complexity among ten species of caviomorphs. The results confirmed that there is a positive relationship between the complexity in the social system of the species (group composition, size of offspring, social system and mating system) and the size of caviomorph friendly vocal repertoires.

Keywords: Caviomorph, Hystricognathi, Bioacoustic, Vocal Complexity, Communication

LISTA DE FIGURAS

1. Introdução Geral

1 Taxonomia de Caviomorpha e as relações filogenéticas das superfamílias Erethizontidae, Chinchiloidea, Octodontoidea e Cavioidea, segundo revisão de Woods e kilpatrick (2005)...20

2 Esquema das relações filogenéticas da família Caviidae, segundo Woods e Kilpatrick (2005). Kerodon que fazia parte de Caviinae (destacado em cinza), agora forma com Hydrochoerus a subfamília Hydrochoerinae...21

2. Capítulo 1: The Does the acoustic repertoire of spotted paca (*Cuniculus paca*) can explain the species sociality?

1 Spectrograms and time series of spotted paca vocalizations. The arrows in boxes *a* and *b* indicate formants, and in box *d* indicate a sudden transition to chaotic dynamics of sound production, resulting in deterministic chaos. Combination of sounds between roar and groan calls in box *f*, arrow indicate groan call. Gradation between growl-bark (box *b* and *c* respectively) and click-snore (box *g* and *h* respectively) results from similarity in the acoustic structure between these calls…………..41

3. Capítulo 2: Repertório Acústico da cotia vermelha (*Dasyprocta leporina*)

1 Espectrogramas e respectivos oscilogramas das vocalizações emitidas por cotias ………………………..…...…65

2 A estrutura acústica de vocalizações de cotias. Espectrogramas e oscilogramas mostram freqüência e amplitude sobre o tempo. Símbolos: *Rangido longo e ** rangido curto, respectivamente.…..66

3 Espectrogramas e respectivos oscilogramas de vocalizações emitidas por cotias..…..67

4. Capítulo 3: Complexidade Vocal e Social em Caviomorfos

1 Esquema de contagem de notas múltiplas ou combinadas utilizadas neste trabalho. Número de notas contabilizadas entre parênteses………...................................….......…93

2 Correlação entre as variáveis vocais, sociais e ecológicas nos dois primeiros componentes principais (CP1 e CP2)..…...102

3 Regressão linear entre tamanho do repertório acústico e complexidade do tipo de sistema social seguindo a equação y = 1,58x + 2,29 ($F_{1, 8}$ = 19,65, R^2 = 0,71, P = 0,02)..............103

4 Relação entre o repertório amigável e as variáveis sociais: (A) composição do grupo de acordo com a equação: y = 1,67x - 1,43 ($F_{1, 8}$ = 21,39, R^2 = 0,73, P < 0,002). (B) Tamanho da prole de acordo com a equação: y = 0,69x - 1,05 ($F_{1, 8}$ = 11,00, R^2 = 0,58, P < 0,01). (C) Tipo de sistema social de acordo com a equação: y = 0,89x - 1,30 ($F_{1, 8}$ = 10,50, R^2 = 0,57, P < 0,01). (D) Sistema de acasalamento de acordo com a equação: y = 1,61x - 1,16 ($F_{1, 8}$ = 10,02, R^2 = 0,56, P < 0,01)...…...104

LISTA DE TABELAS

1. Capítulo 1: Does the acoustic repertoire of spotted paca (*Cuniculus paca*) can explain the species sociality?

1 Mean ± standard deviation of each acoustic parameter measured in spotted paca vocalizations. Coefficients of the two main discriminant functions (DF) indicating the relative contribution of each acoustic parameter (variable) for the distinction among vocal types and the percentage of notes correctly attributed to each call type in the cross validation. The N corresponds to the number of emissions analyzed in each category. The values in bold indicate the parameters with higher loading for the discriminant functions (DF1 and DF2)……………………………....……………………………………...…..…43

2 Description of context and possibly communicative function of the vocalizations of spotted paca associated with age (A: adult and J: juveniles) and sex (M: male and F: female)...........................……………………………………………..…………......…45

3 Comparison of acoustic parameters of the sequences formed by *clicks* and *snores*. The *P*-values were adjusted by FDR correction and significant differences are highlighted in bold. The N corresponds to the number of sequences analyzed in each category..47

2. Capítulo 2: Repertório Acústico da cotia vermelha (*Dasyprocta leporina*)

1 Número de indivíduos e composição sexual (Macho, Fêmea) de 13 grupos (A-M) de cotias vermelhas utilizadas nesse estudo……...….61

2 Coeficientes das duas principais funções discriminantes (DF) indicando a contribuição de cada parâmetro acústico para distinção entre os tipos vocais. Os valores em negrito

indicam os parâmetros com maior peso para a função discriminante (DF1 e DF2)...68

3 Média ± desvio padrão de cada parâmetro acústico medido das vocalizações de cotia vermelha e a porcentagem de notas atribuídas corretamente a cada tipo de chamado na validação cruzada. N corresponde ao número de emissões analisadas em cada categoria...70

4 Médias e valores de P dos parâmetros acústicos utilizados na Análise de variância (ANOVA). Efeito significativo destacado em negrito..72

5 Diferenças significativas entre tipos vocais de cotias vermelhas (testes post hoc de Tukey p<0.05). Os numeros correspondem a 1 –freq. min., 2 – freq. max., 3 – faixa de freq., 4 – duração, 5 – freq. fundamental, 6 – intervalo entre notas...73

6 Descrição do contexto comportamental e possível função comunicativa dos sons de cotias associadas a idade (A: adultos e J: jovens) e sexo (M: macho e F: fêmea) do emissor..75

3. Capítulo 3: Complexidade Vocal e Social em Caviomorfos

1 Tamanho dos repertórios e características sociais, reprodutivas, ecológicas e morfológicas de 10 espécies de roedores caviomorfos. Escores usados estão representados entre parêntesis...…96

2 Matriz dos componentes principais, autovalores, variância e variância acumulada entre as variáveis acústicas, sociais e ecológicas nos quatro primeiros componentes principais (CP 1, CP 2 CP 3 e CP 4). Os valores em negrito indicam as variáveis com maior peso na análise em cada componente..101

SUMÁRIO

1.INTRODUÇÃO GERAL………..….............…...16

1.1 A Complexidade Social conduz a complexidade de sistemas de comunicação ou vice-versa?...16

1.2 A ordem Rodentia e os roedores Caviomorfos...19

1.3 A paca (*Cuniculus [Agouti] paca*)...23

1.4 A cotia vermelha (*Dasyprocta leporina*)..25

1.5 Informações sobre repertórios vocais de espécies de roedores caviomorfos...27

1.6 Objetivos...31

1.7 Hipóteses...32

2. CAPÍTULO 1

Does the acoustic repertoire of spotted paca (*Cuniculus paca*) can explain the species sociality? …………………..…...33

Abstract……………………………..........………….................….…..33

Introduction………………………………..………...................……....…34

Material and Methods……………..……..…35

Results……………………..……………….…......................………………..…40

Discussion…………………………….….….…………...............................…...48

Acknowledgements ………………….………………………………......………..…52

Literature Cited…………………………......……………............………........…52

3. CAPÍTULO 2

Repertório Acústico da cotia vermelha (*Dasyprocta leporina*)............................….58

Resumo……......……………..…...……………..…..………..58

Introdução………………...………………………...…......…59

Material e Métodos…………………………....................................…………60

Resultados………………..................…………………..............................…………64

Discussão…………….......................…………………...…...78

Conclusão...82

Referências……………………......…........................……………….........................…83

4. CAPÍTULO 3

Complexidade vocal e social em caviomorfos……..........…………………………..87

Resumo……...........……………………………………..….....................…………..87

Introdução……………………........……………………...........................…......…88

Métodos……………………………….....................………………........…………92

Resultados……………………………………..….....……..........……...…100

Discussão……………………….....……………..….……………...................…105

Referências………………......................……………......……..…......………..........109

5. CONSIDERAÇÕES FINAIS...120

6. REFERÊNCIAS BIBLIOGRÁFICAS...121

1. INTRODUÇÃO GERAL

1.1 A Complexidade Social conduz à complexidade de sistemas de comunicação ou vice-versa?

A comunicação acústica em mamíferos é utilizada para mediar interações sociais entre coespecíficos em contextos variados (Blumstein e Armitage, 1997; Freeberg, 2006; Pollard e Blumstein, 2011; Freeberg et al., 2012). Espera-se, portanto, que haja alguma relação entre a comunicação acústica e socialidade dos animais (Francescoli, 1999; Schleich e Busch, 2002). Vários estudos têm obtido fortes evidências indicando que animais que vivem em grupos sociais mais complexos apresentam um repertório mais diverso para produzirem maior quantidade de informações (Blumstein e Armitage, 1997; McComb e Semple, 2005; Freeberg, 2006; Freeberg et al., 2012). No entanto, há ausência de estudos empíricos que melhor expliquem a direcionalidade causal da complexidade social, isso é, o que viria primeiro a complexidade vocal ou a complexidade social? (Ord et al., 2012; Krams et al., 2012).

A complexidade social pode ser mensurada através de atributos de socialidade (Pollard e Blumstein, 2012) como a estrutura social do grupo, número de papéis sociais (Blumstein e Armitage, 1997), a densidade do grupo, a força das ligações sociais (Silk et al., 2003), sistemas de acasalamento (Devillard et al., 2004), ou o tamanho do grupo social (McComb e Semple, 2005; Freeberg, 2006). Um atributo da complexidade social, que talvez seja o mais simples e o mais estudado em uma variedade de taxa, é o tamanho do grupo social (Pollard e Blumstein, 2012). Vários estudos apresentam evidencias da existência de correlação entre o tamanho do repertório vocal e o tamanho do grupo social (Blumstein e Armitage, 1997; McComb e Semple, 2005; Freeberg, 2006; Freeberg e

Harvey 2008; Freeberg et al., 2012). Esta relação pode ser explicada pela hipótese da complexidade social, segundo a qual, o número de indivíduos em um grupo social pode afetar diretamente a comunicação, uma vez que uma maior quantidade destes, gera a necessidade dos animais exibirem diferentes tipos de expressão comunicativa (Wilkinson, 2003). Considerando esta relação, quando os limites de um grupo social são bem definidos, apenas uma contagem simples dos indivíduos seria necessária para projetar tal perspectiva (Pollard e Blumstein, 2012). Dessa forma, o tamanho do grupo social pode influenciar a extensão e o potencial de informação do repertório acústico de uma espécie, aumentando assim sua complexidade (Freeberg et al., 2012).

A complexidade acústica pode ser medida por meio de atributos como o tamanho do repertório (maior número de tipos de sons) (Blumstein e Armitage, 1997; McComb e Semple, 2005), e a quantidade de *bits* de informação em um sistema de sinais acústicos (Freeberg, 2006; Krams et al., 2012). Sinais vocais podem ainda ser complexos por estarem associados a referenciais externos tais como chamados de alarme que comunicam um tipo de predador como ocorre em *Suricata suricatta (*Manser, 2001)*, por exemplo.* A presença de informações como idade, sexo ou identidade do emissor em assinaturas vocais também aumentam o repertório e a complexidade vocal (Pollard e Blumstein, 2011). Assim como uma maior proporção de vocalizações "amigáveis" em espécies mais sociais, em comparação com espécies solitárias, também refletem complexidade vocal (Leroux et al., 2009). Vocalizações amigáveis são descritas como chamados emitidos durante contextos de apaziguamento com função de reforçar a coesão do grupo (Morton, 1977). O uso de gradações, transições e combinações de sons percebidos nos repertórios de várias espécies de mamíferos (*Pan troglodytes*, Crockford e Boesch, 2005; *Aonyx cinerea*, Lemasson et al., 2014 *Pteronura brasiliensis,* Leuchtenberger et al., 2014), também podem aumentar a variabilidade acústica o que pode resultar em um repertório mais complexo

(Schassburger, 1993; Wilson, 2000). A hipótese da complexidade vocal (Freeberg et al. 2012) prediz que repertórios grandes e com gradações, transições e combinações de sons são esperados por ocorrer com maior frequência em espécies altamente sociais, quando comparadas a espécies solitárias (McComb e Semple, 2005; Freeberg et al., 2012).

A diversidade de espécies de roedores, com uma ampla variedade de sistemas sociais, de uso de habitat e de repertório acústico (Emmons e Feer, 1997; Wolff e Sherman, 2007), tem levado à escolha desse grupo como modelo para inúmeros estudos experimentais e observacionais em diversas áreas, incluindo ecologia, psicologia e comunicação animal (Eisenberg, 1974; Wolff e Sherman, 2007; Ebensperger e Hayes, 2016). As informações alojadas na ampla base de dados destes taxa podem ser úteis para testar hipóteses sobre a complexidade dos sistemas sociais (Wolff e Sherman, 2007; Ebensperger e Hayes, 2016). Dentre os roedores, os caviomorfos apresentam-se como um importante táxon para analisar a relação entre complexidade social e vocal por já existir uma grande disponibilidade de dados na literatura sobre sua ecologia, comportamento social e repertório acústico (Francescoli et al., 2016). No presente estudo, objetiva-se explorar a relação entre complexidade social e vocal nos caviomorfos, além de ampliar os dados da literatura no que se refere ao repertório acústico ainda pouco conhecido de duas espécies deste grupo (*Cuniculus paca* e *Dasyprocta leporina*). . Ocapítulo um desta tese dedica-se à descrição do repertório vocal de pacas (*Cuniculus* [*Agouti*] *paca*) mantidas em cativeiro e avalia este repertório com relação a seu tipo social, territorialidade e complexidade vocal e social. Este capítulo em particular, já foi escrito na língua inglesa em forma de manuscrito e pretende-se submetê-lo ao periódico científico *Behavioral Processes*. O capítulo dois tem como objetivo descrever o repertório acústico da cotia vermelha (*Dasyprocta leporina*), espécie caviomorfa da qual nada sobre sua comunicação acústica consta na literatura, até o momento. Este segundo capítulo que segue em formato

de manuscrito, será submetido ao periódico científico *Behavioural Processes*. O terceiro e último capítulo desta tese completa o raciocínio principal deste documento apresentando dados sobre a correlação entre complexidade social e vocal em dez espécies de caviomorfos, já incluindo os dados gerados pelas duas espécies aqui descritas. Este último capítulo submeteremos ao periódico *Animal Behaviour*.

1.2 A ordem Rodentia e os roedores Caviomorfos

Os roedores formam a ordem mais numerosa, com maior diversidade e distribuição geográfica, com mais de 2.000 espécies que abrangem aproximadamente 44% de todos os mamíferos (Simpson, 1974; Wilson e Reeder, 1993; Wolff e Sherman, 2007). A característica em comum que une essa ordem é um par de incisivos que são usados para defesa, trituração de alimentos e escavação de túneis (Wolf e Sherman, 2007). Os roedores podem ser classificados em duas subordens principais, Sciurognatas e Histricognatas (Tulberg, 1899). Os primeiros são caracterizados pelo ângulo da mandíbula inferior ter origem lateral ao incisivo (Tulberg, 1899). Já os histricognatas possuem o ângulo da mandíbula inferior originado abaixo do incisivo (Tulberg, 1899).

Os histricognatas representam um grupo monofilético (Luckett e Hartenberger, 1993; Nedbal et al., 1996; Huchon et al., 2000; Adkins et al., 2001; Huchon e Douzery, 2001) que contém 17 das 28 famílias de roedores existentes (Huchon et al., 2000; Adkins et al., 2001). Podem ser subdivididos em três grandes grupos: Bathy-Phiomorpha - que representa três famílias (Bathyergidae, Thryonomyidae e Petromuridae) endêmicas da África sub saariana (Wood, 1965), Hystricomorpha - consistindo de porcos-espinhos do velho mundo distribuídos na África e Ásia (Wood, 1965) e Caviomorpha - um grupo sul-americano contendo13 das 17 famílias de roedores (Lavocat, 1973). Os Caviomorpha são

subdivididos nas superfamílias Erethizontoidea, Chinchilloidea, Octodontoidea e Cavioidea (Patterson e Wood, 1982).

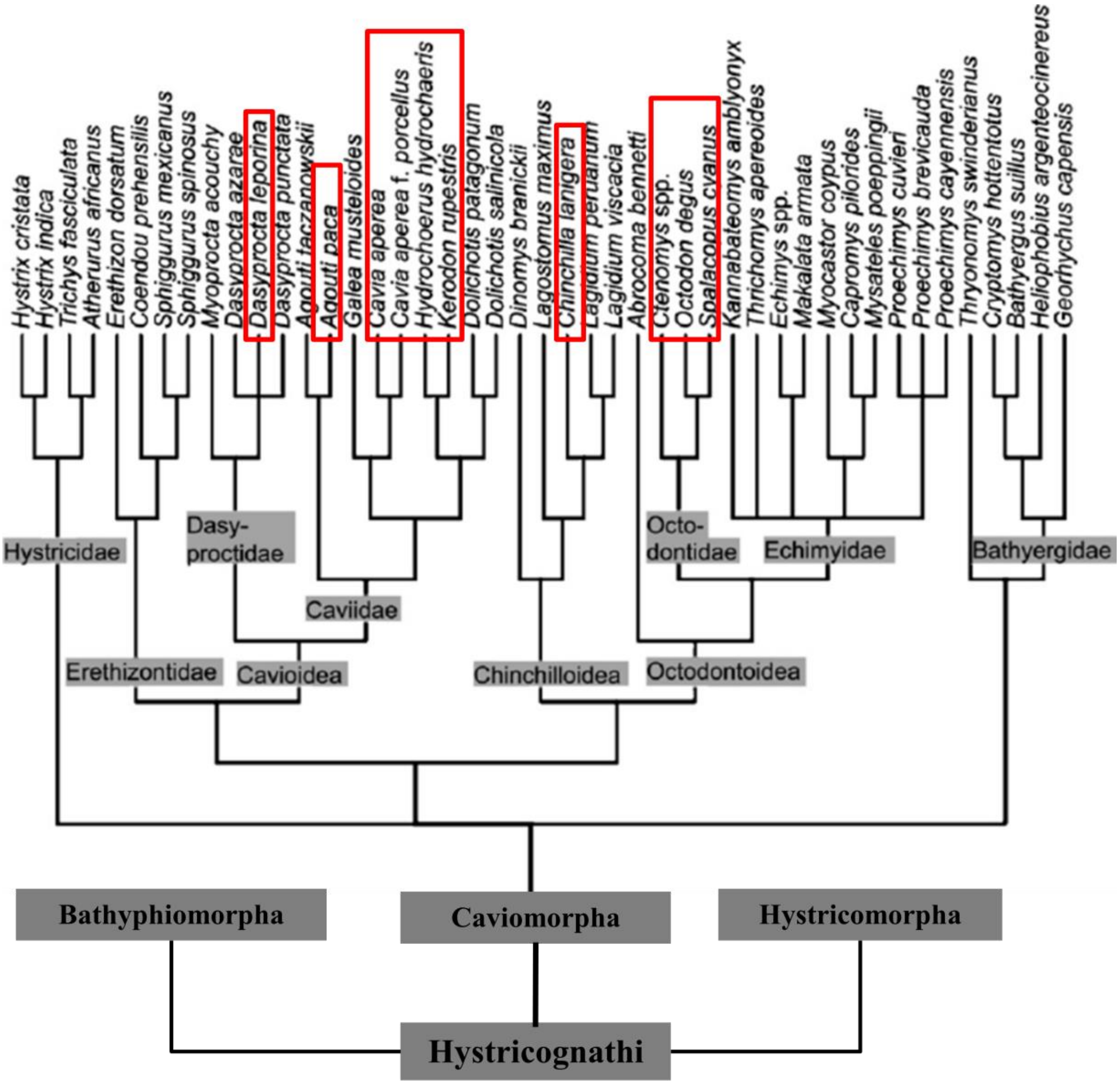

Fig. 1 Taxonomia de Caviomorpha e as relações filogenéticas das superfamílias Erethizontidae, Chinchiloidea, Octodontoidea e Cavioidea, segundo revisão de Woods e kilpatrick (2005). As espécies destacadas em vermelho foram utilizadas neste trabalho.

A superfamília Cavioidea é tradicionalmente dividida em três ou quatro famílias: Caviidae, Hydrochaeridae, Dasyproctidae (Corbet e Hill, 1991; Wilson e Reeder,1993), e Agoutidae (McKenna e Bell, 1997). A família Caviidae é dividida em duas subfamílias: Dolichotinae, que representa dois gêneros *Dolichotis* e *Pediolagus*, e Caviinae, com 4 gêneros *Kerodon, Galea, Cavia* e *Microcavia*.

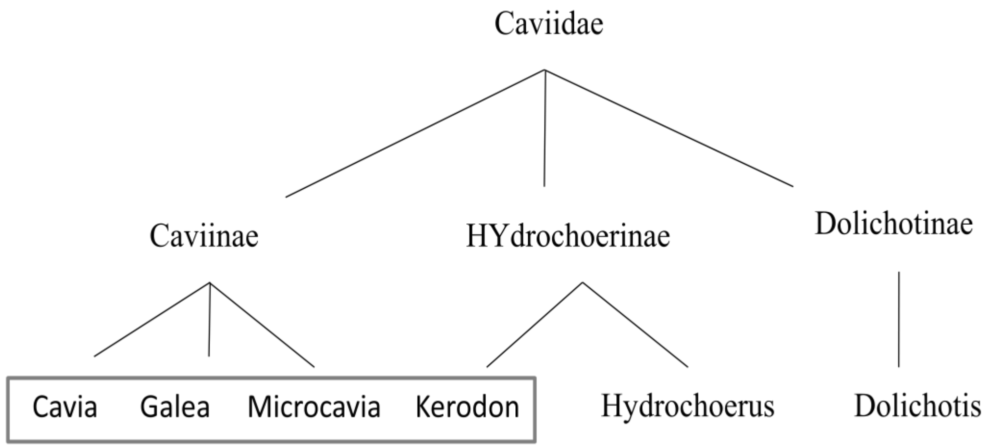

Fig 2. Esquema das relações filogenéticas da família Caviidae, segundo Woods e Kilpatrick (2005). Kerodon que fazia parte de Caviinae (destacado em cinza), agora forma com Hydrochoerus a subfamília Hydrochoerinae.

As relações filogenéticas da família Caviidae não são conclusivas e requerem mais estudos (Cabrera, 1961; Corbet e Hill, 1991; Wilson e Reeder, 1993; McKenna e Bell, 1997; Woods e Kilpatrick, 2005). Classificações taxonômicas baseadas em análises morfológicas e moleculares discordam em relação às posições dos grupos e gêneros na família (Quintana, 1998; Rowe e Honeycutt, 2002; Trillmich et al., 2004; Woods e Kilpatrick, 2005). Além disso, conflitos sobre o número de famílias reconhecidas estão relacionados com os gêneros *Cuniculus* e *Stictomys* que tanto são atribuídos à família Agoutidae (Anderson e Jones,1984) quantosão colocados com os genêros *Dasyprocta* e *Myoprocta* na família Dasyproctidae. Dessa forma, o conteúdo e a relação entre essas

subfamílias e seus gêneros não tem sido adequadamente abordados (Hartenberger, 1985; Nedbal et al., 1994). Estudar a comunicação acústica dessas espécies de interesse filogenético pode fornecer subsídios de caráter acústico, que poderão auxiliar em estudos futuros sobre a posição taxonômica dos grupos e gêneros nessas famílias.

A superfamília Cavioidea e em particular a família Caviidae são um excelente grupo para analisar a hipótese da ligação entre complexidade social e complexidade vocal por várias razões além de sua relação filogenética. Primeiro por apresentarem uma ampla variedade de tipos de estrutura social e respectivos comportamentos que revelam plasticidade comportamental (Ebensperger e Hayes, 2016) e que possibilitam testara relação entre complexidade social e vocal. Pode ser encontrado neste grupo, espécies que vivem em grupos socialmente complexos, até espécies mais solitárias, que só se encontram para o acasalamento e manutenção da prole (Redford e Eisenberg, 1992; Maher e Burger, 2016). Além do tamanho dos grupos sociais (Redford e Eisenberg, 1992; Emmons e Feer, 1997; Nowak, 1999; Maher e Burger, 2016), os sistemas de acasalamento também são variados entre os taxa (Herrera, 2016). Outro aspecto que torna os caviomorfos um grupo interessante para se estudar a relação entre complexidade social e vocal é o uso frequente do canal acústico para comunicação (Eisenberg, 1974; Francescoli et al. 2016). Além da disponibilidade de estudos na literatura sobre os repertórios acústicos das espécies deste grupo, há variações nas estruturas acústicas dos chamados e nos tamanhos dos repertórios (Long, 2007; Barros et al., 2011; Alencar Jr., 2012; Nogueira et al., 2012; Monticelli e Ades, 2013).

Como já abordado anteriormente, entre as espécies de interesse encontram-se a paca (*Cuniculus paca*) e a cutia vermelha (*Dasyprocta leporina*), cujos repertórios acústicos foram pouco explorados (Eisenberg 1974; Dubost, 1988) e um maior conhecimento de seus parâmetros acústicos são necessários para melhor compreender e

analisar a complexidade vocal no grupo de caviidae. Tais informações sobre o repertório acústico destas duas espécies, as quais serão disponibilizadas no estudo, poderão além de permitir testara relação entre complexidade vocal e complexidade social neste grupo de roedores, subsidiar estudos futuros sobre a filogenia de caviomorfos com o uso de caracteres acústicos.

1.3 A paca (*Cuniculus* [*Agouti*] *paca*)

A paca, é o segundo maior roedor caviomorfo, que vive desde a região sul do México até o Paraguai e norte da Argentina, e está distribuída por todo território Brasileiro (Woods, 1984). A espécie mede aproximadamente 80 centímetros de comprimento e pesa em média 10 kg (Smythe, 1987). Apresenta pelagem de coloração marrom-avermelhado, com listras laterais longitudinais esbranquiçadas que se estendem do pescoço até a lombar (Perez, 1992). A cauda é curta, quase imperceptível. As patas são curtas, os dedos são alongados, quatro nas patas anteriores e cinco nas posteriores (Perez, 1992). Na natureza, as pacas são animais que vivem solitárias ou em pares, apresentando comportamento monogâmico (Smythe, 1987). Os machos tentam estabelecer seu domínio sobre as fêmeas, por meio de sinalização comportamental que ocorre durante a corte(Smythe, 1987). Nesta sinalização, o macho lança jatos de urina na fêmea, e os dois correm em círculos antes de estabelecer-se como um par (Smythe, 1987). No entanto, quando a fêmea não está receptiva pode tornar-se agressiva às investidas do macho (Smythe, 1987). A fêmea torna-se ativa reprodutivamente aos nove meses de idade, enquanto o macho atinge a maturidade sexual com um ano (Collett, 1981; Matamoros, 1982). Nessa espécie, o período de gestação dura em média 148 dias ocorrendo o nascimento de apenas um filhote duas vezes ao ano (Guimarães *et al*., 2008). A paca é um animal precocial e logo ao nascer apresenta os olhos abertos e é bem ativo; podendo caminhar e seguir a mãe, além de comer alimentos

sólidos nas primeiras 24 h (Smythe, 1987). O filhote é tolerado pela fêmea até os seis meses de idade aproximadamente (Smythe, 1987). A espécie é territorialista e ocupa uma área de vida de três a quatro hectares, sendo agressivas com coespecíficos do mesmo sexo na defesa deste território (Smythe, 1987). Com relação a sua alimentação, consome frutos, sementes e tubérculos e costuma forragear de forma solitária (Emmons e Feer, 1997; Beck-King *et al.*, 1999). É considerada importante predadora e dispersora de sementes (Beck-King *et al.*, 1999).

Durante o dia a paca permanece em tocas individuais (Smythe, 1987; Lobão e Nogueira Filho, 2011) e defendem suas tocas de coespecíficos tanto em vida livre quanto em cativeiro (Smythe, 1987; Sabatine e Paranhos da costa, 2001). Apresenta atividade noturna, o que pode explicar a escassez de estudos relacionados a seu comportamento (Dubost, 1988; Sabatini e Paranhos da Costa, 2001). Sabe-se, no entanto, que as pacas comunicam-se com coespecíficos através de quatro canais - olfatório, visual, tátil e vocal (Francescoli et al., 2016). Os sinais acústicos, no entanto, foram os mais descritos até o momento dentro do sistema de comunicação da espécie (Eisenberg, 1974). Este fato possivelmente esteja relacionado com a facilidade de sua percepção e registro. No entanto, as características acústicas dessas vocalizações, tais como duração das notas, número de harmônicos, frequências máxima, mínima e fundamental, ainda são desconhecidas, o que impede uma correta discriminação entre os tipos de sons emitidos e os contextos comportamentais relacionados. Eisenberg (1974) registrou cinco tipos de emissões vocais na espécie e uma emissão não vocal, as quais associou a contextos de alarme, agressividade e contato. Este autor identificou que quando assustadas, as pacas emitem um ronco ou rosnado baixo (*snort* or *low growl*), quando ameçadas por coespecífico ou predador batem os dentes (*tooth chattering*) e emitem um rosnado muito forte (*growl*). As

fêmeas utilizam um grunhido baixo (*low grunt*) para chamar seus filhotes. E durante a catação social emitem um gemido baixo (*low whine*).

1.4 A cotia vermelha (*Dasyprocta leporina*)

Esta espécie é um roedor caviomorfo que faz parte da família Dasyproctidae. Essa família é composta por 11 espécies do gênero *Dasyprocta* (Eisenberg, 1989): *Dasyprocta azarae*, *Dasyprocta coibae*, *Dasyprocta cristata*, *Dasyprocta fuliginosa*, *Dasyprocta guamara*, *Dasyprocta kalinowskii*, *Dasyprocta leporina*, *Dasyprocta mexicana*, *Dasyprocta prymnolopha* ,*Dasyprocta punctata* e *Dasyprocta ruatanica* (Wilson e Reeder, 1993). Geralmente essas espécies são alopátricas (Smythe, 1978). A cotia vermelha pesa de quatro a seis quilos (Dubost, 1988), com a cabeça e as patas dianteiras acinzentadas, com dorso vermelho variando de um tom mais escuro para mais alaranjado (Emmons e Feer, 1997). As orelhas são pequenas e a cauda é quase vestigial (Emmons e Feer, 1997). Os membros posteriores são maiores que os anteriores, com quatro dedos nas patas dianteiras e três nas patas traseiras (Emmons e Feer, 1997). Possuem garras ligeiramente arqueadas, indicando habilidade para escavar (Emmons e Feer, 1997). A espécie apresenta dimorfismo sexual, sendo as fêmeas maiores do que os machos (Dubost, 1988).

A cotia vermelha está distribuída desde o sul do México até o norte da Argentina (Eisenberg, 1989). Habita uma ampla variedade de ecossistemas que vai desde ambientes de florestas densas até savanas (Eisenberg, 1989). A espécie vive em pares monogâmicos e geralmente forma grupos familiares compostos de três a cinco indivíduos (Dubost, 1988). O período de gestação varia de 104 a 120 dias (Dubost, 1988). Ocupa uma área de vida de cinco a dez hectares (Silvius e Fragoso, 2003; Jorge e Perez, 2005) e utiliza tocas, troncos de árvores, raízes e vegetação como refúgio ou locais para descanso (Dubost, 1988). A espécie possui hábitos diurnos, é territorialista e desloca- se aos pares ou em grupo,

defendendo seu território contra grupos vizinhos (Dubost, 1988). A dieta principal da espécie é baseada em frutos, frutos secos e sementes (Dubost, 1988). As cotias apresentam comportamento de estocagem (*scatter-hoarding*) e costumam acumular sementes que são enterradas em diversos locais da sua área de vida, para consumo em períodos de escassez,.(Silvius e Fragoso, 2003). Tal comportamento de cavar, esconder e procurar alimentos torna as cotias importantes dispersores de sementes nas florestas em que habitam (Emmons e Feer, 1997). Devido àsua ampla distribuição geográfica e por ser considerada uma espécie abundante, seu estado de conservação é considerado menos preocupante (Emmons e Reid, 2016). No entanto, em algumas regiões, a espécie é muito caçada por populações rurais de baixa renda para o consumo da carne (Emmons e Feer, 1997).

Sobre a cotia da américa central, *Dasyprocta punctata*, é possível encontrar várias informações em relação ao uso do habitat e comportamento (Einsenberg, 1974; Smythe, 1978; Francescoli et al., 2016). Enquanto para a cotia vermelha há pouca informação disponível na literatura tanto sobre sua comunicação, como sobre seu comportamento, o que dificulta a compreensão de estratégias comportamentais que deem suporte à sua conservação. Diversos tipos de sinais químicos, visuais e acústicos estão presentes nesta espécie (Francescoli et al., 2016). Estes sinais acompanham comportamentos tais como os de defesa de território, de expressão de dominância/submissão e de acasalamento (Francescoli et al., 2016).

Com relação ao repertório acústico dos Dasyproctidae, a literatura descreve nove tipos de sinais acústicos de *D. punctata* durante contextos agonísticos ou ameaça (*tooth chattering*, *fight grunts*, *Growl*, *rumble*), alarme (*alarm bark*, *scream distress*) e contato (*purr*, *squeak* and *creak squeak*) (Einsenberg, 1974; Smythe, 1978). Em relação à cotia vermelha, a única vocalização descrita na literatura foi o *hollow grunts*, também denominada *barks* utilizadas em contextos de alarme (Dubost, 1988, Emmons e Feer,

1997). Dessa forma, descrições e informações sobre a estrutura acústica do repertório vocal da cotia vermelha ainda não estão disponíveis na literatura e precisam ser melhor estudadas.

1.5 Informações sobre repertórios vocais de espécies de roedores caviomorfos

A capivara (*Hydrochoerus hydrochaeris*) é o maior roedor do mundo, cuja ocorrência vai desde a América central até o norte da Argentina (Emmons e Feer, 1997). A espécie vive em grupos coesos e estáveis que variam de 10 a 25 indivíduos adultos de ambos os sexos e seus filhotes (Azcarate, 1980; Mones e Ojasti, 1986; Herrera e Mcdonald, 1987, 1989). Esses animais são tidos como territorialistas e apresentam hierarquia de dominância social dos machos em relação às fêmeas, relacionada ao peso corporal (Herrera e Macdonald, 1993). Possuem glândulas anais e uma supra-nasal que estão associadas a marcação de território (Herrera e Macdonald, 1993). O repertório acústico da espécie foi descrito por Barros et al. (2011) com animais mantidos em cativeiro e compreendeu sete tipos de sinais acústicos emitidos em diferentes contextos: isolamento (*whistle*, *cry* e *whine*), contato (*click*), alarme (*bark*), distress (*squeal*) e agonísticos (*tooth chattering* e *cackle*) (Barros et al., 2011). Em outro estudo complementar sobre o repertório acústico da espécie, foram encontradas mais dois chamados emitidos em contexto agonístico (cacarejo) e de côrte (chamado de cópula) (Suzuki, 2016).

Os preás são pequenos roedores, que em ambiente natural vivem em pares de macho-fêmea ou em harém composto por um macho com até três fêmeas e seus filhotes (Asher et al., 2004). Esta composição pode variar, uma vez que há relatos de que em outras populações naturais encontram-se formações de cinco a dez indivíduos adultos de ambos os sexos (Rood e Weir, 1970; Rood, 1972; Redford e Eisenberg, 1992). Essa espécie é territorialista (Rood, 1972), e apresentam poliginia de defesa de fêmeas (Asher e

Sachser, 2001; Asher et al., 2004; Asher et al., 2008). Em grupos de baixa densidade os machos apresentam hierarquia de dominância linear, enquanto em grandes densidades a relação social é caracterizada por associações macho-fêmea duradoura (Sachser, 1986). Em laboratório, as fêmeas apresentam hierarquia de dominância (Rood, 1972) que é relacionada idade apresentando animais mais velhos como mais dominantes (Sachser et al., 1999). O repertório acústico de preás (*Cavia aperea*) foi estudado em grupos cativos de um macho e duas a quatro fêmeas, grupos de animais do mesmo sexo e em pares (macho-macho, fêmea-fêmea e macho-fêmea) (Monticelli e Ades, 2013). O repertório acústico dessa espécie consiste de 10 tipos de sinais sonoros emitidos em situações agonísticas (*whine, scream, teeth chattering, squeal*), amigáveis ou exploratória (*contact calls*), alarme (*alarm whistle, drr*), isolamento (*isolation whistle, tweet*) e corte (*purr*) (Monticelli e Ades, 2011; Monticelli e Ades, 2013). Esses autores relataram um repertório rico em tipos de chamados e contextos, com presença de gradação e formas transitórias e intermediárias.

Cobaia (*Cavia porcellus*) é uma espécie de preá domesticada que vive em pares macho-fêmea ou pequenos grupos poligínicos de um macho e até três fêmeas (Rood, 1972). As cobaias apresentam um repertório acústico muito semelhante ao de *C. aperea* composto por 11 tipos de chamados (Monticelli, 2005). As diferenças encontradas entre essas duas espécies estão relacionadas à estrutura física, contextos de emissão e tamanho do repertório (Monticelli, 2005; Monticelli e Ades, 2013), pois a cobaia emite um chamado adicional (*whistle*) para o tratador (Ades et al., 1994) ou em contexto de antecipação alimentar (Monticelli et al., 2009). Os sinais acústicos foram emitidos nos seguintes contextos: agonísticos (*tooth chattering, whine, squeal, purr*), distress (*squeal, scream, isolation whistle*), isolamento (*contact calls, squeal, isolation whistle*), interações mãe-filhote (*contact calls, purr, tweet*), encontros sexuais (*whine, squeal, scream, contact calls, tooth chatering*), atividades exploratórias (*contact calls, drr*) e contato (*contact calls,*

isolation whistle, squeal) (Berryman, 1976; Monticelli, 2005; Monticelli e Ades, 2013). A riqueza do repertório vocal de cobaia foi relacionada por Eisenberg (1974) a flexibilidade do repertório, já que os indivíduos podem mudar as sílabas para expressar diferentes motivações em diferentes contextos.

Mocós (*Kerodon rupestris*) são pequenos roedores sociais que vivem em haréns compostos por um macho e três a quatro fêmeas adultas e seus filhotes (Lacher, 1981; Mares e Ojeda, 1982; Nowak, 1999). Esses animais apresentam poliginia de defesa de recursos (Lacher, 1981) e em cativeiro hierarquia de dominância linear entre fêmeas (Lacher, 1981). Seu repertório acústico é composto por 12 sinais sonoros (Alencar Jr, 2012) emitidos durante contextos de exploração/forrageamento (chorinho, drr, có, estalido), alerta (asobio de alarme, drr, silvo) e interações agonísticas (ganido, grito, ronco, arfar, assobio de alarme, bater de dentes, tamborilar). É possível que outros sinais venham a ser descritos quando se estudar as interações entre adultos e filhotes e as respostas de filhotes à situação de isolamento social ou da mãe, já que foram observados apenas animais adultos.

Degu *(Octodon degus)* é um roedor semifossorial que vive em grupos de dois a três machos e de duas a cinco fêmeas (Fulk, 1976). São diurnos e apresentam hierarquia social, além de apresentarem poliginia e promiscuidade como sistemas de acasalamento (Soto-Gamboa et al., 2005; Quirici et al., 2010). Segundo alguns autores, possuem repertórios complexos tanto comportamental quanto vocal (Braum et al., 2003; Long , 2007). Seu repertório acústico é composto por 15 tipos de vocalizações emitidas em contextos agonísticos (whine, groan, grunt, tweet, chirp), de alarme (wheep, squeal, bark), distress (pip), contato (chaff, trill, low whistle, chitter) e brincadeira (loud whistle, warble).

Coruro *(Spalacopus cyanus)* são roedores fossoriais altamente sociais que vivem em grandes colônias compostas por grupos de cinco a quinze indivíduos adultos que

apresentam sistemas de acasalamento poligínico (Begall et al., 1999). O repertório vocal consiste de 12 tipos de sinais acústicos (Veitl et al., 2000) que são emitidos em contextos agressivos (cluck I, cluck II, teeth chatter), contato (cooing, twitter I, twitter II e squeak), distress (cluck III, squeal), alarme (trill) e corte (creaking e scream).

Chinchila (*Chinchilla lanigera*) são pequenos roedores noturnos e territoriais que vivem em colônias (Spotorno et al., 2004). As fêmeas dessa espécie são mais agressivas que os machos na defesa territorial (Spotorno et al., 2004). Os animais desta espécie apresentam um repertório acústico composto por dez tipos de chamados emitidas em contextos de alarme (alarm call), agonístico (snort, blocking, scream, teeth chatter), contato (attract call, sucking sound, offspring contact), exploratório (position call) e sexual (mating call) (Bartl, 2006).

Tuco tuco *(Ctenomys talarum)* é um pequeno roedor subterrâneo que vive em galerias individuais (Busch et al., 1989), e forrageiam tanto dentro dos seus túneis como acima do solo. Essa espécie é solitária e territorial (Busch et al., 1989) e apresentam um repertório vocal composto por cinco tipos de chamados emitidos em contextos agonísticos (tooth grinding, tuc tuc e grunds), distress (grunds) e durante a côrte (vocalização de acasalamento de macho e vocalização de acasalamento de fêmeas). As vocalizações possuem características acústicas, como freqüências baixas, que permitem uma melhor transmissão no subsolo (Schleich e Busch, 2002).

1.6 OBJETIVOS

Testar a relação entre complexidade social e vocal em 10 espécies de Cavioidea (*Cavia aperea, C. porcellus, , Kerodon rupestres, Cuniculus paca, Dasyprocta leporina, Ctenomys talarum, Spalacopus cyanus, Chinchilla lanígera, Octodon degus* e *Hydrochoerus hydrochaeris*) e analisar os papéis que fatores morfológicos, reprodutivos e ecológicos podem exercer na complexidade vocal deste grupo. Descrever e analisar os repertórios acústicos de duas espécies de caviidae, a paca (*Cuniculus paca*) e a cotia (*Dasyprocta punctata*) para contribuir no conhecimento e discussão sobre aspectos sociais e ecológicos que envolvem sua comunicação sonora.

1.7 HIPÓTESES

H1. O repertório acústico de *Cuniculus paca* será composto principalmente por chamados associados a contextos agressivos.

H2. Há diferenças vocais nos chamados de *Cuniculus paca* passíveis de discriminação de machos e fêmeas.

H3. A paca apresenta um repertório simples como esperado para espécies solitárias.

H4. O repertório acústico de *Dasyprocta leporina* será composto principalmente por sinais associados a contextos agonísticos.

H5. Cotias exibem chamados que apresentam diferenças vocais suscetíveis de discriminação entre machos e fêmeas.

H4. Há relação direta entre complexidade vocal e complexidade social em caviomorfos.

H5. Quanto maior a complexidade social, das espécies, maior será a complexidade vocal das mesmas.

CAPÍTULO 1

Does the acoustic repertoire of spotted paca (*Cuniculus paca*) can explain the species sociality?

Abstract

The acoustic communication has revealed many signs of species social behavior and may be useful to track traces of sociality in spotted paca (*Cuniculus paca*). This species is a nocturnal, territorial hystricognath rodent that lives alone or in monogamous pairs. However, there are some sights of groups in the wild and successful captive groups in Neotropical wild farms. We aim to investigate the presence of sociality in 51 captive spotted pacas by describing the species acoustic repertoire and discuss our findings in light of the species' social and territorial behavior. For this propose, because the spotted paca has solitary and territorial habits we predict to find a simple vocal repertoire, composed of few acoustic signals, mainly related to agonistic contexts. In opposite, if spotted paca remains social skills and can be breed in social groups in captivity, the acoustic repertoire of animals will be more plastic and shows more acoustic complexity. We found that the acoustic repertoire of spotted paca consists of six vocal types and one mechanical signal. We confirmed our expectations that the spotted paca's repertoire is mainly composed of agonistic vocalizations, possibly related to territorial defense. Nevertheless, we observed evidences of complexity in the vocal repertoire of this solitary animal, such as gradations and the production of combined vocal types. Our study showed plasticity in the species' repertoire by the presence of gradations and combinations of sounds that can diversify its signals and improve communicative performance during social interactions.

Keywords: bioacoustics, caviomorph, communication, hystricognathi, rodent, vocal complexity.

Introduction

The acoustic communication has promoted information about social strategies of many mammals' species as subordinate calls (Silk et al. 1996; Nogueira et al. 2016), greeting calls (Laporte et al. 2010) and contact calls (Sousa-Lima et al., 2002, 2008; Nogueira et al. 2012;Dos Santos et al., 2013). These acoustic strategies present in many mammals' societies have been valuable to better understand the species social organization and may also help to clarify the evolution of sociality traits. The evolution of sociality is related with predation risk, diurnal habits, body size (Topping et al. 1999), and also is correlated with burrowing in hystricognath rodents (Ebensperger and Blumstein 2006). Nevertheless, species have been impacted for human-induced rapid environment change (HIREC) (Sih 2013) as climate change, hunting or habitat fragmentation. These challenges may enforce changes of species natural behavior to persist in a modified environment being more plastic (Price et al. 2003; Sih et al. 2011) and maybe masking a natural behavior useful in other time and space.

Spotted paca (Cuniculus paca, Linnaeus 1766) is the second largest hystricognath rodent occurring from Southern Mexico to Northern Argentina, and it is widely distributed in Neotropical countries (Emmons 2016). The species is nocturnal and live in home ranges of three hectares, alone or in monogamous pairs during reproductive periods (Smythe 1987). Individuals can behave territorially and are aggressive towards conspecifics of the same and opposite sex to defend their burrows in wild (Smythe 1987). The social organization of this species remains controversial. Despite the apparent territorial intolerance among conspecifics (Smythe 1987; Sabatini and Paranhos Da Costa 2001)

there have been some sightings of spotted paca groups in the wild (Nogueira-Filho personal communication). In addition, wild farmers have been successful assembling juveniles and breed them in captive groups (Smythe and Brown de Guanti 1995; Nogueira-Filho and Nogueira 1999).

The social complexity hypothesis for communication states that solitary animals show lower diversity of communicative signals than species living in groups with complex social systems (Freeberg et al. 2012). Eisenberg (1974) reported six types of acoustic signals, mostly related to contact (*low grunt* and *low whine*), alarm (*snort* and *low growl*) and aggressiveness (*tooth chattering* and *very loud growl*). However, no data is available about the acoustic parameters of these calls to better investigate vocal complexity as a cue for more sociality in spotted paca. Thus we aimed to extend the knowledge on the spotted paca acoustic communication to investigate the presence of sociality' signs and discuss our results in the light of the species' habits: nocturnal, burrowing, territorial and solitary – only living in pairs during reproductive seasons and clarify the group assembly possibility in captive. For this propose, because the spotted paca has solitary and territorial habits we predict to find a simple vocal repertoire, composed of few acoustic signals, mainly related to agonistic contexts (Blumstein and Armitage 1997). In opposite, if spotted paca remain social skills and can be breed in social groups in captivity, the acoustic repertoire of animals will be more plastic and shows more acoustic complexity.

Materials and Methods

This work followed the principles of laboratory animal care (NIH publication No. 86-23, revised in 1985) and was approved by the Committee on Animal Research and Ethics of the State University of Santa Cruz, under protocol # 010/11.

Study area and subjects

The study was carried out at a commercial spotted paca farm at Camaçari, state of Bahia, Brazil. We recorded vocalizations and behaviors of 51 individuals: 42 adults (26 females and 16 males) and nine young (six females and three males). All individuals were born and raised in captivity. The adult individuals ranged from two to four years old and the young from 15 days to four months old. Because the females give birth to one pup at a time, farmers usually breed animals in groups to improve reproduction rates, following Smythe and Brown de Guanti's (1995) recommendation. Thus, the animals were housed in groups of one male and four females in 20 breeding pens and four maternity pens. Five out of 20 breeding pens had one to two young. The maternity pens housed only the mother and its single weaned pup.

Each pen consisted of a 6 m² (3m long x 2m wide) area, with concrete floors, surrounded by 1-m-high brick walls supported by a wooden pole. The ceiling was covered with tiles and surrounded by a wire mesh fence. All pens had two wooden shelters (1.5m long X 1.0m wide X 1.5m high), a water tank (0.6m long X 0.3m wide) and two feeders (1.0m long X 0.3mwide). During observations at night, we used red lights to allow better visualization of animals and minimizing disturbances to them. The animals received a regular diet of a commercial ration for rabbits (200 g / animal), seasonal fruits, mineral salt and water *ad libitum*.

Data collection

Both calls and behaviors were recorded simultaneously at 1.5 m distance from the animals. The observation sessions usually took place between 4 pm and 6 pm, the period of the highest activity of the captive spotted paca. We also observed the animals on three other occasions: during cleaning of the pen (between 11 am and 1 pm), during handling for medical procedures (between 11 am and 1 pm) and when the animals were fed (4 pm).

We recorded the acoustic signs *ad libitum* (Altman 1974) using a Sennheiser ME-66 directional microphone (Wedemark, Germany) and a Marantz PMD 670 (Sagamihara, Japan) digital recorder (recorder settings: WAV format, mono mode, 48 kHz sampling rate, and 16-bits resolution). The observer started to record the animals when they were actively emitting sounds and kept recording until any sound emission was produced up to its end or after an interval of 1 minute with no sound emission. Because the animals are not highly vocal, we could not register calls from all individuals housed in the breeding pens. The data collection totalized 90 hours of recording and observations.

To stimulate the emission of isolation or contact calls between mother and young, we isolated four mothers from their young by using a wooden barrier (122cm X 75cm X 2.5 cm), which acted as a barrier to physical and visual signals, but not loss of auditory and chemical contact. The animals' keeper was responsible for setting up the barrier between mother and young. The observer started to record the acoustic signs just after the keeper isolated the animals. Each mother/pup pair was separated from each other only once. This recording/observation session lasted 30 minutes per pair.

Acoustic analysis

Spectrograms were generated and analyzed with Raven Pro Software version 1.5 (Cornell Lab of Ornithology, Ithaca NY) using the following settings: Hann window type, 1460 samples window size, 90% overlap in the time domain, and 4096 DFT size. First we categorized putative call types by ear and by visual inspection of spectrograms. The smaller vocal units of the calls were termed elements and were defined as a continuous sound without interruption (*sensu* Feng et al. 2009 and Barros et al. 2011). For each element we measured the following parameters: minimum frequency (Hz), maximum frequency (Hz), dominant frequency (Hz), duration (s), and the number of harmonics below 1 kHz. Single elements could be emitted repeatedly, intercalated with intervals of

silence, forming phrases (*sensu* Hauser 1989). For sounds emitted as phrases, we measured the duration of the inter-element intervals and the rhythm (the number of elements divided by the total phrase duration). We calculated the average interval between the elements and considered one element as part of another phrase when the interval between subsequent elements was longer than the average interval (*sensu* Barros et al. 2011). We also examined the spectrogram for the presence of gradation (gradual transition from one signal to the next - Hauser 1996), combination of sounds (combination of different vocalization types in sequences - Crockford and Boesch 2005), formants (vocal tract resonance frequencies -Fitch 1997) and deterministic chaos denoted as broadband energy on the spectrogram with residual periodic energy (Fitch et al. 2002). To calculate the rate of call emission per hour (hourly rates) we divided the total numbers of each vocal type by the total observation time (90 h).

Statistical analysis

To test the validity of the putative call types that we categorized by ear and by visual inspection of the spectrograms, we conducted a Discriminant Function Analysis (DFA) based on the five acoustic parameters that were measured. To avoid including more than one sample of the same element type from the same recording section in the analyses, we randomly chose 50 elements of each vocal type from the total 3,341 elements recorded for acoustic measurements, totaling 400 samples. All vocalizations chosen had high signal quality (low background noise and no overlap among calls from different individuals). Before conducting the DFA analysis we standardized the variables (by subtracting the mean of the variable from each data point and dividing the result by the variable's standard deviation) to avoid the spurious attribution of weights to acoustic parameters measured in different units (Noy-Meir et al. 1975). To test the significance of the discriminant model we performed a Multivariate Analysis of Variance (MANOVA). Further, to determine

whether it was possible to predict each vocal type correctly based on the measured acoustic parameters (independent variables), we performed cross-validation analyses, reporting the DFA accuracy as the proportion of elements correctly assigned to each vocal type. We performed a binomial test to evaluate whether the proportion of the success of the cross-validation analysis was higher than that expected by chance.

We also evaluated the pattern of emissions of *clicks* and *snores* emitted in sequence (phrases). This procedure was done because of the similarity in the acoustic structure of the single elements of these emissions that occur in different behavioral contexts. Therefore, we calculated the maximum frequency of the first element of the phrase (Hz), the inter-element interval (s) and the rate of emission for each vocal type (number of elements per second). Subsequently, we calculated the absolute differences of each variable measured (maximum frequency of the first element of a sequence, the inter-element interval, and the rate of the emission) between *clicks* and *snores*. Then, we compared these observed differences with the distribution of differences expected by chance (under the null hypothesis that sequences of *clicks* and *snores* do not differ from each other). To calculate the associated *P*-values, we generated the distribution of the differences expected by chance by 1000 Monte Carlo simulations (Monte Carlo simulation, Manly 1997). To avoid any possible artifact due to multiple comparisons, we used a false discovery rate (FDR) correction, which corresponds to a less conservative and more powerful correction than Bonferroni’s (Benjamini and Hochberg 1995).

All analyses were performed in R software version 2.15.0 (R Development Core Team 2014) using the packages ‘MASS’ version 7.3-18 (Venables and Ripley 2002) and ‘FactoMineR’ version 1.25 (Husson et al. 2014), with a significance level of $\alpha = 0.05$.

Results

Eight putative calls were found based on the animals' observations and the visual inspection of the spectrograms (Figure 1). The hourly rate of emissions showed that *roar* (3.3 calls/s) was the most frequent vocal type, followed by *snore* (3.2 calls/s), *growl* (2.6 calls/s), *bark* (1.8 calls/s), *cry* (0.7 calls/s), *tooth chattering* (0.9 calls/s), *groan* (0.7 calls/s) and *click* (0.4 calls/s).

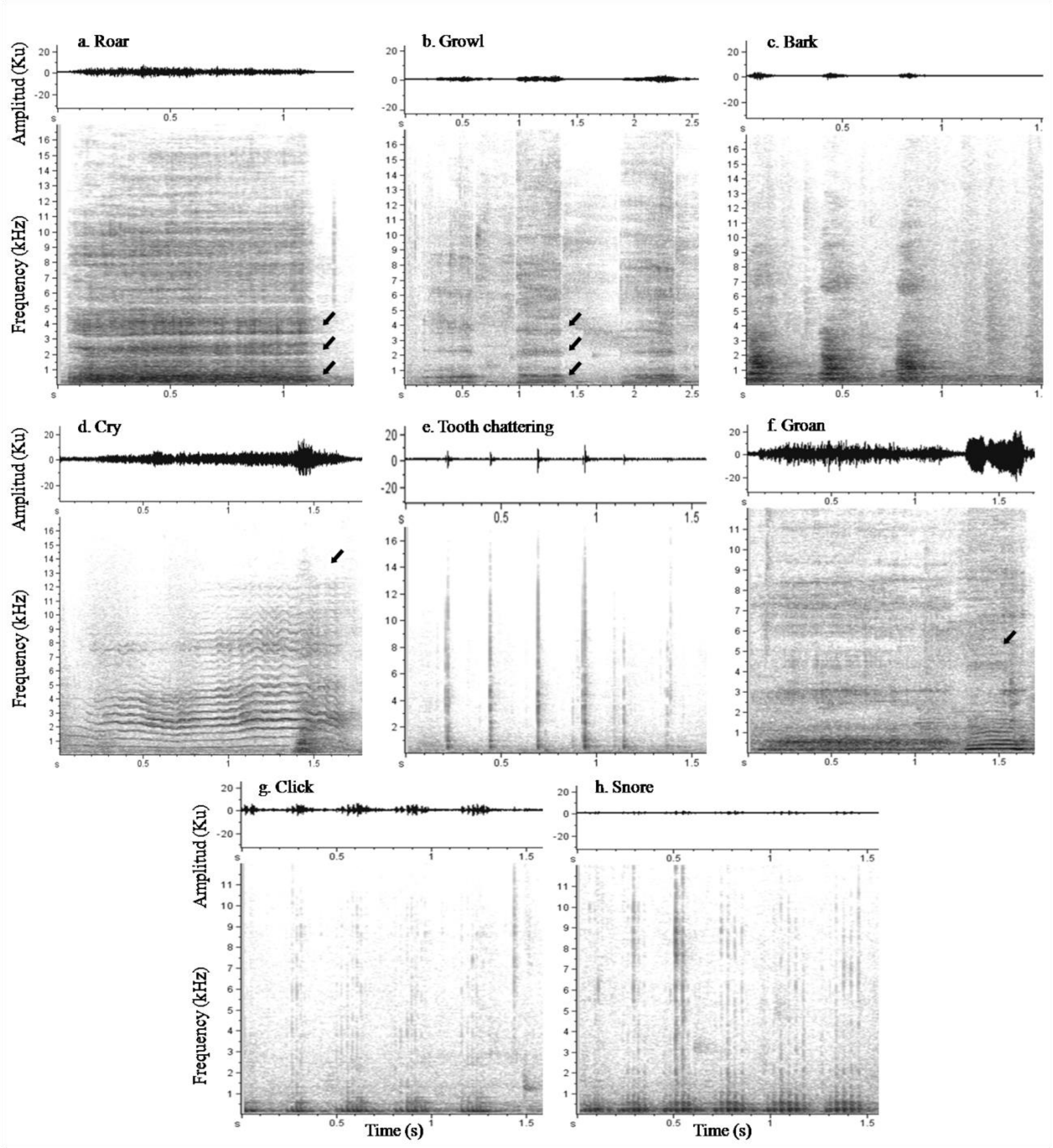

Figure 1. Spectrograms and time series of spotted paca vocalizations. The arrows in boxes *a* and *b* indicate formants, and in box *d* indicate a sudden transition to chaotic dynamics of sound production, resulting in deterministic chaos. Combination of sounds between roar and groan calls in box *f*, arrow indicate groan call. Gradation between growl-bark (box *b* and *c* respectively) and click-snore (box *g* and *h* respectively) results from similarity in the acoustic structure between these calls.

The DFA, based on the five acoustic parameters measured, discriminated six (*roar, growl/bark, tooth chattering, groan, snore/click* and *cry*) out of the eight call types initially proposed (Wilks Lambda = 0.013; *P* <0.001, n = 400; Table 1).

Table 1 Mean ± standard deviation of each acoustic parameter measured in spotted paca vocalizations. Coefficients of the two main discriminant functions (DF) indicating the relative contribution of each acoustic parameter (variable) for the distinction among vocal types and the percentage of notes correctly attributed to each call type in the cross validation. The N corresponds to the number of emissions analyzed in each category. The values in bold indicate the parameters with higher loading for the discriminant functions (DF1 and DF2).

Vocal type	N	Duration (s)	Dominant frequency (Hz)	Min. frequency (Hz)	Max. frequency (Hz)	Harmonic number at 1 Hz	Cumulative percentage of explained variance DFA	Cross-validated DFA
Roar	50	1.5 ±0.9	672.0 ±589.0	95.2 ±66.2	15922.8 ±8712.8	-	-	76
Growl	50	0.4 ±0.2	392.1 ±242.7	114.9 ±110.1	13577.3 ±9173.9	-	-	36
Bark	50	0.2 ±0.07	531.8 ±348.6	107.5 ±68.9	15466.6 ±7321.6	-	-	68
Tooth chattering	50	0.9 ±0.02	589.0 ± 157.2	66.2 ± 124.5	8712.8 ± 6858.3	-	-	78
Groan	50	0.5 ±0.2	359.8 ±191.7	162.5 ±96.4	5782.4 ±6814.7	3.0 ±1.0	-	92
Snore	50	0.3 ±0.05	190.8 ±82.0	65.1 ±44.2	8702.4 ±7892.4	-	-	36
Click	50	0.2 ±0.05	146.0 ±28.3	47.8 ±16.7	2863.4 ±3820.3	-	-	74
Cry	50	1.8 ±0.7	1676.9 ±711.4	280.8 ±139.5	14434.7 ±4979.6	1.4 ±0.6	-	92
DF1	-	0.26	0.25	**0.62**	-0.24	**2.77**	72.0	-
DF2	-	**1.17**	**0.67**	0.23	0.23	-0.69	91.0	-

The first two discriminant functions explained 91% of variance among the vocalizations. The number of harmonics under 1 kHz and the minimum frequency were the variables that most contributed to the first discriminant function, whereas the duration and the dominant frequency were the variables that most contributed to the second function (Table 1). The cross-validation correctly attributed the vocal categories with an accuracy of 66%, which was significantly higher than the 13% (or 1/8) expected by chance (binomial test: Ps <0.001). The accuracy of cross-validation for the eight vocal categories ranged from 36% (*snore* and *growl*) to 92% (*cry* and *groan*) (Table 1). In DFA analysis, *snore* and *growl* vocalizations were confounded with *clicks* and *barks*, respectively, possibly because of the structural similarity resulting from gradation between them (Table 1; Figure 1). Although *snores* (average freq max = 5.98; SD = 6.81) have been confounded with *clicks* in the cross-validation analysis, these calls were emitted in different behavioral contexts (Table 2) and also differed with respect to the maximum frequency of the first element that is emitted in sequences or phrases (Table 3; Figure 1), with snores presenting higher frequencies. The sequences formed by both calls, however, did not differ from each other with respect to the inter-element interval, and nor did the rhythm of element emission (Table 3). These results led us to consider these calls as two different types of vocalizations.

Table 2. Description of context and possibly communicative function of the vocalizations of spotted paca associated with age (A: adult and J: juveniles) and sex (M: male and F: female).

Call	Communicative Function	Age and sex category	Context
Roar	Agonístic	A, J, M, F	Loud and harsh sound emitted as a single element or in sequences of two to five elements. During these emissions, the animals are very close to one another, and usually adopt alert posture, exhibiting pilo-erection and open mouth. Recorded during agonistic encounters mainly by defense of burrows, and during capture for management procedures. Roar call was produced once by one female during copula avoidance. The possibly function is a menace to repel conspecifics or keeper.
Snore	Agonistic	A, M, F	Low vocalization emitted in sequences of three to seven elements. They were produced when an animal approaches burrows or food of another animal or a human being during pen cleaning. The animal assumes alert posture with minimal movements of mouth.
Tooth chattering	Agonístic	A, J, M, F	A mechanical signal produced by the clash of upper and lower incisors. This sound is produced in sequences of two to five elements. The animals produce the sound during defense of burrows, during mother/pup isolation and during human-being presence. This sound usually does not evolve to an attack if the receiver animal promptly runs away from the sender or displays a submissive posture (runs away slowly with the head and forelegs slightly down).
Growl/bark	Agonistic/alarm	A, J, M, F	A harsh sound produced as a single element or in sequences of two to five elements. Growls were recorded in agonistic encounters addressed only to conspecifics. The call was emitted mainly during defense of burrow or resources. Barks are produced alone or in sequences of two to six short elements. These vocalizations were emitted only when an animal was captured for medical procedures or during environmental disturbance (loud noises or presence of unfamiliar human-beings). Animal assumes alert posture, pilo-erection and vocalizes. Sometimes the animal jumps, trying to escape from the situation. A behavioral response from a conspecific, immediately after the call is released, can lead to freezing and hiding in burrows. The possible function of

			growl/bark is to menace and alarm conspecifics, respectively.
Groan	Agonistic/alarm	A, J, M, F	Noisy vocalization with three to four harmonic structures. These calls are emitted in a sequence of two to three elements. Produced when an animal is injured during agonistic encounters or during a net capture. This call possibly functions to express pain or to alert conspecifics to the negative experience.
Click	Contact	A, M, F	Clicks are very low vocalizations emitted in sequences of three to eight elements This vocalization was produced before feeding and when the keeper approaches for food delivery.
Cry	Contact	J, M, F	Loud sound with broadband noise that presents harmonic structures emitted singly or in a sequence of two to four elements. Produced only by young during mother-pup separation. The possible function is to reestablish contact with mother.

Table 3. Comparison of acoustic parameters of the sequences formed by *clicks* and *snores*. The *P*-values were adjusted by FDR correction and significant differences are highlighted in bold. The N corresponds to the number of sequences analyzed in each category.

Variable	N	Click (Mean ± SD)	Snore (Mean ± SD)	Observed difference	Expected difference	*P* corrected
Max. freq. initial	35	1.51 ± 2.48	5.98 ± 6.81	4.47	1.05	**0.006**
Inter-elements interval	28	0.044 ± 0.042	0.048 ± 0.039	0.003	0.008	0.388
Rhythm	12	3.95 ± 1.44	3.52 ± 0.56	0.43	0.35	0.273

The vocalizations here described were produced in aggressive, alarm and contact contexts (Table 2). Most calls produced during aggressive behavioral contexts showed low dominant frequencies (under 1676.9 Hz; Table 1). Inspection of the spectrograms revealed complex acoustic structures as nonlinear phenomena and deterministic chaos in *roar, growl/bark* and *cry* vocalizations (Figure 1). We also observed structures resembling formants in *roar* and *growl/bark* calls (Figure 1). In addition, we observed complex structures such as gradation (*click/snore* and *growl/bark*) and a combination of sounds between *groans* and *roars* (Figure 1).

Cry and *clicks* were the only non-agonistic calls in the species repertoire. *Clicks* were emitted in the context of feeding, probably as a contact call (Table 2). In mother-offspring separation, only the *cry* call (Figure 1) was emitted by offspring, suggesting a

contact function (Table 2). During *cry* emissions by the offspring, the mother remained agitated, moving back and forth into the maternity pen, sniffing the air and the wooden barrier between them. The mother could also produce *tooth chattering* and *bark*s in response to the offspring *cry* call. On one occasion, we observed one of the mothers performing thumping displays (when the animal beats the ground with its hind legs) during this separation period.

Discussion

The captive spotted paca repertoire comprises seven vocalizations: six vocal types and one non-vocal. In accordance with our expectation, the repertoire of the species is mostly produced under agonistic contexts, which is possibly associated with animals' territoriality and defensive behavior. In addition, we also confirm the presence of structural complexity in species' repertoire, refuting our first prediction that suggests a simple repertoire for a socially simple species.

The spotted paca vocalizations and associated behavioral contexts found in our study are aligned with the previous description of the species' vocal repertoire reported by Eisenberg (1974) in a study of free-range animals. This author found a small number of calls (n=6) for the species, and most of the vocalizations were related to agonistic contexts (Eisenberg 1974), as observed here. This previous description, however, did not include the acoustic structures of the calls, which prevented more accurate comparisons about complexity.

The spotted paca is known to defend its territory, particularly its burrow, and is usually aggressive towards conspecifics to shows its dominance (Smythe 1987; Sabatini and Paranhos Da Costa 2001). This behavior may explain the great proportion of agonistic vocalizations reported in the present study, and is in accordance with previous

reports about solitary species that mainly use acoustic communication for territorial defense and/or to find partners for reproductive purposes (rufous-and-white wren, *Thryothorus rufalbus*: Mennil and Vehrencamp 2008; swift fox, *Vulpes velox*: Darden and Dabelsteen 2008).

The presence of gradation between some call types may have precluded a prompt distinction among vocalizations by DFA and cross-validation analysis, as in the case of *clicks*/*snores* and *growls*/*barks.* In clicks and snores, we registered differences in the maximum frequency in the first element. In *snores* this frequency is greater than in *clicks*, possibly related to their different function. While snores were emitted in agonistic function, *clicks* were emitted during feeding, possibly with a contact function, as reported above. The presence of gradation in elements of vocalizations can communicate different emotional states (Einsenberg 1974) and seems to have an important role for hystricognathi species because it is a common feature in the repertoire of 17 species in this group (Einsenberg 1974). *Growls* and *barks*, in turn, seemed to be part of a continuous gradation that was related to duration. These calls were emitted when animals were close to each other, as expected for graded calls (Owren and Rendall 1997), thus, the animals can use visual displays such as alert posture and pilo-erection (Francescoli et al. 2016), to aid the receiver to distinguish among behavioral contexts involved in this vocal emission.

The presence of gradation in animals' acoustic repertoire is believed to increase the amount of information conveyed per category of calls (Hauser 1996). These characteristics of vocalizations possibly increase the complexity of apparently simple repertoires composed of few acoustic signals, as is possibly in the case of the spotted paca repertoire. In addition, the production of acoustic variability in calls can minimize habituation of receivers, increasing the chances of eliciting unconditioned responses

(Owren and Rendall 1997). *Growls* are produced during defense of burrows, and in this particular case, gradations may be advantageous. The production of a variable sound with the potential to reduce the habituation of the listeners, avoiding that they ignore the call, may improve the call's effectiveness and promote of avoidance or flight in the receiver.

In the present study, another source of complexity on the species repertoire is the presence of nonlinear phenomena resulting from the aperiodic vibration of the vocal apparatus, such as deterministic chaos (Fitch et al. 2002). Deterministic chaos seems to be present in the *roar*, *growl/bark,* and *cry,* which were emitted in aggressive, alarm and contact (young/mother) contexts. Chaotic calls are unpredictable, and are also believed to be harder to ignore than calls produced under a more regular vibratory regime (Fitch et al. 2002; Blumstein et al. 2008; Blumstein and Récapet 2009). Thus, these calls may be very effective in contexts of high risk, avoiding receiver's habituation to the call in situations as those described for the occurrence of *roar*, *growl/bark,* and *cry*. In *roar* and *growl/bark* vocalizations, formant-like structures seem also to be present (Figure 1). Formants usually play an important role in individuals' recognition, because they reflect details of individual vocal-tract anatomy and body size (Owren and Rendall 2001). The presence of formants may also allow receivers to assess the caller's age, sex and maturity (Reby and McComb 2003a; Rendall et al. 2004), which could help the recognition of the reproductive condition of male/female in the spotted paca. However, we do not have morphological measures of the spotted paca's vocal tract to allow more accurate analyses or to certify if these structures are indeed formants. Therefore, other studies and measures need to be carried out to better characterize these features.

Our finds suggest that the vocal repertoire of spotted paca (6 calls, 3 contexts) seems to be simple, in relation to the number of calls and the behavioral contexts in

which they occur in comparison to the other solitary caviomorphs including *Ctenomys talarum* (4 calls, 3 contexts: Schleich and Busch 2002) and more social species such as *H. hydrochaeris* (8 calls, 5 contexts: Barros et al. 2011; Suzuki 2016), *Cavia aperea* (9 calls, 6 contexts: Monticelli and Ades 2013), *C. porcellus* (10 calls, 7 contexts: Monticelli 2005), *Kerodon rupestris* (10 calls, 6 contexts: Alencar Jr. 2011); and *Octodon degus* (14 calls, 7 contexts: Long 2007). Conversely, the gradations and combination of sounds observed here are usually expected for more complex repertoires in species with higher levels of social organization (see Freeberg et al. 2012). Although the spotted paca social system is characterized as less complex solitary individuals, found in monogamous pairs only during reproductive periods (Smythe 1987), we found cues of a complex structural repertoire for this species. We raise two possible alternatives to explain the presence of a complex vocal repertoire in this solitary species. The first addresses to the characterization of the species as solitary. Although most studies reports the observation of one or two individuals, Nogueira-Filho and Nogueira (1999) report the presence of about four free-ranging individuals, including adults and young, in the Atlantic Forest in São Paulo state. The authors suggest that the solitary habit reported for the species in the literature may be related to the intensity of hunting pressure, a HIREC (Sih, 2013). Thus, the complexity of the acoustic repertoire of the spotted paca could be due to complex social organization, still under-observed. An alternative explanation may be that the breeding system adopted for farmed animals, keeping them in groups of five animals, can resulted in more complexity on paca's acoustic repertoire, imposing more behavioral plasticity. These arrangements may give the animals' the opportunities to diversify their repertoire. Freeberg (2006) found relationships between group size and chick-a-dee's call complexity. The author experimentally manipulated groups of chickadees and compared the vocal complexity

with non-manipulated groups in the field. The study indicated that birds from larger groups usually emitted more complex calls than birds in smaller groups (Freeberg 2006). Regardless of which alternative is better to explain the presence of complexity in the spotted paca's acoustic repertoire, our study did show plasticity in the species' repertoire by the presence of gradations and combinations of sounds that can diversify its signals and improve communicative performance during social interactions.

Acknowledgements

We thank the commercial spotted paca farm for supporting this research and the Laboratório de Etologia Aplicada at UESC staff for helping with bioacoustics equipment and logistic analysis. This study was supported by PNPD/CAPES (#2951/2010). SGCL and SSCN were supported by CNPq.

Literature Cited

Alencar Jr., R. N. 2011. O repertório acústico de um especialista de rochedos da Caatinga, o mocó. Master Thesis; Universidade de São Paulo, São Paulo, Brazil.

Altmann, J. 1974. Observational study of behaviour: sampling methods. Behaviour 49:223-265.

Barros, K. S., Tokumaru, R. S., Pedroza, J. P. and Nogueira, S. S. C. 2011. Vocal repertoire of captive capybara (*Hydrochoerus hydrochaeris*): structure, context and function. Ethology 116:83-93.

Benjamini, Y. and Hochberg,Y. 1995. Controlling the false discovery rate: a practical and powerful approach to multiple testing. Journal of the Royal Statistical Society 57:289-300.

Blumstein, D. T. and Récapet C. 2009. The sound of arousal: the addition of novel non-linearities increases responsiveness in marmot alarm calls. Ethology 115:1074-1081.

Blumstein, D. T., Richardson, D. T., Cooley, L., Winternitz, J. and Daniel, J. C. 2008. The structure, meaning, and function of yellow-bellied marmot pup screams. Animal Behaviour 76:1055-1064.

Blumstein, D. T., and Armitage, K. B. 1997. Does sociality drive the evolution of communicative complexity? A comparative test with ground dwelling sciurid alarm calls. The American Naturalist 150:179-200.

Crockford, C. and Boesch, C. 2005. Call combinations in wild chimpanzees. Behaviour 142:397-421.

Darden, S. K., and T. Dabelsteen. 2008. Acoustic territorial signalling in a small, socially monogamous canid. Animal Behaviour 75:905-912.

Dos Santos, E.; Tokumaru, R. S.; Nogueira Filho, S. L. G. and Nogueira, S. S. C. 2014. The effects of unrelated offspring whistle calls on capybaras (*Hydrochoerus hydrochaeris*). Brazilian Journal Biology 74:171-176.

Ebensperger, L. A. and Blumstein, D. T. 2006. Sociality in New World hystricognath rodents is linked to predators and burrow digging. Behav Ecol 17:410-418.

Eisenberg, J. F. 1974. The function and motivational basis of hystricomorph vocalizations. Symposia Zoological Society London 34:211-247.

Emmons, L. 2016. *Cuniculus paca*. The IUCN Red List of Threatened Species 2016: e.T699A22197347. Downloaded on 09 October 2016.

Feng, A. S., Riede, T., Arch, V. S., Yu, Z., Xu, Z. M., Yu, X. J. and Shen, J. X. 2009. Diversity of the vocal signals of concave-eared torrent frogs (*Odorrana tormota*): evidence for individual signatures. Ethology 115:1015-1028.

Fitch, W.T., Neubauer, J.and Herzel, H. 2002. Calls out of chaos: the adaptive significance of nonlinear phenomena in mammalian vocal production. Animal Behavior 63:407-418.

Fitch, W. T. 1997. Vocal tract length and formant frequency dispersion correlate with body size in rhesus macaques. The Journal of the Acoustical Society of America 102:1213-1222.

Francescoli, G., Nogueira, S. and Schleich, C. 2016. Mechanisms of social communication in caviomorph rodents. In: Sociobiology of caviomorph rodents: an integrative approach (eds L. A. Ebensperger and L. D. Hayes), John Wiley & Sons, Ltd, Chichester, UK.

Freeberg, T. M., Dunbar, R. I. M., and Ord, T. J. 2012. Social complexity as a proximate and ultimate factor in communicative complexity. Philosophical Transactions Royal Society B 367:1785-1801.

Freeberg, T. M. 2006. Social Complexity Can Drive Vocal Complexity. Psychological Science 7:557-561.

Hauser, M. D. 1996. The evolution of communication. Cambridge, MA: MIT Press.

Hauser, M. D. 1989. Ontogenetic changes in the comprehension and production of vervet monkey (*Cercopithecus aethiops*) vocalizations. Journal Comparative Psychology 103:149-158.

Husson, F., Josse, J., Le, S. and Maze, J. 2014. Multivariate exploratory data analysis and data mining with R. URL: http://factominer.free.fr

Laporte, M. N. C.; Zuberbühler, K. 2010. Vocal greeting behaviour in wild chimpanzee females. Anim Behav. 80: 467–473.

Long, C. V. 2007. Vocalisations of the degu, *Octodon degus*, a social caviomorph rodent. Bioacoustics 16:223-244.

Manly, B. F. J. 1997. Randomization, Monte Carlo methods in biology chapman and hall. New York.

Mennill, D. J. and Vehrencamp, S. L. 2008. Context-dependent functions of avian duets revealed by microphone-array recordings and multispeaker playback. Current Biology 18:1314-1319.

Monticelli, P. F. and Ades, C. 2013. The rich acoustic repertoire of a precocious rodent, the wild cavy *Cavia aperea*. Bioacoustics 22:49-66.

Monticelli, P. F. 2005. Comportamento e comunicação acústica no preá e na cobaia. Doctoral dissertation, Tese de Doutorado. Instituto de Psicologia, Universidade de São Paulo, São Paulo.

Nogueira, S. S. C.; Caselli, C. B.; Costa, T. S.; Moura, L. N.; Nogueira-Filho, S. L. 2016. The Role of Grunt Calls in the Social Dominance Hierarchy of the White-Lipped Peccary (Mammalia, Tayassuidae). PloS one 11:1-13.

Nogueira, S. S. C.; Pedroza J. P.; Nogueira Filho, S. L. G.; Tokumaru, R. S. 2012. The function of click call emission in capybaras (*Hydrochoerus hydrochaeris*). Ethology, 118: 1-9.

Nogueira Filho, S. L. G and Nogueira, S. S. C. 1999. Criação De Pacas (*Agouti Paca*). 1. Ed. Piracicaba, Sp, Brasil: Fundação de Estudos Agrários - Fealq,. 70p.

Noy Meir, I., Walker, D. and Williams, W. T. 1975. Data transformations in ecological ordination. II. On the meaning of data standardization. Journal of Ecology 63:779-800.

Owren, M. J. and Rendall, D. 2001. Sound on the rebound: bringing form and function back to the forefront in understanding nonhuman primate vocal signaling. Evolutionary Anthropology: Issues, News and Reviews 10:58-71.

Owren, M. J. and Rendal, D.1997. An Affect-conditioning model of nonhuman primate vocal signaling. In: Owings, D. H.; Beecher, M. D.; Thompson, N. S. (EDS.). Perspectives in ethology. Communication. New York, NY: Springer Science Business Media, LLC, v. 12.

Price, T. D.; Qvarnström, A.; Irwin, D. E. 2003. The role of phenotypic plasticity in driving genetic evolution. Proceedings of the Royal Society of London B: Biological Sciences, 270: 1433-1440.

R Core Team. 2014. R: A language and environment for statistical computing. R Foundation for Statistical Computing, Vienna, Austria. URL http://www.R-project.org/.

Reby, D., and McComb, K. 2003a. Anatomical constraints generate honesty: acoustic cues to age and weight in the roars of red deer stags. Animal behavior 65:519-530.

Rendall, D., Owren, M. J., Weerts, E., and Hienz, R. D. 2004. Sex differences in the acoustic structure of vowel-like grunt vocalizations in baboons and their perceptual discrimination by baboon listeners. The Journal of the Acoustical Society of America 115:411-421.

Sabatini, V. and Paranhos Da Costa, M. 2001. Etograma da paca (*Agouti paca*, Linnaeus 1782) em cativeiro. Revista de Etologia 3:3-14.

Schleich, C. E. and Busch, C. 2002. Acoustic signals of a solitary subterranean rodent *Ctenomys talarum* (Rodentia: ctenomyidae): physical characteristics and behavioural correlates. Journal Ethology 20:123-131.

Sih, A. 2013. Understanding variation in behavioural responses to human-induced rapid environmental change: a conceptual overview. Animal Behaviour 85: 1077-1088.

Sih, A.; Ferrari, M. C.; Harris, D. J. 2011. Evolution and behavioural responses to human induced rapid environmental change. Evolutionary Applications, 4: 367-387.

Silk, J. B.; Cheney, D. L.; Seyfarth, R. M. 1996. The form and function of post-conflict interactions between female baboons. Animal Behaviour 52:259-268.

Smythe, N. and Brown de Guanti, O. 1995. La domesticación y cria de la paca (*Agouti paca*). Guia de conservación 26 Roma. FAO, pp. 91.

Smythe, N. 1987. The paca (*Cuniculus paca*) domestic source of protein for the neotropical humid lowlands. Applied Animal behaviour Science 17:155-170.

Sousa-Lima, R. S., Paglia, A. P., & da Fonseca, G. A. 2008. Gender, age, and identity in the isolation calls of Antillean manatees (*Trichechus manatus manatus*). *Aquatic mammals*, 34(1), 109.

Sousa-Lima, R. S., Paglia, A. P., & Da Fonseca, G. A. 2002. Signature information and individual recognition in the isolation calls of Amazonian manatees, *Trichechus inunguis* (Mammalia: Sirenia). *Animal Behaviour*, *63*(2), 301-310.

Suzuki, C. T. 2016. A complexidade do repertório acústico das capivaras (*Hydrochoerus hydrochaeris*). Master's Dissertation, Faculdade de Filosofia, Ciências e Letras de Ribeirão Preto, University of São Paulo, Ribeirão Preto. Retrieved 2016-10-28, from http://www.teses.usp.br/teses/disponiveis/59/59134/tde-15042016-105721/

Topping, M. G.; Miller, J. S.; Goddard, J. A. 1999. The effects of moonlight on nocturnal activity in bushy-tailed wood rats (*Neotoma cinerea*) Ca. J Zool 77:480-485.

Venables, W. N. and Ripley, B. D. 2002. Modern Applied Statistics with S, 4th ed. (Springer, New York), 496 pp.

CAPÍTULO 2

Repertório acústico da cotia vermelha (*Dasyprocta leporina*)

Resumo

A cutia vermelha (*Dasyprocta leporina*) é um roedor caviomorfo territorialista que vive em pares monogâmicos. Seu repertório acústico e os contextos comportamentais de emissão são desconhecidos até o momento. Nesse estudo foi descrito e discutido o repertório vocal de 63 indivíduos cativos em relação ao contexto comportamental de emissão. Como as cotias vermelhas gastam grande parte de seu tempo no forrageio, processando alimentos e são agressivos na defesa territorial contra grupos vizinhos, predizemos que seu repertório vocal apresenta freqüências baixas, com sinais vocais principalmente associados a contextos agressivos durante a alimentação. Além disso, discutimos a complexidade vocal associada à socialidade da espécie. Os resultados corroboram nossa predição e mostram que o repertório acúsico de cotias é composto por 10 tipos de chamados, apresentando baixas freqüências e associados principalmente a contextos agonísticos e de defesa durante o período de alimentação. Nosso estudo também revelou a presença de gradações, transições e combinações de sons que adicionam complexidade ao repertório acústico das cotias vermelhas, como esperado para espécies que vivem em pares ou formam pequenos grupos familiares.

Palavras-chave: Caviomorfo, Bioacústica, Complexidade Vocal, Territorialidade, Comunicação

Introdução

A maioria das informações disponíveis sobre o gênero Dasyprocta diz respeito à cotia da América Central (*Dasyprocta punctata*) (Einsenberg 1974; Smythe, 1978; Francescoli et al., 2016), no entanto, há uma grande escassez de dados sobre a cotia vermelha (*Dasyprocta leporina*), que é amplamente distribuída no continente americano (Emmons e Feer, 1997). Esta espécie ocorre desde o Sul do México até o Norte da Argentina (Eisenberg, 1989) e é distribuída em uma variedade de ecossistemas desde florestas densas até savanas (Eisenberg, 1989). Cotias vivem em pares monogâmicos e podem formar pequenos grupos familiares compostos do par e mais dois ou três filhotes, que juntos se deslocam e defendem seu território contra outros grupos vizinhos (Dubost, 1988). Esses animais apresentam dimorfismo sexual, sendo as fêmeas maiores do que os machos (Dubost, 1988). A espécie é descrita como diurna e sua área de vida é de cinco a dez hectares (Silvius e Fragoso, 2003; Jorge e Perez, 2005), onde os indivíduos usam tocas, troncos de árvores, raízes e vegetação, como refúgio ou descanso (Dubost, 1988). Na natureza, essa espécie passa grande parte do tempo forrageando e processando os alimentos (Santos, 2005). Essa espécie exibe hábito frugívero e mostra comportamento de estocagem (scatter-hoarding), acumulando sementes sob o solo em várias localizações para posterior consumo em tempos de escassez (Dubost, 1988). Tal comportamento de escavar, esconder e buscar sementes tornam as cotias importantes dispersoras de sementes nas florestas (Emmons e Feer, 1997; Santos, 2005). Em relação à comunicação, o repertório acústico de Dasyprocta foi descrito apenas para *D. punctata,* sendo composto por nove tipos de chamados (Einsenberg, 1974; Smythe, 1978). Os sinais acústicos são principalmente produzidos durante três contextos comportamentais – agonístico ou ameaça (*tooth chattering*, *fight grunts*, *growl*, *rumble*), alarme (*alarm bark*, *scream distress*) e contato (*purr, squeak,*

creak squeak) (Einsenberg, 1974; Smythe, 1978). Por outro lado, informações sobre a comunicação de cotia vermelha é basicamente inexistente. (Dubost, 1988; Emmons e Feer, 1997). Até o momento, o único chamado relatado nesta espécie foi o *hollow grunt* ou *bark*, geralmente produzido em contextos de alarme (Dubost, 1988, Emmons e Feer, 1997). Além desta informação, não há mais relatos sobre o repertório desta espécie. Desta forma, o presente estudo tem como objetivo descrever o repertório acústico da cotia vermelha. Além de discutir como os parâmetros acústicos das vocalizações refletem estados motivacionais de acordo com as regras da estrutura motivacional de Morton’s (1977). Esse autor afirma que sons emitidos em contextos hostis são mais ruidosos e possuem freqüências mais baixas (Morton, 1977). Como as cotias vermelhas apresentam comportamento territorialista e gastam a maior parte do seu orçamento temporal na busca e processamento de alimentos, predizemos que seu repertório acústico será composto principalmente por sinais de baixa freqüência, relacionados a contextos agonísticos durante a alimentação.

Material e Métodos

Este trabalho seguiu os princípios de cuidado com animais de laboratório (NIH publicação No. 86-23, revisado em 1985 e foi aprovado pelo comitê de ética e pesquisa animal da Universidade Estadual de Santa Cruz, sob protocolo no. 010/11.

Área de Estudo e Sujeitos

O estudo foi realizado no Centro de Multiplicação de Animais Silvestres (CEMAS) da Universidade Federal Rural do Semi-Árido -UFERSA localizada em Mossoró, estado do Rio Grande do Norte, Brasil. Foram registradas vocalizações e comportamentos de 63 cotias adultas (52 fêmeas e 11 machos) e 10 jovens (oito fêmeas

e dois machos). Todos os indivíduos foram nascidos e criados em cativeiro. Os animais adultos variaram de um a quatro anos de idade e os jovens tinham de 15 dias a cinco meses de idade. Os animais foram alojados em 13 baias reprodutivas e seis baias maternidade. Cada baia reprodutiva alojou grupos de três a 29 indivíduos (Tabela 1). Cada baia maternidade alojou uma mãe com seus respectivos filhotes (um a dois). As baias consistiam de 9 a 12 m² de área, com piso de terra, cercado por paredes de tijolo com 1-m-de altura apoiados por colunas de concreto. O telhado era coberto com telhas e cercado com tela de alambrado (12; 2.5’). Todas as baias continham um tanque de água (0.6m X 0.3m) e dois comedouros (1.0m X 0.3m). Os animais foram alimentados com frutas da estação à vontade, ração para coelho (150 g / animal), sal mineral e água a vontade.

Tabela 1 Número de indivíduos e composição sexual (Macho, Fêmea) de 13 grupos (A-M) de cotias vermelhas utilizadas nesse estudo.

Grupos	A	B	C	D	E	F	G	H	I	J	K	L	M
Número de indivíduos	1♂ 2♀	1♂ 2♀	3♂	1♂ 3♀	1♂ 3♀	4♂	4♀	4♀	1♂ 4♀	1♂ 5♀	13♀	16♀	29♀

Coleta de Dados

Tanto os chamados acústicos, como os comportamentos associados, foram registrados simultaneamente a 1.5 m de distância dos animais. As sessões de observação geralmente ocorreram entre 7:00 e 18:00 horas, período de maior atividade das cotias vermelhas no local estudado. Observamos também os animais em três momentos particulares: durante a limpeza das baias pelo tratador (entre 11:00 e 13:00 horas), durante procedimentos de manejo (entre 7:00 e 9:00 horas) e quando os animais foram alimentados (entre 11:00 e 13:00 horas). Os dados foram registrados *ad libitum*

(Altman, 1974), usando um microfone direcional Sennheiser ME-66 (Wedemark, Germany) e um gravador digital Marantz PMD 670 (Sagamihara, Japan) (configuração do gravador: formato WAV, modo mono, taxa de amostragem 48 kHz, e resolução de 16-bits). O observador iniciou o registro quando os animais estavam ativos e continuou registrando até que a emissão sonora produzida fosse finalizada ou após um intervalo de um minuto sem nenhuma emissão. A coleta de dados totalizou 90 horas de registros e observações. Para estimular a emissão de isolamento ou chamado de contato, quatro mães foram isoladas dos seus filhotes por uma barreira de madeira (122cm X 75cm X 2.5 cm), que impedia o contato físico e visual mas não o contato auditivo. O tratador dos animais foi responsável pela colocação da barreira entre mãe e filhote para evitar estresse dos animais em relação a um novo manejador. O observador iniciou o registro das vocalizações logo após a separação dos animais. Cada par ou trio de mãe/filhotes foram separados apenas uma vez. Essa sessão de registros e observações durou 30 minutos por cada grupo mãe/filhotes.

Análise Acústica

Espectrogramas foram gerados e analisados por meio do Software Raven Pro versão 1.5 (Cornell Lab of Ornithology, Ithaca NY) usando as seguintes configurações: janela tipo Hann, tamanho da janela1350 amostras, 90% de sobreposição no domínio do tempo, e tamanho de DFT 4096. Primeiro os tipos de chamados foram categorizados por inspeção aural e visual dos espectrogramas. A menor unidade vocal dos chamados foi denominada elementos ou notas e foram definidos como um som contínuo sem interrupção (*sensu* Feng et al., 2009 and Barros et al., 2011). Para cada unidade vocal (elementos ou notas) foram medidos os seguintes parâmetros: freqüência mínima (Hz), freqüência máxima (Hz), freqüência dominante (Hz), duração (s), e o intervalo entre elementos (s). Elementos únicos podem ser emitidos repetidamente intercalados com

intervalos de silêncio formando frases (*sensu* Hauser, 1989). Para sons emitidos como frases, foram medidas a duração dos intervalos entre elementos e o ritmo (o número de elementos dividido pela duração total da frase). Foram calculados o intervalo médio entre os elementos e considerado um elemento como parte de outra frase quando o intervalo entre os elementos subsequentes foi maior do que o intervalo médio (*sensu* Barros et al., 2011). Os espectrogramas foram examinados para a presença de não-linearidades e estruturas complexas incluindo gradação, transição e combinação de sons. Para calcular a taxa de emissão por hora dos chamados (taxa horária) foram divididos o número total de cada tipo vocal pelo tempo de observação total (90 h).

Análise estatística

Para testar a validade dos tipos de chamados que foram categorizados pela inspeção aural e visual dos espectrogramas, foi conduzida uma Análise de Função Discriminante (DFA) baseada na medida de cinco parâmetros acústicos - freqüência mínima (Hz), freqüência máxima (Hz), freqüência dominante (Hz), duração (s), e o intervalo entre elementos (s). De 3.263 elementos registrados, foram escolhidos aleatoriamente 50 amostras (elementos) de cada tipo vocal, totalizando 800 amostras. Esse procedimento foi realizado para evitar incluir mais de uma amostra do mesmo elemento da mesma sessão de registro. Todas as vocalizações escolhidas possuem alta qualidade de sinal, menor ruído de fundo e nenhuma sobreposição entre chamados de diferentes indivíduos.

Para determinar a probabilidade de atribuir corretamente cada elemento a um tipo de chamado usamos uma abordagem multivariada (ver Lehner, 1996). A DFA foi conduzida usando um método stepwise e valor F para a aceitação ou rejeição de variáveis independentes que foram fixadas em F = 0,05 e F = 0,01. Além disso, para determinar se foi possível prever corretamente cada tipo vocal com base nos parâmetros

acústicos medidos (variáveis independentes) e usados na DFA, foi realizada a análise de validação cruzada. Posteriormente foi utilizado uma ANOVA, seguida por testes *post hoc* de Tukey (HSD) para testar as diferenças entre as vocalizações discriminadas na análise anterior. Transformações de log (x + 1) foram realizadas para atender a hipótese de normalidade. Logo após foi realizado um teste de normalidade dos parâmetros acústicos após transformação em duas etapas (Templeton, 2011).Todas as análises foram realizadas no software SPSS versão 16, com nível de significância $p < 0.05$.

Resultados

Dezesseis sinais acústicos foram encontrados baseados nas observações diretas e inspeções visuais dos espectrogramas (Figuras 1, 2 e 3). A taxa horária de emissão mostrou que *gemido* (6,7 chamados/s) foi o tipo vocal mais frequente, seguido por *hum-hum* (4,0 chamados/s), *burburinho* (3,8 chamados/s), *có* (3,6 chamados/s), *rangido curto* (3,6 chamados/s), *rangido longo* (3,1 chamados/s), *ronco* (2,5 chamados/s), *rosnado* (2,2 chamados/s), *batida de dente* (1,6 chamados/s), *hu-hu* (1,5 chamados/s), *batida de patas* (1,0 chamados/s), *gru* (0,8 chamados/s), *purr* (0,8 chamados/s), *latido* (0,3 chamados/s), e *choro* (0,05 chamados/s).

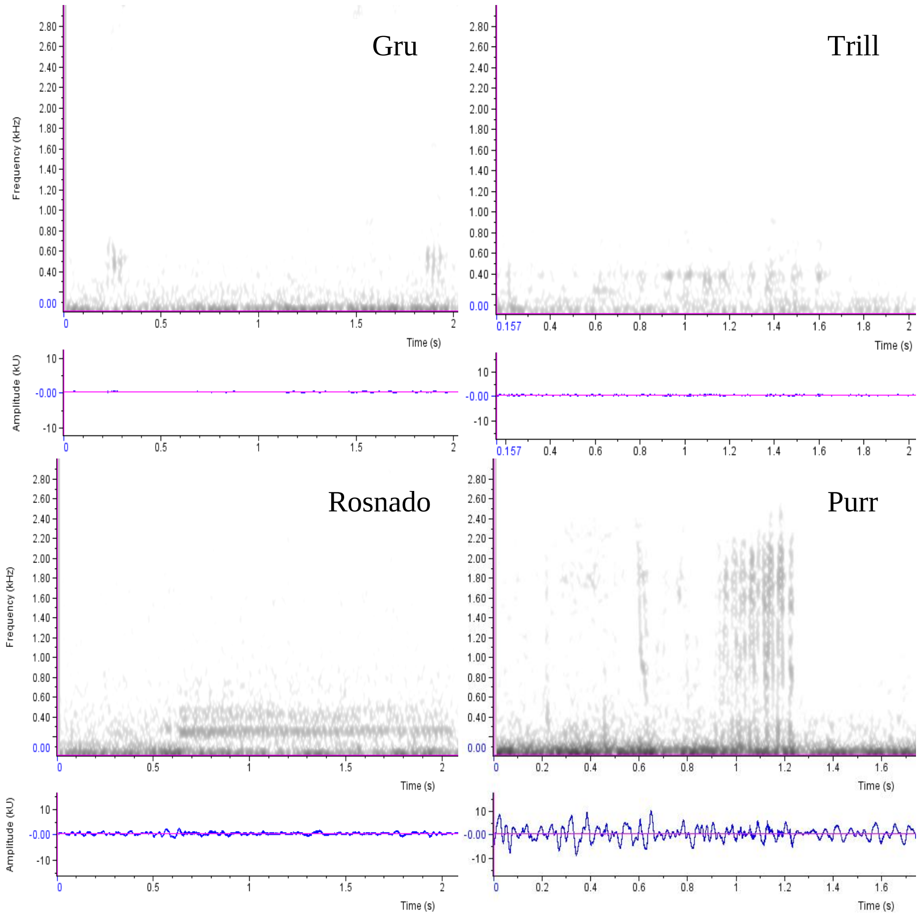

Fig. 1. Espectrogramas e respectivos oscilogramas das vocalizações emitidas por cotias.

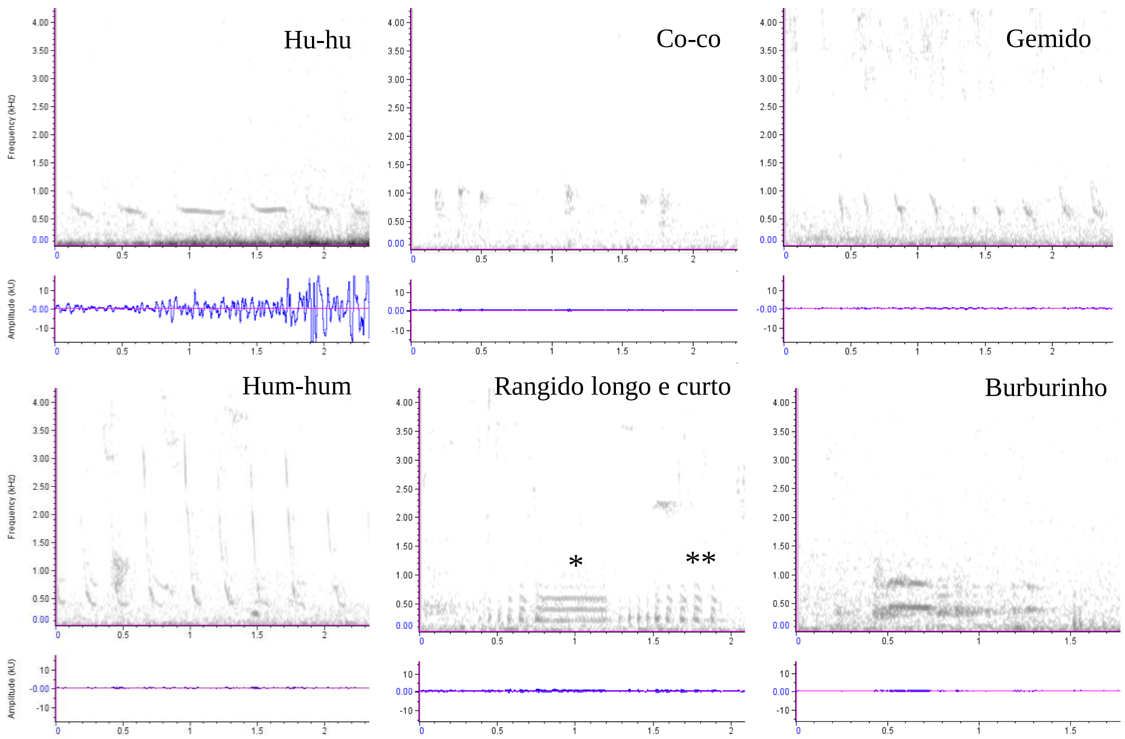

Fig. 2. A estrutura acústica de vocalizações de cotias. Espectrogramas e oscilogramas mostram freqüência e amplitude sobre o tempo. Combinação de elementos *Rangido longo e ** rangido curto, respectivamente.

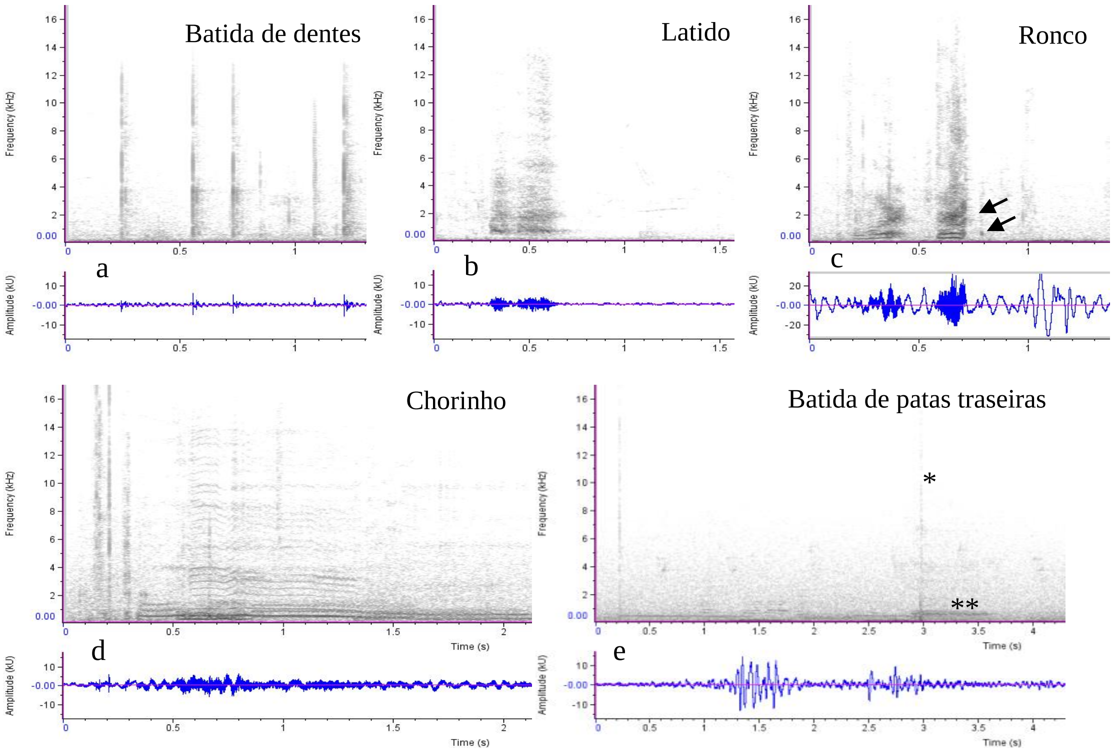

Fig. 3. Espectrogramas e respectivos oscilogramas de sinais acústicos emitidos por cotias: a) sequência de batida de dentes; b) sequência de latido; c) sequência de ronco, setas indicam presença de formantes; d) nota de chorinho; e) combinação de elementos entre *batida de patas e

A DFA, baseada nos 6 parâmetros acústicos medidos: duração das notas (s), frequência máxima (Hz), frequência mínima (Hz), intervalo entre elementos (s), faixa de frequência (Hz) e frequência dominante (Hz), discriminou 10 dos 16 tipos de chamados encontrados nesse estudo (Wilks Lambda= 0,032; P < 0,001; N = 804) (*batida de dente, batida de patas no solo, ronco, purr, rosnado, trill, gemido, hum-hum, rangido longo, rangido curto*) (Figuras 1, 2 e 3 Tabela 2). As duas primeiras funções discriminantes explicaram 80.3% (Tabela 2) da variância entre os chamados. A duração das notas e a frequência máxima foram as variáveis que mais contribuiram para a

primeira função discriminante, enquanto a faixa de frequência contribuiu para a segunda função discriminante.

Tabela 2 Coeficientes das duas principais funções discriminantes (DF) indicando a contribuição de cada parâmetro acústico para distinção entre os tipos vocais. Os valores em negrito indicam os parâmetros com maior peso para a função discriminante (DF1 e DF2).

Parâmetros Acústicos	DF1	DF2
Faixa de Frequência (Hz)	-0.13	**-0.49**
Duração (s)	**0.74**	0.43
Frequencia Max. (Hz)	**-0.51**	0.30
Frequencia Min. (Hz)	-0.07	-0.65
Frequencia Dominante	-0.05	0.07
Intervalo entre elementos (s)	0.03	0.12
Porcentagem da Variância Acumulada	59.0	80.3

A validação cruzada atribuiu corretamente para as categorias vocais com uma precisão de 60.1%, bastante semelhante a classificação original de 62.8%, indicando estabilidade da matriz de dados. No entanto, houve grande variação na porcentagem de atribuição correta das notas entre as várias vocalizações. A acurácia da validação cruzada para as demais categorias vocais variou de 90% (batida de dente) a 21.6% (Gru) (Tabela 3). Dentre as vocalizações que apresentaram menos de 60% das notas atribuídas

corretamente estão: *gru* (21.6%), *hu-hu* (37,3%), latido (37,9%), burburinho (44%) e *có* (58,8%) (Tabela 3).

Tabela 3 Média ± desvio padrão de cada parâmetro acústico medido das vocalizações de cotia vermelha e a porcentagem de notas atribuídas corretamente a cada tipo de chamado na validação cruzada. N corresponde ao número de emissões analisadas em cada categoria.

Tipo Vocal	N	Duração (s)	Frequência Dominante (Hz)	Frequência Min. (Hz)	Frequência Max. (Hz)	Faixa de Frequência (Hz)	Validação Cruzada
Batida de dente	50	0.04 ±0.01	1979.1 ±1936.6	638.9 ±1168.6	20787.7 ±2368.6	7433.4±1605.	90
Batida de pata	50	0.03 ±0.01	823.3 ±330.8	614.5 ±240.9	22714.5 ±1235.7	3940.7±2171.8	86
Ronco	50	0.37 ±0.37	1034.9 ±1050.3	369.3 ±668.8	12645.9 ±8584.31	3828.1±3538.1	60.8
Purr	50	0.09 ±0.07	489.6 ± 369.8	275.3 ± 223.1	4355.6 ± 6700.5	1029.2±1242.7	62.7
Rosnado	50	0.93 ±0.44	320.4 ±127.9	197.1 ±77.4	607.9 ±324.5	239.4±186.1	75
Trill	50	0.27 ±0.19	432.2 ±86.8	259.9 ±65.9	748.5.4 ±296.5	282.2±124.4	71.4
Gemido	50	0.14 ±0.05	701.0 ±223.1	460.0 ±104.8	1067.5 ±297.5	441.4±264.1	72.5
Hum-Hum	50	0.12 ±0.04	414.2 ±156.8	256.0 ±49.8	1137.8 ±648.1	633.7±479.6	80.8
Rangido longo	50	0.43±0.23	264.9±105.2	134.7±29.0	968.3±282.8	352.5±115.9	68.6

Rangido curto	50	0.13±0.19	280.8±139.9	130.7±35.1	946.1±302.4	419.1±139.3	62.7
Gru	50	0.27±0.23	393.1±108.2	234.5±69.1	937.4±205.9	411.5±155.0	21.6
Hu-Hu	50	0.15±0.07	476.8±162.8	318.7±122.3	749.2±298.4	271.6±168.5	37.3
Có	50	0.09±0.04	720.6±361.2	548.6±319.6	897.9±402.2	219.7±106.5	58.8
Burburinho	50	0.79±0.56	377.5±87.2	247.6±75.9	875.5±241.1	359.1±138.0	44
Latido	50	0.12±0.04	1018.3±1909.4	292.9±186.6	15975.3±7993.3	3679.3±2732.3	37.9
Choro	50	1.13±0.43	356.2±110.4	201.3±89.6	11431.4±9331.3	825.0±535.6	60

Na análise de DFA *chorinho, huhu, gru, latido* e *burburinho* foram confundidos com *burburinho, có, hum hum, purr* e *rosnado* respectivamente, possivelmente devido a similaridade estrutural resultante de gradação e transição ente eles (Tabela 3; Figuras 1, 2 e 3). Além disso, encontramos combinações entre os sinais acústicos: *rangido longo* e *rangido curto* (Figura 2) e entre *batida de pata* e *rosnado* (Figura 3).

As vocalizações diferiram significativamente em todos os parâmetros acústicos estudados (Tabela 4). Os testes dois a dois (Tukey B) mostraram que cada par de vocalizações apresentou diferença significativa em pelo menos um parâmetro acústico (Tabela 5).

Tabela 4. Médias e valores de P dos parâmetros acústicos utilizados na Análise de variância (ANOVA). Efeito significativo destacado em negrito.

		Soma dos quadrados	df	Quadrado médio	F	p
Freq. min.	Inter grupos	29,201	15	1,947	43,998	**,000**
	Intra grupos	32,565	736	,044		
	Total	61,765	751			
Freq. max.	Inter grupos	42,978	15	2,865	107,551	**,000**
	Intra grupos	19,634	737	,027		
	Total	62,612	752			
Faixa de freq.	Inter grupos	39,488	15	2,633	88,581	**,000**
	Intra grupos	21,873	736	,030		
	Total	61,361	751			
Duração	Inter grupos	45,113	15	3,008	133,731	**,000**
	Intra grupos	16,552	736	,022		
	Total	61,666	751			
Freq. fundamenta l	Inter grupos	24,747	15	1,650	32,943	**,000**
	Intra grupos	36,859	736	,050		
	Total	61,606	751			
Intervalo entre	Inter grupos	13,534	15	,902	20,997	**,000**
	Intra grupos	31,670	737	,043		

elementos	Total		45,205	752

Tabela 5 Diferenças significativas entre tipos vocais de cotias vermelhas (testes post hoc de Tukey p<0.05). Os numerous correspondem a 1 –freq. min., 2 – freq. max., 3 – faixa de freq., 4 – duração, 5 – freq. fundamental, 6 – intervalo entre notas. Abreviações: bd (batida de dente), ge (gemido), hm (hum-hum), bu (burburinho), có (có), rc (rangido curto, rl (rangido longo), ro (ronco), rs (rosnado), hu (hu-hu) bp (batida de patas), gr (gru), pu (purr), lat (latido) e ch (choro).

	tri	bp	rs	pu	lat	gr	bu	rc	rl	hm	ge	có	hu	ch	ro
bd	2,3,4,5,6	1,3,4,6	1,2,3,4,5,6	1,2,3,4	1,3,4,5	1,2,3,4,5	1,2,3,4,5	1,2,3,4,5	1,2,3,4,5	2,3,4,5	2,3,4	2,3,4	2,3,4,5	1,3,4,5	1,3,4,5
ro	2,3,6	1,2,4,5,6	2,3,4,5	2,3,4,6	4,6	2,3	2,3,4	1,2,3,4,5,6	1,2,3,5	2,3,4	1,2,3,4,5	1,2,3,4,5,6	1,2,3,4,6	3,4,5	
ch	2,3,4,6	1,2,3,4,6	2,3,6	4	4	2,3,4	2,3,4	1,2,3,4	1,2,3,4	2,4	1,2,3,4	1,2,3,4	1,2,3,4		
hu	4,6	1,2,3,4,5,6	1,2,4,5,6	2,3,4	2,3	2,3	2,3,4	1,2,3,5	1,2,3,4,5	2,3	1,2,3,5	1,4,5			
co	1,4,5,6	2,3,4,6	1,2,4,5,6	1,2,3	1,2,3,5	1,3,4,5	1,3,4,5	1,3,5	1,3,4,5	1,3,5	2,3,4				
ge	1,2,3,4,5,6	2,3,4,6	1,2,3,4,5,6	1,2,3,4	1,2,3,5	1,5	1,4,5	1,4,5	1,4,5	1,5					
hm	2,3,4,6	1,2,3,4,5	2,3,4,6	2,4	2,3,6	4	3,4	1,5	1,3,4,5						

		,6						
r l	1,2,4,5,6	1,2,3,4,5,6	1,2,3,4,6	1,2,3,4,5	1,2,3,4,5	1,4,5	1,4,5	4
rc	1,2,3,4,5	1,2,3,4,5,6	1,2,3,4,6	1,2,3,5	1,2,3,5	1,4,5	1,4,5	
bu	2,4,6	1,2,3,4,5,6	2,3,4,6	3,4	2,3,4	4		
gr	2,3,6	1,2,3,4,5,6	2,3,4,6	2,3,4,6	2,3,4,6			
la	2,3,4	1,4,5,6	2,3,4,5,6	2,3,4				
pu	2,3,4	1,2,3,4,6	2,3,4,6					
rs	2,4,5,6	1,2,3,4,5						
bp	1,2,3,,4,5,6							

A maioria das vocalizações de cotias apresentam baixa frequência (Tabela 3). As observações comportamentais associadas às emissões acústicas produzidas, permitiram analisar que 75% dos chamados nas cotias foram emitidos em contextos agonísticos e de ameaça (*chorinho, gru, trill, rosnado, hu-hu, có, gemido, batida de dentes, latido, batida de patas traseiras e ronco*), enquanto 12,5% em situação de contato (*purr, hum-hum, burburinho*) e 12,5% em contexto de distress/alarme (*rangido longo, rangido curto, latido*) (Tabela 6).

Tabela 6 Descrição do contexto comportamental e possível função comunicativa dos sons de cotias associadas a idade (A: adultos e J: jovens) e sexo (M: macho e F: fêmea) do emissor.

Chamado	Função comunicativa	Categoria idade e sexo	Contexto
Batida de dente	Agonístico	A, M, F	Sinal mecânico produzido pelo entrechoque de incisivos inferiores e superiores, emitido em sequências de três a doze elementos. O animal assume postura de alerta e produz esses sons durante isolamento mãe/filhote, presença de humanos não familiares, encontros agonísticos e durante disputas por alimentos entre coespecíficos.
Batida de patas	Agonístico	A, J,M, F	Sinal mecânico produzido pela batida das patas inferiores no solo. São pulsos tonais realizados em sequências de quatro a nove elementos. São emitidos em contexto de isolamento mãe/filhote e durante a presença de pessoas não familiares. Quando a mãe faz esse movimento o filhote começa a imitar. Esses sons também são produzidos em associação com o tipo vocal *rosnado*.
Ronco	Agonístico	A, M, F	São chamados ruidosos,, com presença de formantes e até quatro harmônicos nem sempre visíveis. Pode ser emitido como um único elemento ou em sequências de dois a quatro elementos. São produzidos quando os animais estão próximos um do outro, durante encontros agressivos por disputa de alimentos. Esse chamado é quase sempre seguido por tentativa de mordidas e perseguição por parte do emissor.
Rosnado	Agonístico	A, M, F	São chamados de frequência baixa com estrutura harmônica de até dois harmônicos. São emitidos durante encontros agressivos, por disputa de alimentos que envolvem lutas, durante captura para manejo, em que o animal vocaliza para o tratador e também durante isolamento mãe/filhote. Os animais assumem posstura de alerta e eriça os pêlos.

Trill	Agonístico	A, J, F	São vocalizações de freqüência baixa, emitidas em sequências de quatro a 11 elementos, durante aproximação de pessoas não familiares e durante isolamento mãe/filhote.
Gemido	Agonístico	A, M, F	São vocalizações tonais de frequência baixa emitidas em sequências de dois a 21 elementos, emitidas durante disputa por alimentos, em que um animal persegue o outro durante alimentação.
Có	Agonístico	A,M,F	São vocalizações tonais de baixa frequencia, emitidas como único elemento ou em sequências de dois a três elementos. Foram emitidas durante disputas por alimento, em que um animal persegue o outro para pegar o seu alimento, porém não ocorre agressão física entre os animais.
Gru	Agonístico	A,F,M,F	São vocalizações de baixa frequência emitidos em sequências de três a quatro elementos. Foram emitidos para o tratador durante captura para manejo, e disputa por alimentos seguidos por posturas de alerta e eriçamento dos pêlos. O animal também pode realizar a *batida de patas traseiras no solo* e vocalizar o *gru*. Parece ter função de ameaça.
Huhu	Agonístico	A,M,F	São vocalizações tonais de baixa frequência, emitidas como um único elemento ou em sequências de dois a 12 elementos. Foram emitidas durante disputas por alimento, em que um animal persegue o outro para pegar o seu alimento, porém não ocorre agressão física entre os animais.
Burburinho	Agonístico	A,J,M,F	São vocalizações tonais de baixa freqüência, com estrutura harmônica espaçada contendo de um a três harmônicos visíveis. São emitidas pelas mães e pelos filhotes durante aproximação de pessoas não familiares.
Chorinho	Agonístico	A,F	Vocalização tonal de alta freqüência, com presença de estrutura harmônica. Produzida durante disputa por alimentos. Emitida pelo animal que perde a disputa.
Latido	Agonístico/ Alarme	A,F,M,F	São vocalizações emitidas como único elemento ou em sequências de dois a quatro elementos. Foram emitidos por adultos e filhotes durante captura pela rede de manejo, durante presença de pessoas e barulhos/ruídos não familiares. Geralmente o animal apresenta postura de alerta.
Rangido longo	Distress/	A,M,F	São vocalizações tonais de baixa freqüência e com estrutura harmônica de três a quatro harmônicos visíveis, emitidas em sequências de até quatro elementos. Pode ser emitido sozinho ou combinado com o *rangido curto*, quando o animal foi injuriado durante encontros

	Alarme		agonísticos e durante captura com a rede. Esse chamado possivelmente expressa dor ou serve para alertar os coespecíficos.
Rangido curto	Distress/<br>Alarme	A,M,F	São vocalizações tonais de curta duração e frequência baixa, com estrutura harmônica de 3três a seis harmônicos visíveis, emitidas em sequências de quatro a 12 notas. Podem ser emitidos sozinhos ou em combinação com o *rangido longo*. Foram emitidos quando o animal foi injuriado durante encontros agonísticos e durante captura com a rede para procedimentos de manejo. Esse chamado possivelmente expressa dor ou serve para alertar os coespecíficos sobre experiências negativas.
Hum-Hum	Contato	A, M,F	São chamados tonais de baixa frequência com até dois harmônicos visíveis, emitidos em sequências de nove a 32 elementos. Emitidos apenas durante o fornecimento da alimentação. Neste contexto os animais ficam muito agitados, vocalizam e caminham explorando o recinto.
Purr	Contato	A, J, F	São pulsos curtos, emitidos como um único elemento ou em sequências de dois a dez elementos. São emitidos por filhotes e fêmeas adultas durante separação mãe/filhote.

Durante a separação mãe/filhote encontramos uma emissão vocal exclusivamente emitida pelos filhotes e por suas mães - o *purr* - sugerindo um chamado de contato (Tabela 4). Durante as emissões de *purr* pelos filhotes, a mãe permaneceu bastante agitada, movendo-se pelo recinto e tentando derrubar a barreira de madeira entre eles, produzindo *purr, batidas de dente, trill, rosnados* e *batidas de pata traseira no solo* (thumping). Além do *purr*, os filhotes também emitiram *trill* e *batidas de pata traseira*.

Discussão

O repertório acústico da cotia vermelha é composto por 10 sinais (8 tipos vocais e dois sinais mecânicos). Como hipotetizamos, a maioria dos sinais possui baixas frequências e são produzidos pela espécie em contextos agonísticos durante a alimentação possivelmente associados a comportamento de defesa. Nossos resultados também confirmam nossa hipótese de que há diferença entre os chamados de machos e fêmeas e que, portanto, pode haver reconhecimento entre os sexos. Além disso, encontramos a presença de estruturas complexas como gradação, transição e combinação de sons que são esperados em espécies mais sociais.

Os sinais acústicos da cotia vermelha descritos nesse estudo são inéditos e, portanto, não puderam ser comparados com outros estudos da mesma espécie. No entanto, nossos achados se assemelham as descrições acústicas feitas por Eisenberg (1974) e Smythe (1978) para outra espécie de cotia a *Dasyprocta puntacta*. Estes autores encontraram nove tipos de sinais em animais de vida livre, sendo dois mecânicos e sete vocalizações, a maior parte emitida em contextos agonísticos, como

encontrada neste estudo. Esta descrição do repertório, porém, não inclui os parâmetros acústicos dos chamados ou imagens, o que impede maiores interpretações e comparações.

Os contextos associados às emissões do repertório acústico de cotias vermelhas encontrados no presente estudo são compatíveis com o esperado para uma espécie com comportamento agressivo na defesa do território e de recursos. Um estudo realizado para descrever os comportamentos da cotia vermelha em cativeiro, encontrou a maior parte das interações agonísticas durante a alimentação (Kaiser et al., 2011) e confirma o que encontramos em nosso estudo. Smythe (1978) destaca que a cotia da América central também apresenta maior agressividade na defesa territórial nos períodos de escassez de alimentos.

Como esperado encontramos a predominância de freqüências baixas (menor que 1979.1 Hz) no repertório acústico da cotia vermelha. Esse resultado está de acordo com as regras de estrutura motivacional de Morton (1977), que relata que sons emitidos em contextos hostis geralmente possuem freqüências baixas e são ruidosos. Essas características acústicas também foram encontradas na paca (*Cuniculus paca*) um roedor caviomorfo territorialista (Eisenberg, 1974, capítulo um desta tese) em que os sons de frequência baixa possivelmente promovem defesa territorial e encontro com parceiros, pois podem ser transmitidos a longas distâncias (Bradbury e Vehremcamp, 1998). Este mesmo efeito parece ocorrer com a cotia vermelha o que reforça a nossa predição sobre a agressividade relacionada a defesa territorial nessa espécie.

Encontramos seis tipos de sinais acústicos emitidos em contextos agressivos no repertório da cotia vermelha. A *batida de patas no solo* emitido por cotias, também foi descrito para mocós (*tamborilar:* Alencar Jr., 2012) e foi relatado na paca (batida de pata: capítulo um desta tese) e na cotia da América central (*D. punctata:* Smythe, 1978),

sendo emitidos em contextos semelhantes aos encontrados nesse estudo. As *batidas de dente* apresentam a mesma estrutura acústica encontrada em outros roedores caviomorfos como a paca (*Cuniculus paca:* capítulo um desta tese), capivara (*Hydrochoerus hydrochaerys:* Barros et al., 2011), preá (*Cavia aperea:* Monticelli e Ades, 2013), cobaia (*Cavia porcelus:* Monticelli, 2005), mocó (*Kerodon rupestris:* Alencar Jr., 2012), chinchila (*Chinchilla lanígera:* Bartl, 2006) e coruro (*Spalacopus cyanus:*Veitl, 2000). O *ronco* foi observado durante disputa por alimentos e possivelmente envolve comportamento de defesa territorial. Essa mesma função de defesa foi atribuída a três espécies de roedores caviomorfos com padrões acústicos similares: a paca (*growl/bark,* capítulo um desta tese), o degu (*Octodon degus, grunt:* Long, 2007) e o mocó (*grunt:*Alencar Jr., 2012). Nos roncos observamos a presença de formantes, que são estruturas que geralmente refletem o tamanho do corpo e a anatomia do trato vocal dos animais, permitindo reconhecimento individual e acesso a características como idade e sexo (Owren e Rendall, 2001; Reby e McComb, 2003a; Rendall et al., 2004). As vocalizações *rosnado, trill* e *gemido* da cotia vermelha também são emitidas por outros roedores caviomorfos e apresentam as mesmas funções agonísticas relatadas aqui, como o *cackle* de capivara (Barros et al., 2011; Suzuki, 2016), o *whine* e *groan* de degus (Long, 2007). Essa semelhança contextual mostra similaridades filogenéticas neste táxon (Eisenberg, 1974).

Encontramos dois tipos de vocalizações com possível função de contato: *hum-hum* e *purr. Hum-Hum* foi emitido durante o fornecimento da alimentação, possivelmente na expectativa de receber o alimento, causando excitação nos animais. *Como a alimentação era fornecida sempre nos mesmos horários,.* esse comportamento parece estar relacionado ao condicionamento e pode ser explicado porque os animais fazem associação entre a liberação do alimento e os sinais produzidos pelo tratador,

como barulhos de passos, vozes, barulhos da preparação dos alimentos. Outros roedores caviomorfos como pacas (*click*: capítulo um desta tese), preás (*whistle*: Ades et al., 1994) e capivaras *(click:* Barros et al. (2011), também apresentam este mesmo comportamento observado aqui. *Purr* foram produzidos por mães e filhotes durante a separação física, provavelmente para reestabelecer contato entre eles. Chamados de isolamento utilizados nesse mesmo contexto são emitidos por pacas (*cry*: capítulo um desta tese), preás (*isolation whistle*: Monticelli e Ades, 2013), por degus *(loud whistle:* Long, 2007), capivaras *(whistle:* Dos Santos et al., 2014); e tuco-tuco *(Cry, Ctenomys talarum*: Schleich e Busch, 2002), e reforçam as similaridades funcionais em caviomorfos.

Nesse estudo, dois tipos de vocalizações foram emitidos por cotias vermelhas em contextos de alarme: o rangido longo e o rangido curto. Ambos chamados foram produzidos pelos animais ao sofrer injúrias durante a contenção para procedimentos de manejo, possivelmente para expressar dor ou alertar os coespecíficos sobre estímulos negativos. Chamados de alarme nesse contexto são comuns em caviomorfos (Eisenberg, 1974) e são encontrados em g*roans* de pacas (capítulo um desta tese), *groans* de degus (*Octodon degu*: Long, 2007), *nasal hiss* mocós (*Kerodon rupestris*: Alencar Jr., 2012), *whine* em cobaias (*Cavia porcellus*: Monticelli e Ades, 2013) e *squeal* de capivaras. Essas semelhanças funcionais e estruturais encontradas nos repertorios de caviomorfos sugerem que futuros estudos possam utilizar comportamento acústico como caracteres em análises filogenéticas.

O presente estudo revelou a presença de gradação, transição e combinação de sons entre os sinais acústicos da cotia vermelha, revelando a complexidade do repertório acústico dessa espécie. Os repertórios acústicos podem ser classificados como discretos, graduados ou uma mistura dos dois (Hauser, 1996; Ranger e Fischer, 2004).

Sistemas acústicos contínuos incluem tipos de chamados que apresenta uma transição gradual de uma estrutura a outra sem um limite acústico claro e que podem ser emitidos em diferentes contextos (Hauser, 1996). Os fenômenos de gradação e transição entre os sinais da cotia vermelha aqui descritos, podem ter impedido a distinção entre os tipos vocais *chorinho/burburinho, huhu /có, gru/hum hum, latido*/*purr* e *burburinho*/*rosnado* pela DFA e na análise de validação cruzada. A presença de gradação e transição é uma característica comum nos repertórios de histricognatos e parecem comunicar diferentes estados motivacionais do emissor (Einsenberg 1974). Este tipo de repertório contínuo dificulta a tarefa de classificar vocalizações biologicamente significativas em tipos distintos de chamados (Keenan et al., 2013). No presente estudo, também encontramos combinações de sons entre *rangido longo* e *rangido curto* e entre *batida de pata* e *rosnado*. Devido a restrições anatômicas, o número de diferentes vocalizações com estrutura acústica discreta que um animal pode produzir é limitado (Arnold e Zuberbühler, 2006). Uma maneira de superar essas limitações é combinar diferentes elementos em sequências (Manser et al., 2014). A combinação de sons, assim como transição e gradação tem o potencial de aumentar a variabilidade dos chamados (Hauser, 1996), e possivelmente aumentar a complexidade acústica do repertório acústico da cotia vermelha.

Conclusão

O presente estudo descreveu o repertório da cotia vermelha composto por 10 tipos de sinais acústicos produzidos em sua maior parte em contextos agressivos durante a alimentação, possivelmente relacionado a sua defesa territorial e disponibilidade de alimentos. A complexidade encontrada no repertório acústico através de gradação,

transição e combinações de sons está em concordância com a característica mais social da espécie.

Referências

Ades, C., Tokumaru, R. S.; Beisiegel, B. M. 1994. Vocalizações antecipatórias da cobaia *Cavia porcellus* em situação de alimentação. Biotemas 7:79-93.

Alencar, R. N. Jr. 2012. O repertório acústico de um especialista de rochedos da caatinga, o mocó [dissertação de mestrado]. São Paulo: Departamento de Psicologia Experimental, Universidade de São Paulo.

Altmann, J. 1974. Observational study of behaviour: sampling methods. Behaviour 49: 223—265.

Arnold, K., & Zuberbühler, K. 2006. Language evolution: semantic combinations in primate calls. Nature 441: 303-303.

Barros, K. S., Tokumaru, R. S., Pedroza, J. P. & Nogueira, S. S. C. 2011. Vocal Repertoire of Captive Capybara (*Hydrochoerus hydrochaeris*): structure, context and function. Ethology 116: 83–93.

Bartl, J. 2006. Lautäußerungen der Chinchillas im Sozialverband (Doctoral dissertation, lmu).

Bradbury, J. W & Vehrencamp, S. L. 1998. Principles of Animal Communication. Sinauer Associates, Canada.

Dos Santos, E.; Tokumaru, R. S.; Nogueira Filho, S. L. G.; Nogueira, S. S. C. 2014. The effects of unrelated offspring whistle calls on capybaras (*Hydrochoerus hydrochaeris*). Braz. J. Biol. 74: S171-S176.

Dubost, G. 1988. Ecology and social life of the red acouchi, Myoprocta exilis; acomparison with the orange-rumped agouti, *Dasyprocta leporine*. J. Zool. Lond. 214: 107-123.

Eisenberg, J. F. 1974. The function and motivational basis of hystricomorph vocalizations. Symp. Zool. Soc. Lond. 34: 211-247.

Eisenberg, J. F. 1989. Mammals of the Neotropics. v. 1. The northern neotropics: Panama, Colombia, Venezuela, Guyana, Suriname, French Guiana.

Emmons, L. H., & Feer, F. 1997. Neotropical rainforest mammals. A field guide, 2.

Feng, A. S., Riede, T., Arch, V. S., Yu, Z., Xu, Z. M., Yu, X. J. & Shen, J. X. 2009. Diversity of the vocal signals of concave-eared torrent frogs (*Odorrana tormota*): evidence for individual signatures. Ethology 115: 1015—1028.

Francescoli, G.; Nogueira, S. and Schleich, C. 2016. Sociobiology of caviomorph rodents: an integrative view Chapter 6: Mechanisms of social communication in caviomorph rodents.

Hauser, M. D. 1996. The evolution of communication. Cambridge, MA: MIT Press.

Hauser, M. D. 1989. Ontogenetic changes in the comprehension and production of Vervet Monkey (*Cercopithecus aethiops*) vocalizations. J. Comp. Psychol. 103: 149—158.

Jorge, M. S., & Peres, C. A. 2005. Population Density and Home Range Size of Red- Rumped Agoutis (*Dasyprocta leporina*) Within and Outside a Natural Brazil Nut Stand in Southeastern Amazonia1. Biotropica 37: 317-321.

Kaiser, S. K., Margarido, T. C. C., & Fischer, M. L. 2011. Avaliação do comportamento de cutias Dasyprocta azarae e Dasyprocta leporina (Rodentia: Dasyproctidae) em cativeiro e semicativeiro em parques urbanos de Curitiba, Paraná, Brasil. Revista de Etologia 10: 68-82.

Keenan, S., Lemasson, A., & Zuberbu¨ hler, K. 2013. Graded or discrete? A quantitative analysis of Campbell's monkey alarm calls. Animal Behaviour 85: 109–118.

Lehner, P. N. 1996. Handbook of Ethological Methods. Cambridge Univ. Press, Cambridge, p. 672.

Long, C. V. 2007. Vocalisations of the degu *Octodon degus*, a social caviomorph rodent. Bioacoustics 16: 223-244.

Manser, M. B., Jansen, D. A.,Graw, B., Hollén, L. I., Bousquet, C. A., Furrer, R. D., & le Roux, A. 2014. Vocal complexity in meerkats and other mongoose species. Advances in the Study of Behavior, 46, 281.

Monticelli, P. F. e Ades, C. 2013. The rich acoustic repertoire of a precocious rodent, the wild cavy *Cavia aperea* , Bioacoustics: The International Journal of Animal Sound and its Recording, 22:1, 49-66

Monticelli, P. F. 2005. Comportamento e comunicação acústica em cobaias e preás. Departamento de Psicologia Experimental. São Paulo, Universidade de São Paulo: 161.

Morton, E. S. 1977. On the occurrence and significance of motivational-structural rules in some bird and mammal sounds. Am. Nat. 111: 855–869.

Owren, M. J. & Rendall, D. 2001. Sound on the rebound: bringing form and function back to the forefront in understanding nonhuman primate vocal signaling. Evolutionary Anthropology: Issues, News, and Reviews 10: 58-71.

Range, F. N., & Fischer, J. 2004. Vocal repertoire of sooty mangabeys (*Cercocebus torquatusatys*) in the Taı¨ National Park. Ethology 110: 301–321.

Reby, D., & McComb, K. 2003. Anatomical constraints generate honesty: acoustic cues to age and weight in the roars of red deer stags. Animal behaviour 65: 519-530.

Rendall, D., Owren, M. J., Weerts, E., & Hienz, R. D. 2004. Sex differences in the acoustic structure of vowel-like grunt vocalizations in baboons and their perceptual discrimination by baboon listeners. The Journal of the Acoustical Society of America 115: 411-421.

Santos, E. F. 2005. Ecologia da cutia Dasyprocta leporina (Linnaeus, 1758) em um fragmento florestal urbano em Campinas-SP (Rodentia: Dasyproctidae).

Schleich, C. E. & Busch, C. 2002. Acoustic signals of a solitary subterranean rodent *Ctenomys talarum* (Rodentia: ctenomyidae): physical characteristics and behavioural correlates. J. Ethol. 20: 123-131.

Silvius, K. M., & Fragoso, J. 2003. Red rumped Agouti (*Dasyprocta leporina*) Home Range Use in an Amazonian Forest: Implications for the Aggregated Distribution of Forest Trees. Biotropica 35: 74-83.

Smythe, N. 1978. The natural history of the Central American agouti (*Dasyprocta punctata*). Smithsonian Contributions to Zoology 257: 1-52.57

Suzuki, C. T. 2016. A complexidade do repertório acústico das capivaras *(Hydrochoerus hydrochaeris)*. Master's Dissertation, Faculdade de Filosofia, Ciências e Letras de Ribeirão Preto, University of São Paulo, Ribeirão Preto. Retrieved 2016-10-28, from http://www.teses.usp.br/teses/disponiveis/59/59134/tde-15042016-105721/

Templeton, G. F. 2011. A two-step approach for transforming continuous variables to normal: implications and recommendations for IS research. Communications of the Association for Information Systems 28: 41-58.

Veitl, S., Begall, S., & Burda, H. 2000. Ecological determinants of vocalisation parameters: the case of the coruro *Spalacopus cyanus* (Octodontidae), a fossorial social rodent. Bioacoustics 11: 129-148.

CAPÍTULO 3

Complexidade vocal e social em Caviomorfos

Resumo

A hipótese da complexidade social para comunicação afirma que espécies mais sociais, apresentam repertórios acústicos mais amplos e diversificados quando comparados a espécies solitárias ou menos sociais. No entanto, outros fatores não sociais (ex: reprodutivos e ecológicos) também podem atuar isoladamente ou em conjunto com fatores sociais aumentando a complexidade vocal. Assim, o objetivo deste estudo foi analisar se há correlação entre fatores que envolvem diferentes tipos sociais, reprodutivos e ecológicos com a complexidade vocal em roedores caviomorfos. Dez espécies deste grupo foram escolhidas para compor as análises por terem dados disponíveis na literatura sobre sua composição social, seu repertório acústico, reprodução e ecologia. Os resultados confirmaram que quanto mais complexo o tipo de sistema social da espécie, maior seu repertório vocal ($P = 0{,}02$). Porém, tal relação positiva existe apenas para o repertório amigável de sons que aumentou linearmente com o número de machos em relação às fêmeas no grupo (composição do grupo); o número de filhotes na ninhada (tamanho da prole) ($P < 0{,}01$); a variação de solitário a harém (sistema social) ($P < 0{,}01$) e a variação de comportamento monogâmico à promíscuo (sistema de acasalamento) ($P < 0{,}01$).

Palavras-chave: Bioacústica, Comunicação animal, Comportamento social, Repertório acústico, Roedores, Socialidade.

Introdução

Características ecológicas e/ou sociais podem influenciar a complexidade vocal nos mamíferos. Há evidências de que predação, características ambientais e fatores de ordem reprodutiva, como a seleção sexual, podem levar ao aumento na complexidade acústica das espécies (Freeberg et al., 2012; Ord e Garcia-Porta, 2012). A pressão por predação, por exemplo, pode levar ao aumento no tamanho do repertório de alarme em ambientes com muitos tipos de predadores (Furrer e Manser, 2009). Os fatores ambientais, por sua vez, podem afetar a fidelidade do sinal (Freeberg et al., 2012) ou favorecer o uso de sinais acústicos no lugar de sinais visuais em ambientes densamente florestados (Bradbury e Vehrencamp, 2011). Já a seleção sexual pode atuar na complexidade estrutural da vocalização, quando características específicas na estrutura do chamado de machos são mais atrativas para as fêmeas (Bernal et al., 2009). Neste contexto, a hipótese da complexidade social afirma que quanto mais social for a espécie, ou seja, maior o número de interações entre os coespecíficos, mais amplo e diversificado será seu repertório acústico em comparação com as espécies menos sociais ou solitárias, que raramente interagem ou quando interagem geralmente o fazem na estação reprodutiva, além de pouco ou nunca repetirem tal interação com o mesmo indivíduo (McComb e Semple, 2005; Freeberg et al., 2012).

O tema foi explorado por Freeberg (2012) em uma revisão que conceituou a complexidade social como: "*..a complexidade em sistemas sociais está relacionada ao número de indivíduos interagindo, aos diferentes tipos ou papéis sociais desses indivíduos e a natureza e diversidade de interações entre eles."* Deste modo, para descrever a complexidade social de uma dada espécie, algumas características são essenciais, tal como o tipo de sistema social (Bouchet et al., 2013), o tipo de sistema de

acasalamento (Stirling e Thomas, 2003; Devillard et al., 2004) e o tamanho do grupo social (McComb e Semple, 2005; Freeberg, 2006). O tamanho do grupo social é a característica social mais estudada em uma variedade de taxa (Pollard e Blumstein, 2012), que apresentam evidencias da existência de correlação entre o tamanho do repertório vocal e o tamanho do grupo social (Blumstein e Armitage, 1997; McComb e Semple, 2005; Freeberg, 2006; Freeberg e Harvey 2008; Freeberg et al., 2012). Entretanto, outros estudos comparativos (*Heliophobius argenteocinereus*, *Fukomys mechowii*, *Fukomys darlingi*: Hrouzkova, 2012; *Suricata suricatta, Mungos mungo, Helogale parvula, Cynictis penicillata, Galerella sanguinea*: Manser et al., 2014) tem encontrado que tamanho de grupo apenas, não é bom preditor de tamanho de repertório vocal. Nesse caso, dividir o repertório entre contextos específicos da produção do chamado, como durante interações agonísticas, afiliativas e de alarme auxilia para uma melhor compreensão da complexiade vocal (Manser et al., 2014).

Com relação à complexidade vocal, esta pode ser acessada pelo menos por quatro tipos de características - tamanho do repertório acústico geral da espécie (Blumstein e Armitage, 1997; McComb e Semple, 2005), tamanhodo repertório com funções mais específicas (ex: diversidade de chamados amigáveis para manutenção da coesão do grupo) (Morton, 1977), presença de informações como idade, sexo, tamanho ou identidade do emissor em assinaturas vocais (Pollard e Blumstein, 2011) ou pela presença de características na estrutura acústica das vocalizações tais como gradações, transições e combinações de notas ou elementos que compõem tais vocalizações (Wilson, 2000; Crockford e Boesch, 2005). Estas características podem favorecer o aumento na variabilidade acústica desses repertórios e auxiliar para uma comunicação mais eficiente entre os indivíduos de um grupo social (Wilson, 2000; Crockford e Boesch, 2005).

A relação existente entre a complexidade social e a comunicação tem sido examinada em várias espécies e sugere que diferentes atributos da complexidade social possivelmente conduzem a uma complexidade vocal (Pollard e Blumstein, 2012). Em roedores, por exemplo, um estudo com 22 espécies da família Sciuridae relatou que o tamanho do repertório de alarme aumenta de acordo com a complexidade dos papéis demográficos (composição idade/sexo) do grupo social (Blumstein e Armitage, 1997; Pollard e Blumstein, 2012). Em primatas não humanos, 40 espécies foram investigadas e os autores concluíram que o tamanho do repertório vocal está relacionado ao grau dos laços sociais exibidos pelos indivíduos dessas espécies (Mccomb e Semple, 2005). Nas aves, um estudo com *Poecile carolinenses* revelou que o tamanho do repertório vocal e a complexidade dos chamados *chick-a-dee* aumentam com o tamanho do grupo (Freeberg, 2006). Apesar do conhecimento de alguns fatores que pressionam na direção de uma complexidade vocal, ainda há muito para compreender sobre quais fatores ambientais e/ou sociais possam ter co-evoluído e contribuído para a complexidade na comunicação de mamíferos (Lemasson, 2011; Freeberg et al., 2012; Pollard e Blumstein, 2012). A dificuldade em identificar a relação entre complexidade comunicativa, social e ecológica pode ser atribuída a numerosas variáveis que distinguem cada um desses domínios, assim como pode haver interações inesperadas entre eles (Pollard e Blumstein, 2012; Manser et al., 2014). Para aumentar essa compreensão, precisamos identificar atributos sociais e ecológicos e como estes se relacionam a atributos comunicativos específicos (Pollard e Blumstein, 2012; Manser et al., 2014). Desta forma, o estudo comparado entre espécies do mesmo táxon, que diferem em seus sistemas sociais e ecológicos, pode possibilitar uma melhor análise para este entendimento como é o caso da infraordem dos caviomorfos.

O grupo dos caviomorfos é composto por 13 famílias, 56 gêneros e 246 espécies (Ojeda et al., 2016). Esta ampla diversidade de espécies, que possuem também variedade no tamanho e composição dos grupos sociais (Redford e Eisenberg, 1992; Emmons e Feer, 1997; Nowak, 1999; Maher e Burger, 2016), nos tipos de sistemas de acasalamento (Herrera, 2016), nos tipos de sistemas sociais (Redford e Eisenberg, 1992; Maher e Burger, 2016) e nos repertórios acústicos (Eisenberg, 1974; Francescoli et al., 2016), possibilita analisar fatores que estejam associados à complexidade vocal. Outro aspecto que torna os caviomorfos um grupo interessante para investigar sobre as possíveis pressões seletivas do ambiente sobre a complexidade vocal, é por estarem presentes em uma variedade de habitats, desde ambientes de florestas tropicais úmidas até regiões desérticas (Redford e Eisenberg, 1992; Emmons e Feer, 1997; Ojeda et al., 2016). Além destas características do táxon, há disponibilidade de dados na literatura sobre o repertório acústico de várias espécies de caviomorfos (Francescoli et al. 2016; cap. 1 e 2 desta tese) o que possibilita uma análise mais completa.

Neste contexto, o objetivo do presente estudo foi analisar quais fatores sociais, reprodutivos e ecológicos têm papel na complexidade vocal de 10 espécies de roedores caviomorfos e assim analisar a relação entre a complexidade social e vocal neste grupo e, desta forma, testar a hipótese da complexidade comunicativa de Freeberg et al. (2012). Predizemos que quanto maior a complexidade social, maior será a complexidade vocal em caviomorfos. Hipotetizamos ainda que a relação direta entre complexidade vocal e social será mais forte em contextos específicos de emissão (agonísticos, alarme e amigáveis).

Material e Métodos

Coleta de Dados

Foram coletadas informações da literatura sobre o repertório vocal total, do repertório amigável, agonístico e de alarme de 10 espécies de roedores caviomorfos: *Ctenomys talarum, Cuniculus paca, Chinchilla lanígera, Dasyprocta leporina, Cavia aperea, Cavia porcellus, Spalacopus cyanos, Kerodon rupestris e Octodon degus* (Tabela 1). Adicionalmente, por meio de revisão de literatura foram obtidas informações sobre características morfológicas, reprodutivas, sociais e ecológicas destas mesmas espécies. Para esta coleta de informações foram consultados 55 artigos científicos, cinco livros, duas teses de doutorado e três dissertações de mestrado. Estas referências foram obtidas a partir da busca nas bases de dados: *Web of Science*, Dedallus (USP), Google acadêmico, *ResearchGate* e Banco de Teses e Dissertação da Coordenação de Aperfeiçoamento do Pessoal de Nível Superior (CAPES).

Foram considerados trabalhos que descreveram o repertório acústico das 10 espécies analisadas, com detalhadamento sobre a estrutura e parâmetros acústicos, além do contexto comportamental para evitar duplicação de vocalizações similares mas com denominações distintas. Também consideramos apenas as vocalizações de animais adultos, primeiro porque para a maior parte das espécies não há dados de filhotes e também devido às possíveis mudanças de origem ontogênica que poderiam interferir nas conclusões sobre o tamanho e diversidade acústica do repertório vocal das espécies. Outro critério adotado foi a exclusão de sinais mecânicos, como batidas de dentes. Adotamos este procedimento porque tais chamados não são de origem vocal e podiam trazer inconsistência às análises. Também excluímos a contagem de efeitos não lineares,

gradação, transição e combinação das vocalizações devido a não disponibilidade de informações para todas as 10 espécies aqui analisadas.

Critérios para Mensuração do Repertório Acústico

Para contabilização das vocalizações descritas com notas múltiplas ou combinadas (Figura 1), adotamos os seguinte critérios: a) não contabilizadas, caso as notas A e B já tivessem sido descritas anteriormente; b) contabilizadas, caso um chamado fosse uma combinação das notas descritas anterioremnte (A ou B) com um novo elemento C que resultaria em um tipo de chamado novo AC ou BC e, portanto, adicionado ao repertório. Aplicamos estas regras para todas as espécies analisadas. Devido a estes critérios, os repertórios das diferentes espécies aqui apresentados podem não coincidir com o número total de tipos vocais indicado pelos respectivos autores dos estudos.

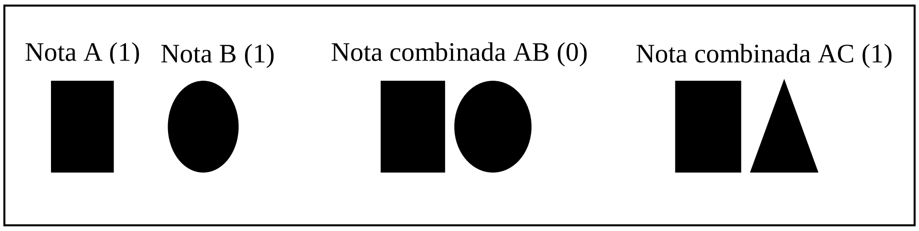

Figura 1. Esquema de contagem de notas múltiplas ou combinadas utilizadas neste trabalho. Número de notas contabilizadas entre

Além da contagem do tamanho do repertório acústico de cada espécie, analisamos se estas vocalizações foram emitidas durante contextos agonísticos, amigáveis ou de alarme,denominados como repertório agonístico, repertório amigável e repertório de alarme de cada espécie. No repertório agonístico foram incluídas as vocalizações emitidas durante encontros agressivos e de ameaça entre coespecíficos. No

repertório amigável, consideramos todas as vocalizações de contato entre coespecíficos, envolvendo ou não função de coesão do grupo. Por sua vez, no repertório de alarme incluímos os chamados emitidos em contextos de alerta e de estresse negativo (*distress*) . Vocalizações emitidas em mais de um contexto foram contabilizadas novamente e, também por este motivo, os tamanhos desses repertórios podem diferir dos estudos originais de cada espécie.

Variáveis Usadas para Analisar a Complexidade Vocal

Com base na literatura consultada, selecionamos 10 variáveis entre características sociais, reprodutivas e ecológicas das 10 espécies para testar a hipótese da complexidade comunicativa em caviomorfos. Para algumas destas variáveis, adotamos escores que variaram de 1 a 6,5 de acordo com o número de possibilidades dos arranjos encontrados para os animais em vida livre e descritos na literatura (Tabela 1). Os escores, portanto, foram adotados de forma crescente, partindo desde a ausência ou menor característica em que adotamos o escore 1 até o número mais complexo da referida característica (6,5).

Com relação às características sociais, quatro variáveis foram escolhidas: *composição do grupo, tamanho máximo do grupo, tipo de sistema social* e *forrageio social*. A variável *composição do grupo*, corresponde ao número de machos e de fêmeas adultos que compõem os grupos sociais. Esta variável pode ser composta por um macho para uma fêmea (escore 1), um macho para várias fêmeas (escore 2) ou vários machos para várias fêmeas (escore 3). O *tamanho máximo do grupo*, diz respeito ao número máximo de indivíduos adultos que formam o grupo. Definimos o *tipo de sistema social* pela unidade social descrita para os indivíduos da espécie: solitária (escore 1); solitária ou par monogâmico (escore 2); par monogâmico (escore 3); par monogâmico ou harém

(escore 3) e harém (escore 4) (Tabela 1). O *forrageio social* corresponde a busca e consumo de alimentos realizado em grupo. Dessa forma, as espécies que realizam o forrageamento em grupo (escore 2) e aquelas que forrageiam solitárias (escore 1).

Tabela 1

Tamanho dos repertórios e características sociais, reprodutivas e ecológicas de 10 espécies de roedores caviomorfos. Escores usados estão representados entre parêntesis.

ESPÉCIE	TRV	RAG	RAM	RAL	CGR	TG	TSC	SAC	FSO	TP	PAT	TDI	THA	NCV	FONTE
Ctenomys talarum	4	1	0	1	1 M e >2F (2)	1	Solitário (1)	Poliginia (2)	Solitário (1)	5	Diurno (1)	Folívero (1)	Semi-fossorial (2)	Pastagem, savana (2,5)	1, 2 ,3 4, 5
Cunicullus paca	5	3	0	2	1M - 1F (1)	1	Solitário/Par monogâmico (2)	Monogamia (1)	Solitário (1)	1	Noturno (2)	Frugívero (2)	Tocas (3)	Florestas abertas e densas (6,5)	6, 7, 8, 9, 10, 11, 12, 13
Chinchila lanigera	7	1	1	1	1M - 1F (1)	2	Par monogâmico (3)	Monogamia (1)	Solitário (1)	3	Noturno (2)	Folívero (1)	Terrestre (5)	Vegetação desértica (2,5)	14, 15, 16, 17, 18,19
Dasyprocta leporina	8	4	1	2	1M - 1F (1)	2	Par monogâmico (3)	Monogamia (1)	Solitário (1)	3	Diurno (1)	Frugívero (2)	Terrestre (5)	Florestas abertas (6)	10, 20, 21, 22, 23, 24
Cavia aperea	7	4	2	3	1 M e >2 F (2)	3	Par monogâmico-Harém(4)	Poliginia de defesa de fêmeas (2)	Grupo (2)	5	Diurno (1)	Folívero (1)	Terrestre (5)	Pastagem, savanas (3,5)	2, 25, 26, 27, 28, 29
Cavia porcellus	9	3	2	3	1 M e >2 F (2)	3	Par monogâmico-Harém(4)4	Poliginia de defesa de fêmeas (2)	Grupo (2)	5	Diurno (1)	Folívero (1)	Terrestre (5)	Pastagem, (1)	30, 31, 32, 33, 34, 35

Hydrochoerus hydrochaeris	8	2	4	2	> 2M e >2F (3)	18	Harém (5)	Poliginia/Promiscuidade (3)	Grupo (2)	7	Diurno (1)	Folívero (1)	Semi-aquático (4)	Savana aberta (2,5)	36, 37, 38, 39, 40, 41, 42, 43
Spalacopus cyanos	9	2	4	1	> 2M e >2F (3)	15	Par monogâmico/ Harém(4)	Poliginia (2)	Grupo (2)	5	Diurno (1)	Folívero (1)	Fossorial (1)	Pastagem, savana (2,5)	44, 45, 46, 47, 48, 49
kerodon rupestris	10	6	1	3	1 M e >2 F (2)	5	Harém (5)	Poliginia de defesa de recursos (2)	Solitário (1)	3	Diurno (1)	Folívero (1)	Tocas (3)	Arbustos, afloramentos rochosos (2,5)	21,50, 51, 52, 53, 54
Octodon degus	13	5	4	3	> 2M e >2F (3)	9	Harém (5)	Poliginia/promiscuidade (3)	Grupo (2)	6	Diurno (1)	Folívero (1)	Semi-fossorial (2)	Arbustos, afloramentos rochosos (2,5)	14, 16, , 55, 56, 57, 58, 59, 60, 61, 62, 63, 64

Abreviações : tamanho do repertório vocal (TRV), repertório agonístico (RAG), repertório amigável (RAM), repertório de alarme (RAL), composição do grupo de adultos (CGR), tamanho máximo do grupo de adultos (TMG), tipo de sistema social (TSC), sistema de acasalamento (SAC), forrageio social (FSO), tamanho máximo da prole (TMP), período de atividade (PAT), tipo de dieta (TDI), tipo de habitat (THA), nível de cobertura vegetal (NCV), massa corporal média em gramas (MCM), dimorfismo sexual (DSE).

Fonte: 1- Busch et al. 1989; 2- Redford e Eisenberg, 1992; 3- Zenuto et al. 1999a, 4-Zenuto et al., 2002; 5- Schleich e Busch, 2002; 6- Woods, 1984; 7- Smythe, 1987; 8- Smythe & Brown de Guanti (1993); 9- Pérez, 1992; 10- Emmons e Feer, 1997; 11- Beck-king et al; 1999; 12-Goulart et al., 2009; 13- Cap. 1 desta tese; 14- Weir, 1974; 15- Mohlis (1983);16- Nowak, 1999; 17- Bartl, 2006; 18- Pavia, 2003; 19- Vanderlip, 2006; 20- Dubost, 1988; 21- Eisenberg e redford, 1989; 22- capítulos um e dois desta tese; 23- Silvius e Fragoso, 2003; 24- Jorge e Perez, 2005; 25-Monticelli e Ades, 2013; 26- Rood, 1972; 27- Asher et al., 2008; 28- Sachser et al. 1999; 29- Asher et al., 2004; 30- Monticelli, 2005; 31 Künzl e Sachser,1999-; 32- Rood, 1972; 33- Berryman, 1976; 34- Sachser, 1986; 35- Sachser 1998; 36 Suzuki, 2015-; 37-Herrera et al., 2011; 38- Azcarate, 1980; 39-Mones e Ojasti, 1986; 40- Herrera e Mcdonald, 1987; 41- Corriale et al. (2013); 42- Salas, 1999; 43- Barros et al. 2011; 44-Veitl et al, 2000; 45-Begall e Gallardo, 2000; 46- Reig, 1970; 47- Contreras e Gutierrez, 1991; 48- Begall et al. 1999; 49- Urrejola et al., 2005; 50-Tasse, 1986; 51-Adrian e Sachser 2011; 52- Lacher, 1981; 53- Mares e Ojeda, 1982; 54- Alencar Jr, 2012;; 55- Ebensperger et al., 2011; 56- Quirici et al., 2010- 57- Hayes et al. 2009; 58- Ebensperger et al., 201259- Kleiman, 1970; 60-; 61- Fulk, 1976; 62- Ebensperger et al., 2004; 63- Soto-Gamboa et al., 2005; 64- Long, 2007.

Como característica reprodutiva consideramos o *sistema de acasalamento.* O *sistema de acasalamento* diz respeito ao tipo de estratégia reprodutiva utilizada pela espécie. Assim, dentre as possibilidades de acasalamento, encontramos os tipos monogâmico (escore 1), poligínico (escore 2) e promíscuo (escore 3).

Entre as características ecológicas das espécies, consideramos: *período de atividade, tipo de dieta, tipo de habitat, índice de cobertura vegetal* e *tamanho da prole.* Para o tipo de dieta, foi considerado a dieta primária da espécie em questão: partes de plantas e gramíneas (escore 1), frugívoros (escore 2). O *tipo de habitat* da espécie foi outra variável analisada e correspondeu ao hábito fossorial (escore 1), hábito semi-fossorial (escore 2), uso de tocas (escore 3), hábito semi-aquático (escore 4) e hábito terrestre (escore 5). Utilizamos o *índice de cobertura vegetal* usado por Ebensperger e Blumstein, (2006) que corresponde a quantidade de cobertura vegetal presente em cada tipo de habitat. Este índice variou de sem cobertura (escore 1), cobertura baixa (escore 2,5), cobertura média (escore 3,5), cobertura alta (escore 6) e cobertura máxima (escore 6,5). Finalmente, o *tamanho máximo da prole* corresponde ao número máximo de filhotes por parição de cada espécie. O período de atividade das espécies, diz respeito ao período de sono e vigília, se diurnos (escore 1) ou se noturnos (escore 2).

Outros dados da história natural, no entanto, tais como estrutura social, tamanho da área de vida e sobreposição da área de vida entre machos e fêmeas não foram incluídos na análise por não haver tais informações para todas as espécies aqui estudadas.

Análise Estatística

Utilizamos a Análise de Componentes Principais (ACP) para explorar e reduzir o número de variáveis reprodutivas, sociais e ecológicas que poderiam estar relacionadas com o repertório acústico das espécies de caviomorfos estudadas. Para esta análise aplicamos o procedimento de Técnicas Exploratórias Multivariadas (*Multivariate Exploratory Techniques procedure*) que torna possível analisar em conjunto variáveis com e sem distribuição normal (Jolliffe, 2002). Para este fim, inicialmente padronizamos as medidas (variável original – média da variável original/desvio padrão da variável original). Tal procedimento permitiu a comparação de dados com unidades e variâncias diferentes – por exemplo, massa corporal (kg) e tamanho da prole. Para decidir quais componentes principais considerar para a interpretação da ACP usamos o critério de Kaiser-Guttman (autovalores > 1). Em seguida, aplicamos o teste de Lilliefors para as variáveis selecionadas pela ACP.

As variáveis que apresentaram distribuição normal foram analisadas por meio de uma matriz de correlação de Pearson. Em seguida, os dados correlacionados foram analisados por meio de análise de regressão linear. Os demais dados que não apresentaram distribuição normal, foram analisados por meio de correlações de Sperman. Todas as análises foram realizadas no software Statistica versão 7.1 (Statsoft, 2005), com nível de significância de $P<0,05$.

Resultados

Na análise de componentes principais (ACP) apenas os quatro primeiros componentes apresentaram autovalores >1,0 e em conjunto explicaram 85,6% da variância (Tabela 2). No componente principal 1 (CP 1), explicando 49,6% da variância, o repertório acústico total aparece positivamente correlacionado com o repertório amigável, tipo de sistema social, tamanho do grupo, composição do grupo, sistema de

acasalamento e tamanho da prole, que por sua vez foram negativamente correlacionados com forrageio social, período de atividade, tipo de dieta e índice de cobertura vegetal (Tabela 2, eixo x da Figura 2). No componente principal 2 (CP 2), explicando 16,9% da variância, o repertório agonístico e o repertório de alarme aparecem correlacionados (Tabela 2, eixo y da Figura 2). No terceiro componente principal (CP3), explicando 11,2% da variância, a massa corporal apareceu isolada (Tabela 2). No quarto componente principal (CP 4), explicando 7,9% da variância, apareceu isolado o tipo de habitat ocupado pelas espécies (Tabela 2).

Tabela 2 Matriz dos componentes principais, autovalores, variância e variância acumulada entre as variáveis acústicas, sociais e ecológicas nos quatro primeiros componentes principais (CP 1, CP 2 CP 3 e CP 4). Os valores em negrito indicam as variáveis com maior peso na análise em cada componente.

Variáveis vocais, sociais e ecológicas	CP 1	CP 2	CP 3	CP 4
Tamanho máx. do repertório vocal	**0,70**	0,48	-0,12	0,03
Repertório agonístico	0,26	**0,91**	-0,11	0,27
Repertório amigável	**0,88**	-0,08	0,25	-0,17
Repertório de alarme	0,37	**0,86**	-0,08	-0,10
Composição do grupo (adultos)	**0,95**	-0,22	0,07	0,16
Tamanho máximo do grupo (adultos)	**0,78**	-0,28	0,47	0,02
Tamanho máximo da prole	**0,85**	-0,30	0,00	-0,27
Tipo de sistema social	**0,79**	0,44	0,08	-0,17
Sistema de acasalamento	**0,94**	-0,06	0,06	0,08
Massa corporal (g)	-0,05	-0,03	**0,94**	-0,04
Dimorfismo sexual	**-0,75**	-0,19	-0,14	-0,43
Período de atividade	**-0,67**	-0,13	0,16	-0,01
Tipo de dieta	**-0,69**	0,35	0,53	0,15
Tipo de habitat	-0,33	0,36	0,01	**-0,83**
Nível de cobertura vegetal	**-0,68**	0,43	0,48	0,02
Forrageio social	**-0,81**	0,01	-0,13	0,35
Autovalores	7,93	2,70	1,80	1,27
Variância (%)	49,55	16,86	11,23	7,94
Variância acumulada (%)	49,55	66,42	77,64	85,58

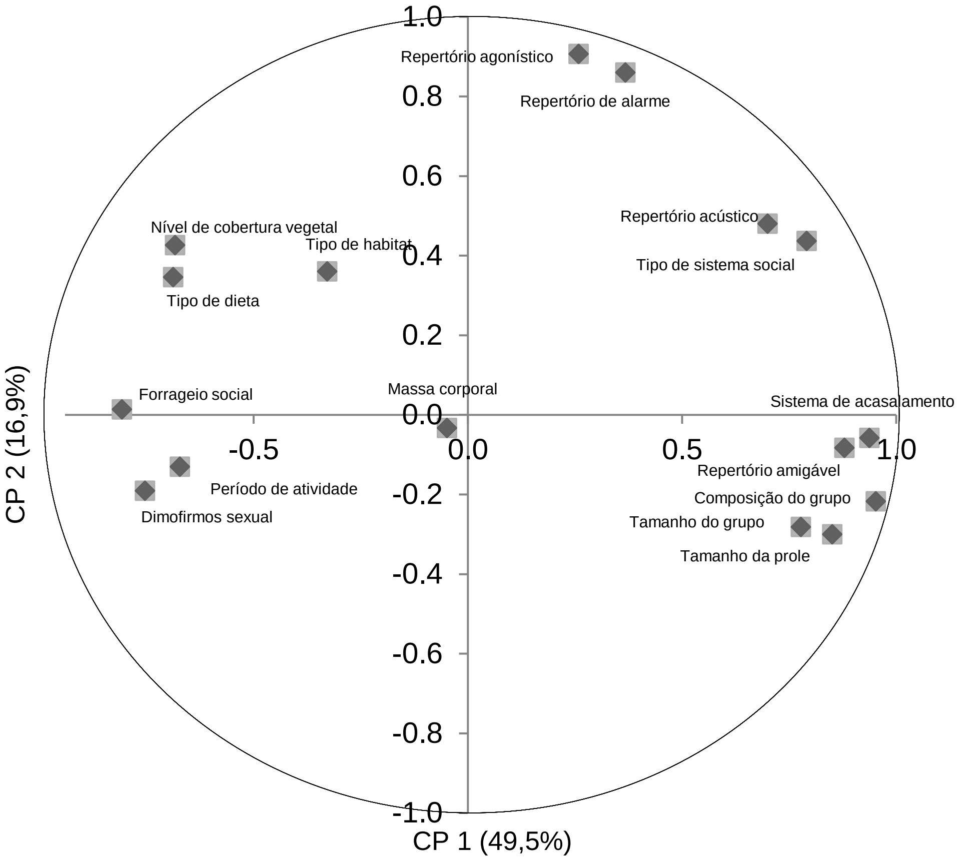

Figura 2. Correlação entre as variáveis vocais, sociais e ecológicas nos dois primeiros componentes principais (CP1 e CP2).

Por meio da matriz de correlação de Pearson o tamanho do repertório vocal foi positivamente correlacionado ao repertório amigável ($r_{Pearson} = 0,69$; $P < 0,05$) e ao tipo de sistema social ($r_{Pearson}=0,84$; $P<0,05$). Por sua vez, o repertório amigável foi positivamente correlacionado à composição do grupo ($r_{Pearson} = 0,85$; $P < 0,05$), tamanho da prole ($r_{Pearson} = 0,76$; $P < 0,05$), tipo de sistema social ($r_{Pearson} = 0,75$; $P < 0,05$) e sistema de acasalamento ($r_{Pearson} = 0,75$; $P < 0,05$). Finalmente, por meio das análises de correlação de Sperman verificamos correlação entre o tamanho do repertório vocal com o tamanho máximo do grupo ($r_{Spearman} = 0,73$; $P < 0,05$), enquanto o repertório amigável

foi correlacionado com tamanho máximo do grupo ($r_{Spearman} = 0,91$; $P < 0,05$) e com o forrageio social ($r_{Spearman} = 0,90$; $P < 0,05$).

A análise de regressão mostrou que quanto mais complexo o tipo de sistema social, que variou de solitário a harém, o repertório vocal dos caviomorfos aumentou de forma linear seguindo a equação $y = 1,58x + 2,29$ ($F_{1, 8} = 19,65$, $R^2 = 0,71$, $P = 0,02$, Figura 3).

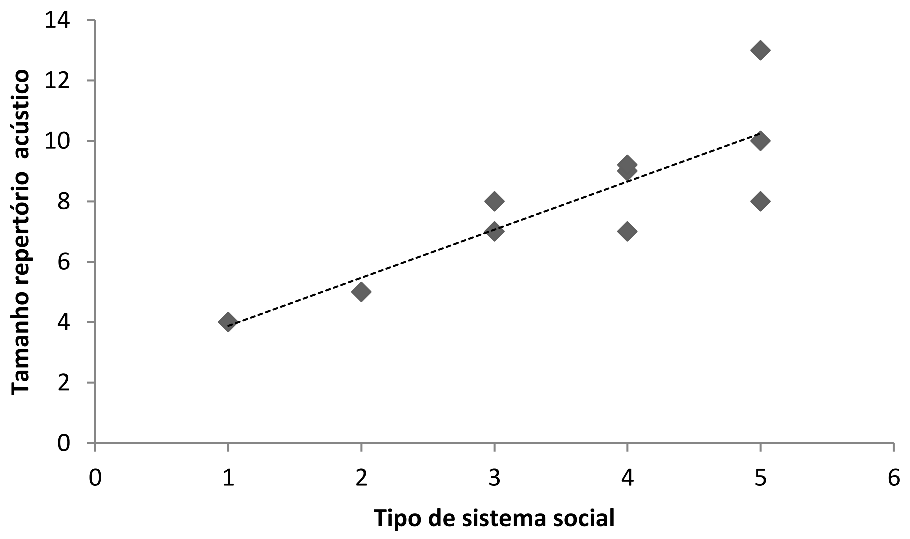

Figura 3. Regressão linear entre tamanho do repertório acústico e complexidade do tipo de sistema social seguindo a equação $y = 1,58x + 2,29$ ($F_{1, 8} = 19,65$, $R^2 = 0,71$, $P = 0,02$).

Por sua vez, o repertório amigável aumentou linearmente com o aumento de machos em relação às fêmeas no grupo (composição do grupo) de acordo com a equação: $y = 1,67x - 1,43$ ($F_{1, 8} = 21,39$, $R^2 = 0,73$, $P < 0,002$, Figura 4A); com o aumento no número de filhotes na ninhada (tamanho da prole) de acordo com a

equação: y = 0,69x - 1,05 ($F_{1, 8}$ = 11,00, R^2 = 0,58, P < 0,01, Figura 4B); com a variação de solitário a harém (sistema social) de acordo com a equação: y = 0,89x - 1,30 ($F_{1, 8}$ = 10,50, R^2 = 0,57, P < 0,01, Figura 4C); e com a variação do comportamento monogâmico ao promíscuo (sistema de acasalamento) de acordo com a equação: y = 1,61x - 1,16 ($F_{1, 8}$ = 10,02, R^2 = 0,56, P < 0,01, Figura 4D).

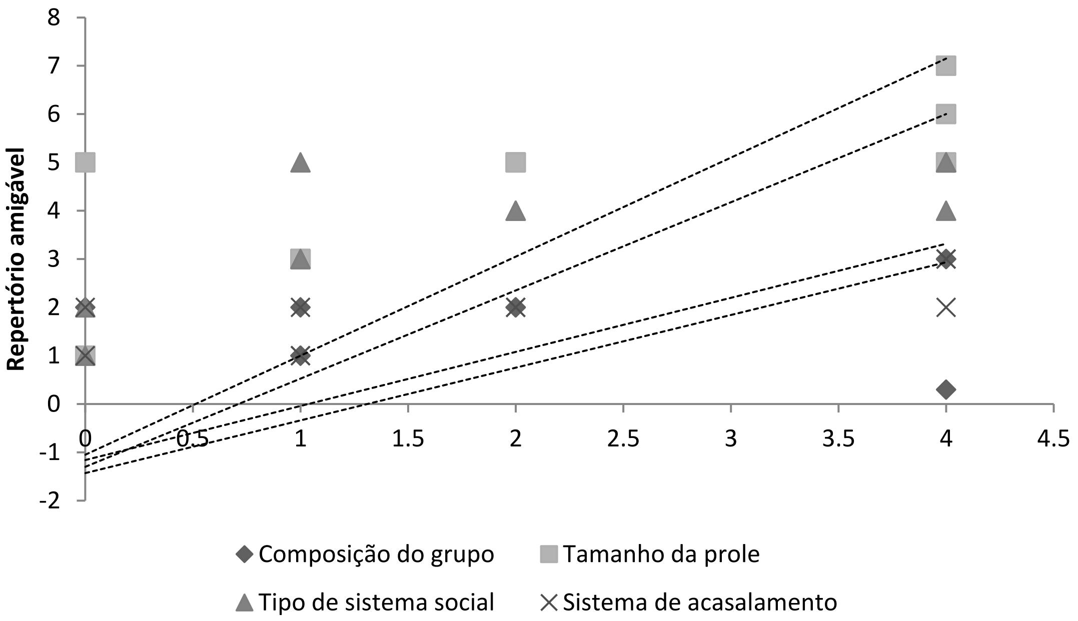

Figura 4. Relação entre o repertório amigável e as variáveis sociais: (A) composição do grupo de acordo com a equação: y = 1,67x - 1,43 ($F_{1, 8}$ = 21,39, R^2 = 0,73, P < 0,002). (B) Tamanho da prole de acordo com a equação: y = 0,69x - 1,05 ($F_{1, 8}$ = 11,00, R^2 = 0,58, P < 0,01). (C) Tipo de sistema social de acordo com a equação: y = 0,89x - 1,30 ($F_{1, 8}$ = 10,50, R^2 = 0,57, P < 0,01). (D) Sistema de acasalamento de acordo com a equação: y = 1,61x - 1,16 ($F_{1, 8}$ = 10,02, R^2 = 0,56,

Discussão

Os resultados encontrados confirmaram nossa hipótese de que quanto maior a complexidade social, maior a complexidade vocal nas espécies de caviomorfos analisadas nesse estudo. De acordo com nossas predições, encontramos aumento no tamanho do repertório vocal com a variação de solitário a harém (sistema social). Dessa forma, esse estudo contribui para caracterizar quais fatores sociais estão relacionados à complexidade vocal nessas espécies. Adicionalmente, reforçando esta predição, encontramos relações entre o repertório vocal amigável com o sistema de acasalamento e o tamanho da prole.

A relação entre complexidade social e vocal encontrada no presente estudo corrobora com a hipótese da complexidade comunicativa de Freeberg et al. (2012). Diferentes atributos da complexidade social estão ligados a diferentes atributos da complexidade vocal (Pollard e Blumstein, 2012). Tais evidências, como encontrada em nosso estudo, que relacionou tamanho do repertóriocom tipo de sistema social; e sistema de acasalamento e tamanho da prole com tamanho do repertório amigável, também são relatadas em outros estudos. O papel social demográfico, por exemplo, foi relacionado com o tamanho do repertório de alarme em *Marmota* spp. e *Cynomys* spp., *Spermophilus* spp. (Blumstein e Armitage, 1997). Uma relação entre o sistema de acasalamento e o tamanho do repertório vocal também foi encontrada em *Erignathus barbatus* e *Leptonychotes weddellii* (Stirling e Thomas, 2003). Outro exemplo de variável associada ao tamanho do repertório vocal é o tamanho do grupo social e o tempo gasto em catação social; esta relação foi encontrada para 46 espécies de primatas não humanos (MacComb e Semple, 2005). Esta mesma relação (tamanho do grupo) foi associada com a complexidade estrutural do chamado *chick-a-dee* em *Poecile carolinenses* (Freeberg, 2006). Outra variável que está relacionada com a complexidade

vocal é o tipo de sistema social e a variabilidade acústica estrutural nas espécies *Cercopthecus neglectus*, *C. campibelli* e *Cercocebus torquatus* (Bouchet et al., 2013).

Nossos resultados mostram que o tamanho do repertório vocal amigável aumenta com o tamanho da prole. Chamados amigáveis são usados para manter a coesão do grupo durante deslocamentos a longas distâncias (Janik e Slater, 1998), e também servem para coordenar atividades entre filhotes e seus progenitores (Sousa-Lima et al., 2002; Dombroski et al. no prelo). Filhotes emitem chamados de contato quando separados de suas mães (Sousa-Lima et al., 2002, 2008; Dos Santos et al., 2013), muitos destes chamados possuem identidade vocal que permitem distinção entre os membros do grupo e poderiam variar ainda mais o repertório vocal como ocorre em morcegos (Wilkinson, 2003), pássaros (Beecher, 1990), e roedores (Blumstein e Armitage, 1997; Pollard e Blumstein, 2012). A função de contato e a identidade vocal entre filhotes dependentes e suas mães, portanto, pode explicar porque encontramos em nosso estudo que espécies com maior número de filhotes apresentam maior número de chamados amigáveis.

O tamanho do repertório amigável também aumenta com o tipo de sistema social (de solitário a harém). O procedimento adotado neste estudo de dividir os chamados dos repertórios vocais de acordo com o contexto de emissão em categorias, ao invés de analisar somente o total de vocalizações do repertório, possibilitou identificar que o número de chamados amigáveis é maior em espécies mais sociais quando comparado a espécies solitárias. Esta relação entre repertório amigável e fatores sociais pode ser explicada pelo fato de que os laços sociais de um grupo social são refletidos no uso de suas vocalizações ou na estrutura dos chamados (Lemasson et al., 2011), influenciando sua variabilidade acústica (Snowdon et al., 1997; Griebel e Oller, 2008). Essa

complexidade acústica pode ser uma maneira de codificar informações sobre a identidade do emissor ou o contexto de emissão (Bouchet et al., 2013).

Nossos resultados também estão de acordo com os obtidos em outro estudo que comparou os tipos de chamados funcionalmente diferentes em repertórios vocais de cinco espécies da família Herpestidae (Manser et al., 2014). Neste estudo, os autores sugerem a presença de uma maior variabilidade de tipos de chamados amigáveis em espécies mais sociais quando comparados a espécies solitárias (Manser et al., 2014). Por outro lado, estes autores não encontraram relação entre os repertórios agonísticos e de alarme com as variáveis sociais entre as espécies solitárias e mais sociais, corroborando com os resultados encontrados no presente estudo. Uma possível explicação seria que a flexibilidade vocal ou variabilidade vocal tem se mostrado mais presente entre os chamados amigáveis (Griebel e Oller, 2008). Sinais vocais em contextos de alarme, por sua vez, tendem a ser mais conspícuos e menos plásticos em relação a características vocais e usos gerais, além de apresentar convergência entre as espécies (Griebel e Oller, 2008; Candiotti et al., 2012). Esses chamados geralmente advertem sobre a presença de predadores e possuem maior valor de sobrevivência e, portanto precisam ter menos variação e entendimento imediato do que os chamados amigáveis (Snowdon e Elowson, 1999). Por sua vez, chamados agressivos geralmente são emitidos em contextos muito similares, como durante a alimentação e encontros agonísticos e não variam muito nos tipos de chamados produzidos (Manser et al., 2014) .

O repertório amigável também foi correlacionado com as características reprodutivas das espécies de caviomorfos analisadas. Ambos, tamanho da prole e sistema de acasalamento estão diretamente relacionados ao tamanho do grupo. Verificamos o aumento na diversidade deste tipo de chamado com o tipo de sistema de acasalamento (de monogâmico a promíscuo). Herrera (2016) relata que quanto mais

promíscuo o sistema de acasalamento em mamíferos, menos os machos conseguem monopolizar grupos de fêmeas. Este comportamento levaria a uma maior competição por parceiros (Herrera, 2016), o que provavelmente requer um repertório mais variado. Este resultado corrobora a proposição de Pollard e Blumstein (2012) que predizem o aumento no tamanho do repertório vocal usado para atrair ou impressionar parceiros sexuais de acordo com a complexidade do sistema de acasalamento. O mesmo foi verificado para aves na família Troglodytidae, em que machos de espécies poligínicas têm repertório vocal maior que machos de espécies monogâmicas (Kroodsma, 1977), no entanto este autor apontou a presença de seleção sexual. Machos de focas, das espécies *Erignathus barbatus* e *Leptonychotes weddellii,* também apresentam aumento no tamanho do repertório vocal com o aumento da complexidade do sistema de acasalamento (Stirling e Thomas, 2003).

Entre as características ecológicas avaliadas nesse estudo, o tipo de habitat explicou uma parte da variância, porém, não foi correlacionado com o tamanho do repertório vocal. Freeberg et al. (2012) sugerem que fatores ecológicos podem atuar contra as pressões geradas pela complexidade social. Os autores relatam que, é essencial excluir fatores não sociais para testar sua hipótese, pois acreditam que apenas os fatores sociais é que levariam a maior complexidade vocal, uma vez que os não sociais poderiam atuar de forma isolada ou em conjunto com outros fatores sociais (Freeberg et al., 2012). Até o momento, poucos estudos têm encontrado quais fatores sociais e não sociais direcionam para a evolução da complexidade na comunicação (Freeberg et al., 2012; Ord e Garcia-Porta, 2012). Neste sentido, o presente estudo contribui com o aumento de informações para uma melhor compreensão da relação entre complexidade social e vocal, mostrando que nas espécies de caviomorfos aqui analisadas, a complexidade na comunicação sugere estar principalmente relacionada a fatores sociais,

ecológicos e reprodutivos que atuam em conjunto aumentando a complexidade vocal nestes animais.

Referências

Adrian, O. & Sachser, N. 2011. Diversity of social and mating systems in cavies: a review. Journal of Mammalogy 92: 39–53.

Alencar, R. N. Jr. 2012. O repertório acústico de um especialista de rochedos da caatinga, o mocó [dissertação de mestrado]. São Paulo: Departamento de Psicologia Experimental, Universidade de São Paulo.

Asher, M., Lippmann, T., Epplen, J.T., Kraus, C. & Trillmich, F. & Sachser, N. 2008. Large males dominate: ecology, social organization, and mating system of wild cavies, the ancestors of the guinea pig. Behavioral Ecology and Sociobiology 62: 15–21.

Asher, M., Spinelli de Oliveira, E. & Sachser, N. 2004. Social system and spatial organization of wild guinea pigs (*Cavia aperea*) in a natural population. Journal of Mammalogy 85: 788–96.

Azcarate, T. 1980. Sociobiologia y manejo del capibara (*Hydrochoerus hydrochaeris*). Donana Acta Vertebrata 7: 1—228.

Barros, K. S., Tokumaru, R. S., Pedroza, J. P. E Nogueira, S. S. d. C. 2011. Vocal repertoire of captive capybara (*Hydrochoerus hydrochaeris*): structure, context and function. Ethology 117: 83-93.

Bartl, J. 2006. Lautäußerungen der Chinchillas im Sozialverband (Doctoral dissertation, lmu).

Beck-King, H. & von Helversen, O. 1999. Home range, population density, and food resources of *Agouti paca* (Rodentia: Agoutidae) in Costa Rica: a study using alternative methods. Biotropica 31: 675–85.

Beecher, M. D. 1990. The evolution of parent-offspring recognition in swallows. In D. Dewsbury (Ed.), Contemporary issues in comparative psychology (pp. 360–380). Sunderland, MA: Sinauer Associates.

Begall, S. & Gallardo, M.H. 2000. *Spalacopus cyanus* (Rodentia: Octodontidae): an extremist in tunnel constructing and food storing among subterranean mammals. Journal of Zoology London 251: 53–60.

Begall, S., Burda, H., & Gallardo, M. H. 1999. Reproduction, postnatal development, and growth of social coruros, *Spalacopus cyanus* (Rodentia: Octodontidae), from Chile. Journal of Mammalogy 80: 210-217.

Bernal, X. E., Akre, K. L., Baugh, A. T., Rand, A. S., & Ryan, M. J. 2009. Female and male behavioral response to advertisement calls of graded complexity in túngara frogs, *Physalaemus pustulosus*. Behavioral ecology and sociobiology 63: 1269-1279.

Berryman JC. 1976. Guinea pig vocalizations, their structure, causation and function. Zeitschrift fu¨r Tierpsychologie 41:80–106.

Blumstein, D. T., & Armitage, K. B. 1997. Does sociality drive the evolution of communicative complexity? A comparative test with ground-dwelling sciurid alarm calls. The American Naturalist 150: 179-200.

Bouchet, H., Blois-Heulin, C., & Lemasson, A. 2013. Social complexity parallels vocal complexity: a comparison of three non-human primate species. Frontiers in psychology, 4, 390.

Bradbury, J. W & Vehrencamp, S. L. 1998. Principles of Animal Communication. Sinauer Associates, Canada.

Busch C, Malizia AI, Scaglia OA, Reig Oa 1989. Spatial distribution and attributes of a population of *Ctenomys talarum* (Rodentia: Octodontidae). J Mammal 70: 204–208.

Candiotti, A., Zuberbühler, K., & Lemasson, A. 2012. Convergence and divergence in Diana monkey vocalizations. Biology Letters 8: 382-385.

Contreras, L. C., & Gutiérrez, J. R. 1991. Effects of the subterranean herbivorous rodent Spalacopus cyanus on herbaceous vegetation in arid coastal Chile. Oecologia 87: 106-109.

Corriale, M. J., Muschetto, E., & Herrera, E. A. 2013. Influence of group sizes and food resources in home-range sizes of capybaras from Argentina. Journal of Mammalogy, 94: 19-28.

Crockford, C. & Boesch, C. 2005. Call combinations in wild chimpanzees. Behaviour 142; 397–421.

Devillard, S., Allainé, D., Gaillard, J. M., & Pontier, D. 2004. Does social complexity lead to sex-biased dispersal in polygynous mammals? A test on ground-dwelling sciurids. Behavioral Ecology 15: 83-87.

Dombroski, J. R. G.; Parks, S. E.; Groch, K. R.; Flores, P. A. C. e Sousa-Lima, R. S. (*no prelo*). *Eubalaena australis* mother-calf pairs' upcall production may be independent of diel period in a nursery area off Brazil.

Dos Santos, E.; Tokumaru, R. S.; Nogueira Filho, S. L. G.; Nogueira, S. S. C. 2014. The effects of unrelated offspring whistle calls on capybaras (*Hydrochoerus hydrochaeris*). Braz. J. Biol. 74: S171-S176.

Dubost, G. 1988. Ecology and social life of the red acouchy, *Myoprocta exilis*; comparison with the orange rumped agouti, *Dasyprocta leporina*. Journal of Zoology 214: 107-123.

Ebensperger, L.A., Sobrero, R., Vargas, F. et al. 2012a. Ecological drivers of group living in two populations of the communally rearing rodent, *Octodon degus*. Behavioral Ecology and Sociobiology 66: 261–74.

Ebensperger L. A., Chesh, A. S. Castro, R. A. et al. 2011. Burrow limitations and group living in the communally rearing rodent, *Octodon degus.* Journal of Mammalogy 92: 21–30.

Ebensperger, L.A. & Blumstein, D.T. 2006. Sociality in New World Hystricognath rodents is linked to predators and burrow digging. Behavioral Ecology 17: 410–18.

Ebensperger, L. A., Hurtado, M. J., Soto-Gamboa, M., Lacey, E. A., & Chang, A. T. 2004. Communal nesting and kinship in degus (*Octodon degus*). Naturwissenschaften 91: 391-395.

Eisenberg, J. F., Redford, K. H. 1989. Mammals of the Neotropics. vol. 3, the central Neotropics: Ecuador, Peru, Bolivia, Brazil. University of Chicago Press, Chicago.

Eisenberg, J. F. 1974: The function and motivational basis of hystricomorph vocalizations. Symp. Zool. Soc. Lond. 34: 211-247.

Emmons, L. H., & Feer, F. 1997. Neotropical rainforest mammals. A field guide, *2*.

Francescoli, G.; Nogueira, S. and Schleich, C. 2016. Sociobiology of caviomorph rodents: an integrative view Chapter 6: Mechanisms of social communication in caviomorph rodents.

Freeberg, T. M.; Dunbar, R. I. M., & Ord, T. J. 2012. Social complexity as a proximate and ultimate factor in communicative complexity, Philos. Trans. R. Soc. B 367: 1785–1801.

Freeberg, T. M. 2006. Social Complexity Can Drive Vocal Complexity. Psychological Science 7: 557-561.

Fulk, G. 1976. Notes on activity, reproduction and social behaviour of *Octodon degus*. Journal of Mammalogy 57: 495-505.

Furrer, R. D., & Manser, M. B. 2009. The Evolution of Urgency-Based and Functionally Referential Alarm Calls in Ground-Dwelling Species. The American Naturalist 173: 400-410.

Goulart, F. V. B., Cáceres, N. C., Graipel, M. E., Tortato, M. A., Ghizoni, I. R., & Oliveira-Santos, L. G. R. 2009. Habitat selection by large mammals in a southern Brazilian Atlantic Forest. Mammalian Biology-Zeitschrift für Säugetierkunde 74: 182-190.

Griebel, U., and Oller, D. K. 2008. Evolutionary forces favoring communicative flexibility, in Evolution of Communicative Flexibility: Complexity, Creativity, and Adaptability in Human and Animal Communication, eds D. K. Oller and U. Griebel (Cambridge: MIT Press), 9–40.

Hayes L.D., Chesh, A.S., Castro, R.A. et al. 2009. Fitness consequences of group living in the degu *Octodon degus*, a plural breeder rodent with communal care. Animal Behaviour 78: 131–9.

Herrera, E. A. 2016. Caviomorphs as models for the evolution of mating systems in mammals. Sociobiology of Caviomorph Rodents: An Integrative Approach, 253-272.

Herrera, E.A., Salas, V., Congdon, E.R., Corriale, M.J. & Tang-Martínez, Z. 2011. Capybara social structure and dispersal patterns: variations on a theme. Journal of Mammalogy 92: 12–20.

Herrera, E.A. & Macdonald, D.W. 1987. Group stability and the structure of a capybara population. Symposia of the Zoological Society of London 58: 115–30.

Hrouzkova, E. 2012. Vibrational communication of subterranean rodents. PhD thesis, CZ: University of Boheminia.

Janik, V. M., & Slater, P. J. 1998. Context-specific use suggests that bottlenose dolphin signature whistles are cohesion calls. Animal behaviour 56: 829-838.

Jolliffe, I. T. 2002. Principal component analysis and factor analysis. Principal component analysis, 150-166.

Jorge, M. S., & Peres, C. A. 2005. Population Density and Home Range Size of Red Rumped Agoutis (*Dasyprocta leporina*) Within and Outside a Natural Brazil Nut Stand in Southeastern Amazonia1. Biotropica 37: 317-321.

Kleiman, D.J., Eisenberg, J.F. & Maliniak, E. 1979. Reproductive parameters and productivity of caviomorph rodents. In: Vertebrate Ecology in the Northern Tropics (J.F. Eisenberg, ed.).Smithsonian Institution Press, Washington, D.C., pp. 173–83.

Kroodsma, D. E. 1977. Correlates of song organization among North American wrens. American Naturalist, 995-1008.

Künzl, C., & Sachser, N. 1999. The behavioral endocrinology of domestication: a comparison between the domestic guinea pig (*Cavia apereaf. porcellus*) and its wild ancestor, the cavy (*Cavia aperea*). Hormones and Behavior 35: 28-37.

Lacher T. E. 1981. The comparative social behavior of *Kerodon rupestris* and *Galea spixii* and the evolution of behavior in the Caviidae. Bulletin of Carnegie Museum of Natural History 17: 1–71.

Lemasson, A., Ouattara, K., Petit, E. J., & Zuberbühler, K. 2011. Social learning of vocal structure in a nonhuman primate?. BMC Evolutionary Biology, 11, 1.

Long, C. V. 2007. Vocalisations of the degu *Octodon degus,* a social caviomorph rodent. Bioacoustics 16: 223-244.

Maher, C. R., & Burger, J. R. 2016. Diversity of social behavior in caviomorph rodents. Sociobiology of Caviomorph Rodents: An Integrative Approach, 28-58.

Manser, M. B., Jansen, D. A.,Graw, B., Hollén, L. I., Bousquet, C. A., Furrer, R. D., & le Roux, A. (2014). Vocal complexity in meerkats and other mongoose species. Advances in the Study of Behavior 46: 281.

Mares, M.A. & Ojeda, R.A. 1982. Patterns of Diversity and Adaptation in South American Hystricognath Rodents. Pymatuning Laboratory of Ecology, Special Publication Series 6, pp. 393–431.

McComb, K. & Semple, S. 2005. Coevolution of vocal communication and sociality in primates. Biology Letters 1: 381–385.

Mohlis, C. 1983. Información preliminar sobre la conservación y manejo de la chinchilla Silvestre en Chile. Corporación Nacional Forestal, Boletín Técnico (Chile) 3: 1–41.

Mones, A. & Ojasti, J. 1986. *Hydrochoerus hydrochaeris*. Mamm. Species 264: 1—7.

Monticelli, P. F. e Ades, C. 2013. The rich acoustic repertoire of a precocious rodent, the wild cavy *Cavia aperea* , Bioacoustics: The International Journal of Animal Sound and its Recording 22: 49-66

Monticelli, P. F. 2005. Comportamento e comunicação acústica em cobaias e preás. Departamento de Psicologia Experimental. São Paulo, Universidade de São Paulo: 161.

Morton, E. S. 1977. On the occurrence and significance of motivational-structural rules in some bird and mammal sounds. Am. Nat. 111: 855–869.

Nowak, R. M. 1999. Walker’s mammals of the world. Johns Hopkins University Press, London, United Kingdom.

Ojeda, R. A., Ojeda, A. A. and Novillo, A. 2016. The caviomorph rodents, in Sociobiology of Caviomorph Rodents: An Integrative Approach (eds L. A. Ebensperger and L. D. Hayes), John Wiley & Sons, Ltd, Chichester, UK.

Ord, T. J., & Garcia-Porta, J. 2012. Is sociality required for the evolution of communicative complexity? Evidence weighed against alternative hypotheses in diverse taxonomic groups. Phil. Trans. R. Soc. B 367: 1811-1828.

Pavia, A. 2003. Chinchillas: a new owners guide. Neptune City, NJ: T.F.H. Publications.

Pérez, E.M. 1992. *Agouti paca*. American Society of Mammalogists, Mammalian Species 404: 1–7.

Pollard, K. A., & Blumstein, D. T. 2012. Evolving communicative complexity: insights from rodents and beyond. *Phil. Trans. R. Soc. B* 367: 1869-1878.

Pollard, K. A., & Blumstein, D. T. 2011. Social group size predicts the evolution of individuality. Current Biology 21: 413-417.

Quirici, V., Faugeron, S., Hayes, L. D., & Ebensperger, L. A. 2010. Absence of kin structure in a population of the group-living rodent *Octodon degus*. Behavioral Ecology arq 196.

Redford, K. H., Eisenberg, J. F. 1992. In: Mammals of the Neotropics: The Southern Cone, vol. 2. University of Chicago Press, Chicago, IL.

Reig, O. A. 1970. Ecological notes on the fossorial octodont rodent *Spalacopus cyanus* (Molina). Journal of Mammalogy 51: 592-601.

Rood, J.P. 1972. Ecological and behavioural comparisons of three genera of Argentine cavies. Animal Behaviour Monographs 5: 1–83.

Sachser, N., E. Schwarz-Weig, A. Keil, And J. T. Epplen. 1999. Behavioural strategies, testis size and reproductive success in two caviomorph rodents with different mating systems. Behaviour 136: 1203–1217.

Sachser, N. 1998. Of domestic and wild guinea pigs: studies in sociophysiology, domestication, and social evolution. Naturwissenschaften 85: 307-317.

Sachser, N. 1986. Different forms of social organization at high and low population densities in guinea pigs. Behaviour 97: 252–272.

Salas, V. 1999. Social organisation in capybaras, *Hydrochoerus hydrochaeris*. Venezuela. Ph. D. thesis, Cambridge University, Cambridge.

Schleich, C. E. & Busch, C. 2002. Acoustic signals of a solitary subterranean rodent *Ctenomys talarum* (Rodentia: ctenomyidae): physical characteristics and behavioural correlates. J. Ethol. 20: 123-131.

Silvius, K. M., & Fragoso, J. 2003. Red-rumped Agouti (*Dasyprocta leporina*) home range use in an Amazonian forest: Implications for the Aggregated Distribution of Forest Trees. Biotropica 35: 74-83.

Smythe, N. & Brown de Guanti, O. 1993. La domesticación y cría de la paca *(Agouti paca).* Smithsonian Tropical Research Institute, Panamá.

Smythe, N. 1987. The paca (*Cuniculus paca*) as a domestic source of protein for the neotropical, humid lowlands. Applied Animal Behaviour Science 17: 155-170.

Snowdon, C. T., & Elowson, A. M. 1999. Pygmy marmosets modify call structure when paired. Ethology 105: 893-908.

Snowdon, C. T., A. M. Elowson, and R. S. Roush 1997. Social Influences on Vocal Development in New World Primates. In Social Influences on Vocal Development, C. Snowdon and M. Hausberger, eds. Pp. 234–248. Cambridge: Cambridge University Press.

Soto-Gamboa, M., Villalón, M., & Bozinovic, F. 2005. Social cues and hormone levels in male *Octodon degus* (Rodentia): a field test of the challenge hypothesis. Hormones and behavior 47: 311-318.

Sousa-Lima, R. S., Paglia, A. P., & da Fonseca, G. A. 2008. Gender, age, and identity in the isolation calls of Antillean manatees (*Trichechus manatus manatus*). Aquatic mammals, 34, 109.

Sousa-Lima, R. S., Paglia, A. P., & Da Fonseca, G. A. 2002. Signature information and individual recognition in the isolation calls of Amazonian manatees, *Trichechus inunguis* (Mammalia: Sirenia). Animal Behaviour 63: 301-310.

StatSoft, S. v. 7. 1. 2005. StatSoft. Computer software.

Stirling, I., & Thomas, J. A. 2003. Relationships between underwater vocalizations and mating systems in phocid seals. Aquatic Mammals 29: 227-246.

Suzuki, C. T. 2016. A complexidade do repertório acústico das capivaras *(Hydrochoerus hydrochaeris)*. Master's Dissertation, Faculdade de Filosofia, Ciências e Letras de Ribeirão Preto, University of São Paulo, Ribeirão Preto. Retrieved 2016-10-28, from http://www.teses.usp.br/teses/disponiveis/59/59134/tde-15042016-105721/

Tasse, J. 1986. Maternal and paternal care in the rock cavy, *Kerodon rupestris*, a South American hystricomorph rodent. Zoo biology *5*: 27-43.

Urrejola, D., Lacey, E. A., Wieczorek, J. R., & Ebensperger, L. A. 2005. Daily activity patterns of free-living cururos (*Spalacopus cyanus*). Journal of Mammalogy 86; 302-308.

Vanderlip, S. L. 2006. The chinchilla handbook. Hauppauge, N. Y.: Barron's.

Veitl, S., Begall, S., & Burda, H. 2000. Ecological determinants of vocalisation parameters: the case of the coruro *Spalacopus cyanus* (Octodontidae), a fossorial social rodent. Bioacoustics 11: 129-148.

Weir, B. J. 1974. Reproductive characteristic of hystricomorph rodents. In Weir BJ & IW Rowlands The Biology of Hystricomorph Rodents. Academic Press, New York: 269-299.

Wilkinson, G. S. 2003. Social and vocal complexity in bats.In Animal social complexity: intelligence, culture and individualized societies de Waal F.B.M, Tyack P.L 2003pp. 322–341. Eds. Cambridge, MA:Harvard University Press.

Wilson, E. 2000. Sociobiology: The New Synthesis, 25th Anniversary ed.(The Belknap Press of Harvard University Press, Cambridge, MA), 697 pp.

Woods, C. A. 1984. Hystricognath rodents. In: Anderson S. & Jones JR. J. K. (Eds). Orders and families of recent mammals of the world. New York: John Wiley & Sons, 389-446.

Zenuto, R. R., Vassallo, A. I., & Busch, C. 2002. Comportamiento social y reproductivo del roedor subterráneo solitario *Ctenomys talarum* (Rodentia: Ctenomyidae) en condiciones de semicautiverio. Revista chilena de historia natural *75*: 165-177.

Zenuto, R. R., Lacey, E. A., & Busch, C. 1999. DNA fingerprinting reveals polygyny in the subterranean rodent *Ctenomys talarum*. Molecular Ecology *8*; 1529-1532.

2. CONSIDERAÇÕES FINAIS

O presente estudo contribui para conhecimento da comunicação acústica de duas espécies de caviomorfos mantidas em cativeiro, a paca (*Cuniculus paca*) e a cotia (*Dasyprocta punctata*), com o fornecimento de informações sobre os parâmetros acústicos de ambas as espécies. Os resultados encontrados reforçam a compreensão de características sociais e ecológicas descritas na natureza sobre as espécies, relacionadas com à manutenção e defesa do território e complexidade vocal. Além disso, esta tese corrobora com a hipótese da complexidade social, apresentando a existência da relação entre complexidade social e vocal em dez espécies de caviomorfos, contribuindo para caracterizar os fatores sociais e não sociais que estão relacionados à complexidade vocal nessas espécies.

3. REFERÊNCIAS BIBLIOGRÁFICAS

Ades, C., Tokumaru, R. S.; Beisiegel, B. M. 1994. Vocalizações antecipatórias da cobaia *Cavia porcellus* em situação de alimentação. Biotemas 7 : 79-93.

Adkins, R. M., Gelke, E. L.; Rowe, D.; Honeycutt, R. L. 2001. Molecular phylogeny and divergence time estimates for major rodent groups: evidence from multiple genes. Mol. Biol. Evol. 18:777–791.

Alencar, R. N. Jr. 2012. O repertório acústico de um especialista de rochedos da caatinga, o mocó [dissertação de mestrado]. São Paulo: Departamento de Psicologia Experimental, Universidade de São Paulo.

Anderson, S.; Jones Jr, J. K. 1984. Orders and families of recent mammals of the world. John Wiley & Sons, New York.

Asher, M.; Lippmann, T.; Epplen, J. T.; Kraus, C.; Trillmich, F.; Sachser, N. 2008. Large males dominate: ecology, social organization, and mating system of wild cavies, the ancestors of the guinea pig. Behavioral Ecology and Sociobiology 62:1509–1521.

Asher, M.; Oliveira, E. S.; Sachser, N. 2004. Social system and spatial organization of wild guinea pigs (*Cavia aperea*) in a natural low density population. Journal of Mammalogy 85:788–796.

Asher, M.; Sachser, N. 2001. Habitat use and structure of wild guinea pigs under natural conditions. Advances in Ethology 36:117.

Azcarate, T. 1980. Sociobiologia y manejo del capibara (*Hydrochoerus hydrochaeris*). Donana Acta Vertebrata 7: 1-228.

Barros, K. S.; Tokumaru, R. S.; Pedroza, J. P. E.; Nogueira, S. S. D. C. 2011. Vocal repertoire of captive capybara (*Hydrochoerus hydrochaeris*): structure, context and function. Ethology 117: 83-93.

Bartl, J. 2006. Lautäußerungen der Chinchillas im Sozialverband (Doctoral dissertation, lmu).

Beck–King, H.; Helversen, O. V.; Beck–King, R. 1999. Home range, population density, and food resources of *Agouti paca* (Rodentia: Agoutidae) in Costa Rica: a study using alternative Methods1. Biotropica 31: 675-685.

Begall, S.; Gallardo, M. H. 2000. *Spalacopus cyanus*: an extremist in tunnel constructing and food storing among subterranean mammals. J. Zool. 251: 53–60.

Berryman, J. C. 1976. Guinea pig vocalizations, their structure, causation and function. Zeitschrift fu¨r Tierpsychologie 41:80–106.

Blumstein, D. T.; Armitage, K. B. 1997. Does sociality drive the evolution of communicative complexity? A comparative test with ground-dwelling sciurid alarm calls. American Naturalist 150: 179–200.

Braun, K.; Kremz, P.; Wetzel, W.; Wagner, T.; Poeggel, G. 2003. Influence of parental deprivation on the behavioral development in Octodon degus: modulation by maternal vocalizations. Developmental Psychobiology 42:237–245.

Busch, C.; Malizia, A. I.; Scaglia, O. A.; Reig, O. A. 1989. Spatial distribution and attributes of a population of *Ctenomys talarum* (Rodentia: Octodontidae). J Mammal 70:204–208.

Cabrera, A. 1961. Catalogo de los mamiferos de America del Sul. Revista do museu argentino das ciências naturais Bernardino Rivadavias, 4: 1-724.

Collet, S. F. Population characteristics of *Agouti paca* (rodentia) in colombia. Biol. Series, v.5, p.601, 1981.

Corbet, G. B.; Hill, J. E. 1991. A world list of mammalian species. 3rd edition. Oxford University Press, Oxford, U.K.

Crockford, C.; Boesch, C. 2005. Call combinations in wild chimpanzees. Behaviour 142: 397–421.

Devillard, S.; Allainé, D.; Gaillard, J. M.; Pontier, D. 2004. Does social complexity lead to sex-biased dispersal in polygynous mammals? A test on ground-dwelling sciurids. Behavioral Ecology 15: 83-87.

Dubost, G. 1988. Ecology and social life of the red acouchy, *Myoprocta exilis*; comparison with the orange- rumped agouti, *Dasyprocta leporina*. Journal of Zoology 214: 107-123.

Ebensperger, L. A ; Hayes, L. D. 2016. Sociobiology of caviomorph rodents: an integrative approach (eds L. A. Ebensperger and L. D. Hayes), John Wiley & Sons, Ltd, Chichester, UK. First edition, pp. 380.

Eisenberg, J. F. 1989. Mammals of the Neotropics: the northern Neotropics. University of Chicago Press, Chicago, Illinois.

Eisenberg, J. F. 1974. The function and motivational basis of hystricomorph vocalizations. Symposia of the Zoological Society of London 34:211–247.

Emmons, L. H.; Feer, F. 1997. Neotropical rainforest mammals. A field guide, 2.

Emmons, L.; Reid, F. 2016. *Dasyprocta leporina*. The IUCN Red List of Threatened Species 2016: e.T89497102A22197762. http://dx.doi.org/10.2305/IUCN.UK.2016-2.RLTS.T89497102A22197762.en. Downloaded on 01 November 2016.EMMONS, L. H., & FEER, F. (1997). Neotropical rainforest mammals. A field guide, 2.

Francescoli, G.; Nogueira, S.; Schleich, C. 2016. Sociobiology of caviomorph rodents: an integrative view Chapter 6: Mechanisms of social communication in caviomorph rodents.

Francescoli, G. 1999. A preliminary report on the acoustic communication in *Uruguayan Ctenomys* (Rodentia: Octodontidae): basic sound types. Bioacoustics 10:203–218

Freeberg, T. M., Dunbar, R. I. M.; Ord, T. J. 2012. Social complexity as a proximate and ultimate factor in communicative complexity. Philosophical Transactions of Royal Society B 367: 1785–1801.

Freeberg, T. M.; Harvey, E. M. 2008. Group size and social interactions are associated with calling behavior in Carolina chickadees, *Poecile carolinensis*. J. Comp. Psychol. 122: 312–318. (doi:10.1037/0735-7036.122.3.312)

Freeberg, T. M. 2006. Social Complexity Can Drive Vocal Complexity. Psychological Science 7: 557-561.

Fulk, G. 1976. Notes on activity, reproduction and social behaviour of *Octodon degus*. Journal of Mammalogy 57: 495-505

Guimarães, D. A.; Bastos, L. V.; Ferreira, A. C. S.; Luz-Ramos, R. S.; Ohashi, O. M.; Ribeiro, H. L. 2008. Reprodutive characteristcs of the female paca (*Agouti paca*) raised in captivity. Acta Amazônica 38: 531-538.

Hartenberger, J. L. 1985. The order Rodentia: major questionson their evolutionary origin, relationships and suprafamilial systematics. Ser. A Life Sci. 92:1–33.

Herrera, E. A. 2016. Caviomorphs as models for the evolution of mating systems in mammals. Sociobiology of Caviomorph Rodents: An Integrative Approach ,253-272.

Herrera, E. A.; Macdonald, D.W. 1993. Aggression, dominance, and mating success among capybara males (*Hydrochoerus Hydrochaeris*). Behavioral Ecology 4:114–119.

Herrera, E. A.; Macdonald, D. W. 1989. Resource utilization and territoriality in group-living capybaras (*Hydrochoerus hydrochaeris*). The Journal of Animal Ecology, 58: 667-679.

Herrera, E. A.; Macdonald, D. W. 1987. Group stability and the structure of a capybara population. Symp. Zool. Soc. Lond. 5: 115—130.

Huchon, D. E.; Douzery, J. P. 2001. From the Old World to the New World: a molecular chronicle of the phylogenyand biogeography of hystricognath rodents. Mol. Phylogenet. Evol. 20:238–251.

Huchon, D.; Catzeflis, F. M.; Douzery E. J. P. 2000. Variance of molecular datings, evolution of rodents,and the phylogenetic affinities between Ctenodactylidae and Hystricognathi. Proceedings of the Royal Society of London,Series B 276:393– 402.

Jorge, M. S.; Peres, C. A. 2005. Population Density and Home Range Size of Red Rumped Agoutis (*Dasyprocta leporina*) Within and Outside a Natural Brazil Nut Stand in Southeastern Amazonia1. Biotropica, 37: 317-321.

Krams, I.; Krama, T.; Freeberg, T. M.; Kullberg, C.; Lucas, J. R. 2012. Linking social complexity and vocal complexity: a parid perspective. Philosophy Transaction of Royal Society B 367: 1879–1891.

Lacher, T. E. JR. 1981. The comparative social behavior of *Kerodon rupestris* and *Galea spixii* and the evolution of behavior in the Caviidae. Bulletin of Carnegie Museum Natural History 17:5–71.

Lavocat, R. 1973. Les rongeurs du Miocene d’Afrique Orientale. Mémoires et Travaux de l’Institut de Montpellier de l’École Pratique des Hautes Études, Institut de Montpellier 1:1–284.

Lemasson, A.; Mikus, M-A.; Blois-Heulin, C.; Lod, E, T. 2014. Vocal repertoire, individual acoustic distinctiveness, and social networks in a group of captive Asian small-clawed otters (*Aonyx cinerea*), J. Mammal. 95: 128–139.

Le Roux, A.; Cherry, M. I.; Manser, M. B. 2009. The vocal repertoire in a solitary foraging carnivore, *Cynictis penicillata*, may reflect facultative sociality. Naturwissenschaften, 96: 575-584.

Leuchtenberger, C.; Sousa-Lima, R.; Duplaix, N.; Magnusson, W. E.; Mourão, G. 2014. Vocal repertoire of the social giant otter. The Journal of the Acoustical Society of America, 136: 2861-2875.

Lobão, É. D. S. P.; Nogueira-Filho, S. L. G. Human-wildlife Conflicts in the Brazilian Atlantic Forest. Suiform Soundings, 14.

Long, C. V. 2007. Vocalisations of the degu *Octodon degus*, a social caviomorph rodent. Bioacoustics, 16: 223-244.

Luckett, W. P.; Hartenberger, J. L. 1993. Monophyly or Polyphyly of the Order Rodentia: possible conflict between morphological and molecular interpretations. Journal of Mammalian Evolution 1: 127-47.

Maher, C. R.; Burger, J. R. 2016. Diversity of social behavior in caviomorph rodents. Sociobiology of Caviomorph Rodents: An Integrative Approach, 28-58.

Manser, M. B. 2001. The acoustic structure of suricates' alarm calls varies with predator type and the level of response urgency. Proceedings of the Royal Society of London B: Biological Sciences 268: 2315-2324.

Mares, M. A.; Ojeda, R. A. 1982. Patterns of diversity and adaptation in South American hystricognath rodents. Pp. 393–432 in Mammalian biology in South America (M. A. Mares and H. H. Genoways, eds.). Pymatuning Laboratory of Ecology, Special Publications No. 6, Pittsburgh, Pennsylvania.

Matamoros, Y. 1982. Notas sobre la biologıa del tepezcuinte, *Cuniculus paca*, Brisson (Rodentia: Dasyproctidae) en cautiverio. Brenezia 19/20, 1–82.

Mccomb, K.; Semple, S. 2005. Coevolution of vocal communication and sociality in primates. Biology Letters 1: 381–385.

Mckenna, M. C.; Bell, S. K.. 1997. Classification of mammals above the species level. Columbia University Press, New York.

Mones, A.; Ojasti, J. 1986. *Hydrochoerus hydrochaeris*. Mamm. Species 264: 1-7.

Monticelli, P. F.; Ades, C. 2013. The rich acoustic repertoire of a precocious rodent, the wild cavy *Cavia aperea*, Bioacoustics: The International Journal of Animal Sound and its Recording 22: 49-66.

Monticelli, P. F.; ADES, C. 2011. Bioacoustics of domestication: alarm and courtship calls of wild and domestic cavies. Bioacoustics 20:169–192.

Monticelli, P. F.; Tarallo, R. C. R. B.; Ades, C. 2009. Is food-anticipation whistle of domestic guinea pigs derived from isolation whistle? International Bioacoustics Congress, Lisbon.

Monticelli, P. F. 2005. Comportamento e comunicação acústica em cobaias e preás. Departamento de Psicologia Experimental. São Paulo, Universidade de São Paulo: 161.

Morton, E. S. 1977. On the occurrence and significance of motivational-structural rules in some bird and mammal sounds. Am. Nat. 111, 855–869.

Nedbal, M. A.; Allard, M. W.; Honeycutt, E. R. L. 1994. Molecular systematics of hystricognath rodents: evidencefrom the mitochondrial 12S rRNA gene. Mol. Phylogenet. Evol. 3:206–220.

Nedbal, M. A.; . Honeycutt, R. L; Schlitter, E. D. A. 1996. Higher-level systematics of rodents (Mammalia, Rodentia): evidence from the mitochondrial 12S rRNA gene.J. Mamm. Evol. 3: 201–237.

Nogueira, S. S. C.; Pedroza J. P.; Nogueira Filho, S. L. G.; Tokumaru, R. S. 2012. The function of click call emission in capybaras (*Hydrochoerus hydrochaeris*). Ethology, 118: 1-9.

Nowak, R. M. 1999. Walker's mammals of the world. Johns Hopkins University Press, London, United Kingdom.

Ord, T. J.; Garcia-Porta, J. 2012. Is sociality required for the evolution of communicative complexity? Evidence weighed against alternative hypotheses in diverse taxonomic groups. Phil. Trans. R. Soc. B 367, 1811–1828.

Patterson, B.; Wood, A. E. 1982. Rodents from the Deseadan Oligocene of Bolivia and the relationships of the Caviomorpha. Bull. Mus. Comp. Zool. 149:371–543.

Pérez, E. M. 1992. *Agouti paca*. Mammalian Species Archive, 404, 1-7.

Pollard, K. A.; Blumstein, D. T. 2012. Evolving communicative complexity: insights from rodents and beyond. Phil. Trans. R. Soc. B 367: 1869–1878.

Pollard, K. A.; Blumstein, D. T. 2011. Social group size predicts the evolution of individuality. Current Biology, 21: 413-417.

Quintana, C. A. 1998. Relaciones filogenéticas de roedores Caviinae (Caviomorpha, Caviidae), de América.Sur. Bol. R. Soc. Esp. Hist. Nat. (Sec. Biol.) 94: 125–134.

Quirici, V.; Faugeron, S.; Hayes, L. D.; Ebensperger, L. A. 2010. Absence of kin structure in a population of the group-living rodent *Octodon degus*. Behavioral Ecology, arq196.

Redford, K. H., Eisenberg, J. F., 1992. In: Mammals of the Neotropics: The Southern Cone, vol. 2. University of Chicago Press, Chicago, IL.

Rood J. P. 1972. Ecological and behavioural comparison of three genera of argentine cavies. Animal Behavior Monographs 5:1–83.

Rood J. R.; Weir B. J. 1970. Reproduction in female wild guinea-pigs. Journal of Reproduction and Fertility 23:393–409.

Rowe, D. L.; Honeycutt, R. L. 2002. Phylogenetic relationships, ecological correlates, and molecular evolution within the Cavioidea (Mammalia, Rodentia). Molecular Biology and Evolution 19: 263-77.

Sabatini, V.; Paranhos Da Costa, M. J. R. 2001b. Etograma da paca (*Agouti paca*, Linnaeus, 1766) em cativeiro. Rev. Etologia 3: 3–14.

Sachser, N.; Schwarz-Weig E.; A.; Keil, J. T. Epplen. 1999. Behavioural strategies, testis size and reproductive success in two caviomorph rodents with different mating systems. Behaviour 136:1203–1217.

Sachser, N. 1986. Different forms of social organization at high and low population densities in guinea pigs. Behaviour 97: 252–272.

Schassburger, R. M. 1993. Vocal Communication in the Timber Wolf, *Canis lupus*, Linnaeus Paul Parey Sci., Berlin.

Schleich, C.; Busch, C. 2002. Acoustic signals of a solitary subterranean rodent *Ctenomys talarum* (Rodentia: Ctenomyidae): physical characteristics and behavioural correlates. Journal of Ethology, 20: 123-131.

Silk, J. B.; Alberts, S. C.; Altmann, J. 2003. Social bonds of female baboons enhance infant survival. Science, 302: 1231-1234.

Silvius, K. M.; Fragoso, J. 2003. Red rumped agouti (*Dasyprocta leporina*) home range use in an amazonian forest: Implications for the aggregated distribution of forest trees. Biotropica 35: 74-83.

Simpson, G. G. 1974. Chairman's introduction: taxonomy. Symposia of the Zoological Society of London 34: 1-6.

Smythe, N. 1987. The paca (*Cuniculus paca*) as a domestic source of protein for the neotropical, humid lowlands. Applied Animal Behaviour Science, 17: 155-170.

Smythe, N. 1978: The natural history of the Central American agouti (*Dasyprocta punctata*). Smithsonian Contributions to Zoology 257: 1-52.

Soto-Gamboa, M.; Villalón, M.; Bozinovic, F. 2005. Social cues and hormone levels in male *Octodon degus* (Rodentia): a field test of the challenge hypothesis. Hormones and behavior 47: 311-318.

Spotorno, Á. E.; Valladares, J. P.; Marín, J. C.; Zeballos, H. 2004. Molecular diversity among domestic guinea-pigs (*Cavia porcellus*) and their close phylogenetic relationship with the Andean wild species *Cavia tschudii*. Revista Chilena de Historia Natural 77: 243-50.

Suzuki, C. T. 2016. A complexidade do repertório acústico das capivaras (*Hydrochoerus hydrochaeris*). Master's Dissertation, Faculdade de Filosofia, Ciências e Letras de Ribeirão Preto, University of São Paulo, Ribeirão Preto.

Trillmich, F. C.; Kraus, J.; Kunkele, M.; Asher, M.; Clara, G.; Dekomien, J. T.; Epplen 2004. Species-level differentiation of two cryptic species pairs of wild cavies, genera Cavia and Galea, with a discussion of the relationship between socialsystems and phylogeny in the Caviinae. Canadian Journal of Zoology 82: 516 –24.

Tullberg, T. 1899. Ueber das System der Nagethiere: Eine phylogenetischeStudie. Nova Acta Regiae Societatis Scientarium Upsaliensis 18:1–514.

Veitl, S.; Begall, S.; Burda, H. 2000. Ecological determinants of vocalisation parameters: the case of the coruro *Spalacopus cyanus* (Octodontidae), a fossorial social rodent. Bioacoustics 11: 129-148.

Wilkinson,G. S. 2003. Social and vocal complexity in bats. In Animal social complexity: intelligence, culture, and individualized societies (eds F. B. M. DeWaal & P. L. Tyack), pp. 322–341. Cambridge,MA: HarvardUniversity Press.

Wilson, E. 2000. Sociobiology: The New Synthesis, 25th Anniversary ed.(The Belknap Press of Harvard University Press, Cambridge, MA), 697 pp.

Wilson, D. E.; Reeder, D. M. 1993. Mammal species of the world. A taxonomic and geographic reference. Smithsonian Institution Press,Washington, D.C.

Wolff, J. O.; Sherman, P. W. 2007. Rodent societies: an ecological & evolutionary perspective. Edited byJerry O. Wolff and Paul W. Sherman.The University of Chicago Press, Ltd., London, 1-605

Wood, A. E. 1965. Grades and clades among rodents. Evolution19:115–30.

Woods, C. A.; Kilpatrick. 2005. Infraorder hystricognathi brandt, 1855. In D. E. Wilson & D. M. Reeder (Eds.), Mammal species of the world. A taxonomic and geographic reference. Baltmore, Maryland: Johns Hopkins University Press, pp. 1538–1600.

Woods, C. A. 1984. Hystricognath rodents. In: Anderson, S., Jones, Jr., J.K. (Eds.), Orders and Families of Recent Mammals of the World. John Wiley and Sons, Canada, pp. 389–445.

Universidade federal do Rio Grande do Norte

Centro de Biociências

Programa de Pós-graduação em Psicobiologia

Justificativa sobre o parecer de comitê de ética (Ceua)

A presente tese faz parte de um projeto maior (projeto mãe) intitulado "Uso da bioacústica como inovação tecnológica aplicada à conservação de mamíferos neotropicais no sul da Bahia." Esse projeto foi aprovado pelo comitê de ética da Universidade Estadual de Santa Cruz. Segue, portanto o certificado de aprovação para a realização das pesquisas.

Profª Drª Renata Santoro de Sousa Lima Mobley
Universidade Federal do Rio Grande do Norte

UNIVERSIDADE ESTADUAL DE SANTA CRUZ
COMISSÃO DE ÉTICA NO USO DE ANIMAIS (CEUA)

CERTIFICADO

Certificamos que o **Protocolo nº 010/11**, relativo ao projeto intitulado **Uso da bioacústica como inovação tecnológica aplicada à conservação de mamíferos neotropicais no sul da Bahia**, da **Drª. Selene Siqueira da Cunha Nogueira**, está de acordo com os Princípios Éticos da Experimentação Animal, adotados pela ***Comissão de Ética no Uso de Animais*** **(CEUA – UESC)**, e foi aprovado na reunião de **10/04/2012**.

Ilhéus, 17 de abril de 2012.

Prof. Guilherme Rosemberg G. Queiroz
Presidente do CEUA - UESC
Cád.: 73.333.462-1

Universidade Estadual de Santa Cruz – **UESC**
Campus Prof. Soane Nazaré de Andrade,
Km 16 – Rodovia Ilhéus/Itabuna – CEP: 45.662-000. Ilhéus – Bahia – Brasil
Comissão de Ética no Uso de Animais (CEUA) – Telefax.: (73) 3680-5319 – ceuauesc@gmail.com
